# Pro‐inflammatory immunity supports fibrosis advancement in epidermolysis bullosa: intervention with Ang‐(1‐7)

**DOI:** 10.15252/emmm.202114392

**Published:** 2021-08-30

**Authors:** Rocco Bernasconi, Kerstin Thriene, Elena Romero‐Fernández, Christine Gretzmeier, Tobias Kühl, Mareike Maler, Pauline Nauroy, Svenja Kleiser, Anne‐Catherine Rühl‐Muth, Michael Stumpe, Dimitra Kiritsi, Stefan F Martin, Boris Hinz, Leena Bruckner‐Tuderman, Jörn Dengjel, Alexander Nyström

**Affiliations:** ^1^ Department of Dermatology Medical Faculty Medical Center – University of Freiburg Freiburg Germany; ^2^ Faculty of Biology University of Freiburg Freiburg Germany; ^3^ Department of Biology University of Fribourg Fribourg Switzerland; ^4^ Laboratory of Tissue Repair and Regeneration Faculty of Dentistry University of Toronto Toronto ON Canada; ^5^ Freiburg Institute for Advanced Studies (FRIAS) University of Freiburg Freiburg Germany; ^6^ University Hospital Regensburg Regensburg Germany; ^7^ Essen University Hospital Essen Germany

**Keywords:** collagen VII, genetic disease, inflammation, skin, therapy, Genetics, Gene Therapy & Genetic Disease, Immunology, Skin

## Abstract

Recessive dystrophic epidermolysis bullosa (RDEB), a genetic skin blistering disease, is a paradigmatic condition of tissue fragility‐driven multi‐organ fibrosis. Here, longitudinal analyses of the tissue proteome through the course of naturally developing disease in RDEB mice revealed that increased pro‐inflammatory immunity associates with fibrosis evolution. Mechanistically, this fibrosis is a consequence of altered extracellular matrix organization rather than that of increased abundance of major structural proteins. In a humanized system of disease progression, we targeted inflammatory cell fibroblast communication with Ang‐(1‐7)—an anti‐inflammatory heptapeptide of the renin‐angiotensin system, which reduced the fibrosis‐evoking aptitude of RDEB cells. *In vivo,* systemic administration of Ang‐(1‐7) efficiently attenuated progression of multi‐organ fibrosis and increased survival of RDEB mice. Collectively, our study shows that selective down‐modulation of pro‐inflammatory immunity may mitigate injury‐induced fibrosis. Furthermore, together with published data, our data highlight molecular diversity among fibrotic conditions. Both findings have direct implications for the design of therapies addressing skin fragility and fibrosis.

## Graphical abstract

Injury‐evoked fibrosis in recessive dystrophic epidermolysis bullosa (RDEB) associates with inflammatory immunity. This fibrosis is a consequence of altered extracellular matrix (ECM) organization. Targeting fibro‐inflammatory communication with Ang‐(1‐7) effectively attenuates fibrosis in RDEB.

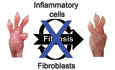

**The paper explained**

**Problem**

Fibrotic diseases constitute a huge medical challenge in that they commonly contribute to organ failure and death. Despite the severity of the problem, the current treatment options for fibrotic diseases are limited, since the disease mechanisms are not completely understood. The efficacy of the few targeted and more general therapies remains low for most conditions and comes with heavy side effects.

**Results**

Using recessive dystrophic epidermolysis bullosa (RDEB) as a paradigmatic model of injury‐induced fibrosis, our study discloses that disabling fibrosis in RDEB is driven by inflammatory immunity. Informed targeting of this inflammation with the natural anti‐inflammatory peptide Ang‐(1‐7) effectively limits fibrosis in RDEB.

**Impact**

Our study suggests that fibrosis in RDEB is the consequence of dysregulation of relatively few and selected biological processes and, consequently, reduction of the intensity of these processes can provide efficient protection against fibrosis development. These findings will not only be directly applicable for RDEB but also for the design of safe and tolerable therapies against other fibrotic diseases.

## Introduction

Tissue injury and inflammation are major triggers that promote fibrosis—the replacement of a functional parenchyma with disorganized scar tissue (Eming *et al*, 2014; Kim *et al*, 2019). Diseases associated with tissue destabilization, such as acquired or genetically‐evoked faulty extracellular matrix (ECM) assembly, frequently present with progressive fibrosis (Nyström & Bruckner‐Tuderman, 2018). A paradigmatic disorder in this context is recessive dystrophic epidermolysis bullosa (RDEB), a rare genetic skin blistering disease caused by mutations in *COL7A1* encoding collagen VII (Cianfarani *et al*, 2017). In the skin, collagen VII is deposited below the epidermal basement membrane where it assembles into electron‐dense suprastructures—anchoring fibrils—and attaches the basement membrane to the underlying dermal ECM. Collagen VII deficiency leads to chronic skin fragility and progressive multi‐organ fibrosis (Nyström & Bruckner‐Tuderman, 2018). Unremitting skin blistering and wounding facilitate development of a severely debilitating dermal fibrosis that causes fusion of fingers and toes and formation of so‐called mitten deformities. In addition, heavily fibrotic sites establish a microenvironment supportive of development and progression of high‐risk cutaneous squamous cell carcinoma at young age (Cho *et al*, 2018). With advancing course of RDEB, fibrosis becomes systemic and affects many organs including the gastrointestinal tract and the eyes.

In RDEB, it is apparent that mechanical injury expedites fibrosis, since exposed sites are first affected. However, scarce knowledge exists about the specific composition of the fibrosis and cellular and molecular triggers promoting its evolution. Although we have shown by using losartan or decorin that targeting TGFβ‐related mechanisms activated downstream of tissue injury can limit fibrosis and reduce severity of the disease in RDEB model mice (Nyström *et al*, 2015; Cianfarani *et al*, 2019), it is likely that other mechanisms contribute to the evolution of fibrosis as well. Mass spectrometry (MS)‐based proteomic analysis of limitedly affected back skin from adult wild‐type (WT) mice or RDEB model mice treated with or without losartan revealed that losartan‐treatment altered some proteins linked to tissue inflammation (Nyström *et al*, 2015). However, the involvement of these proteins as well as other processes outside the response to losartan, i.e., during establishment of fibrosis and advancement of disease, were not assessed. Knowledge of such mechanisms is pivotal for the development of efficacious fibrosis‐limiting therapies that will not only alleviate progression of fibrosis but also improve the outcome of curative cell‐based treatments by generating a less stiff and chronically damaged tissue microenvironment for the therapeutic cells (Nyström & Bruckner‐Tuderman, 2016).

A well‐regulated inflammatory response is essential for correct repair after injury (Eming *et al*, 2017). It is a fine‐tuned event in which pro‐inflammatory immunity should transition into tissue repair immunity, which should subside in a timely manner; however, it is becoming increasingly evident that classification of inflammatory responses is fuzzy (Jeljeli *et al*, 2019). Dysregulation of inflammatory events commonly underlies wound healing pathologies, from chronic wounds to fibrosis (Eming *et al*, 2017). RDEB can be considered a wound healing pathology, and indeed analyses of skin wounds, dressings, and sera from people with RDEB have revealed altered abundance and activity of immune cells and immune mediators, with some studies indicating wound type‐dependent changes (Nyström *et al*, 2015; Alexeev *et al*, 2017; Cianfarani *et al*, 2017; Fuentes *et al*, 2020; Phillips *et al*, 2020; Huitema *et al*, 2021). Rebalancing the inflammatory response downstream of chemically evoked tissue injury and inflammation can attenuate fibrosis progression in preclinical models (Jeljeli *et al*, 2019). However, the aptitude of inflammation modulation for treatment of injury‐induced fibrosis established in a natural setting remains unknown.

Prolonged targeting of inflammation with small molecule drugs frequently comes with unwanted side effects, an alternative attractive approach is to use natural anti‐inflammatory peptides (La Manna *et al*, 2018). The heptapeptide Ang‐(1‐7) is a natural peptide of the renin‐angiotensin system (RAS) with physiological anti‐inflammatory properties that can be therapeutically exploited (Regenhardt *et al*, 2013; Magalhaes *et al*, 2018). Recombinant Ang‐(1‐7) exhibited a good safety profile in preclinical models and in humans during clinical testing for various indications (Petty *et al*, 2009; Mordwinkin *et al*, 2012; Lee *et al*, 2013). Apart from targeting inflammation, on the tissue level it may also directly limit pro‐fibrotic RAS signaling in fibroblasts and parenchymal cells (Bernasconi & Nyström, 2018) and, conversely, activate fibrosis‐limiting signaling. Additionally, Ang‐(1‐7) potentiates fibrosis‐limiting signaling of the kinin‐kallikrein system (KKS) activated after injury (Su, 2014).

Here, longitudinal proteomics of an RDEB mouse model combined with clinical sample analyses showed that progression of fibrosis in RDEB is associated with pro‐inflammatory immunity. Intriguingly, histologically heavily scarred and mechanically stiffened tissue displayed no increase in abundance of major ECM proteins, the typical hallmark of fibrosis (Schaefer, 2018). Rather, fibrosis was a structural consequence of altered ECM organization. Guided by proteomics, we aimed to modulate tissue repair and inflammatory responses and tested the natural heptapeptide Ang‐(1‐7) to reduce fibrosis burden in RDEB. In patient‐derived models and in RDEB mice, low‐dose Ang‐(1‐7) evoked sustained fibrosis‐limiting activities by targeting fibrosis‐inflammatory cross‐communication. Our data support development of Ang‐(1‐7) as therapeutic agent for RDEB and other injury‐ and inflammation‐driven fibrotic diseases. They highlight molecular diversity in established fibrosis and indicate that discrete modulation of inflammation in fibrosis‐primed tissues may evoke sustained attenuation of disease progression.

## Results

### Injury and inflammation correlate with dermal fibrosis in RDEB

**Figure 1:**
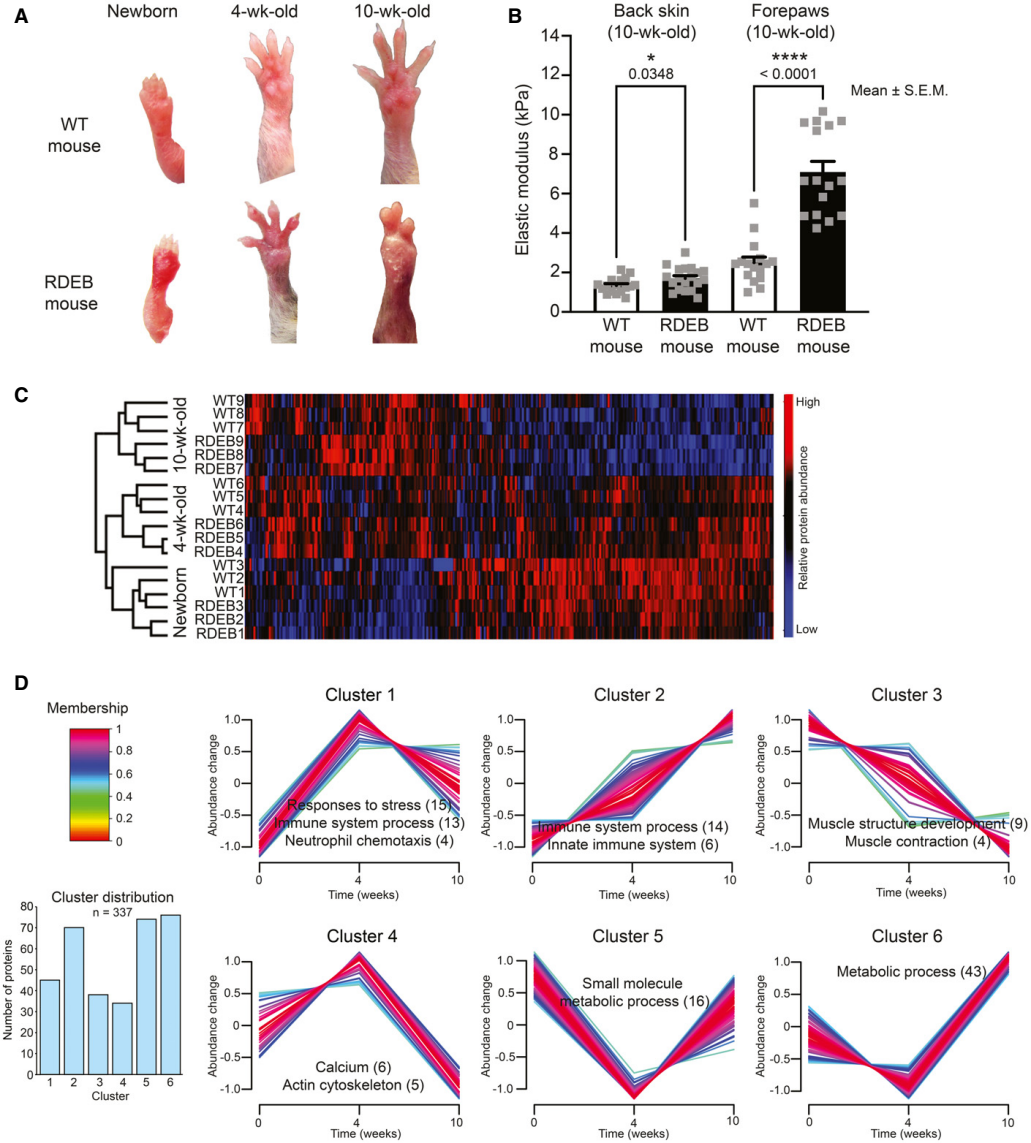
Progression of dermal fibrosis in RDEB is associated with limited changes of the proteome with inflammation being the principal dysregulated process Photographs of forepaws from WT and RDEB mice of the indicated ages. In RDEB mice, dermal fibrosis is apparent by fusion of digits and formation of mutilating deformities.The stiffness of back skin and forepaw palms from 10‐wk‐old WT and RDEB mice was measured with a micro‐indentation stiffness measurement tool. *N* = 3 mice per genotype and 5–7 measurements per mouse. Individual data points, mean ± SEM, are shown, *P* = 0.0348 (back skin) and *P* < 0.0001 (forepaws) tested with unpaired *t*‐test.Hierarchical clustering of forepaw samples of the indicated ages and genotypes based on protein abundances determined by label‐free quantification MS analysis. Limited differences are seen between age‐matched WT and RDEB mice, and most notable differences are between ages.Fuzzy c means clustering of significantly altered proteins in paws of RDEB compared to WT mice to visualize their dynamic regulation during disease progression. Average abundance differences of three biological replicates each (RDEB/WT) were log2‐transformed and standardized. Shown are relative abundance changes over time (0, 4, and 10 weeks). Six clusters were generated, and each cluster was analyzed for enrichment of pathways and processes using STRING DB (Szklarczyk *et al*, 2019). Representative pathways are listed for each cluster. The number of proteins belonging to each pathway is indicated in brackets.

**Figure EV1:**
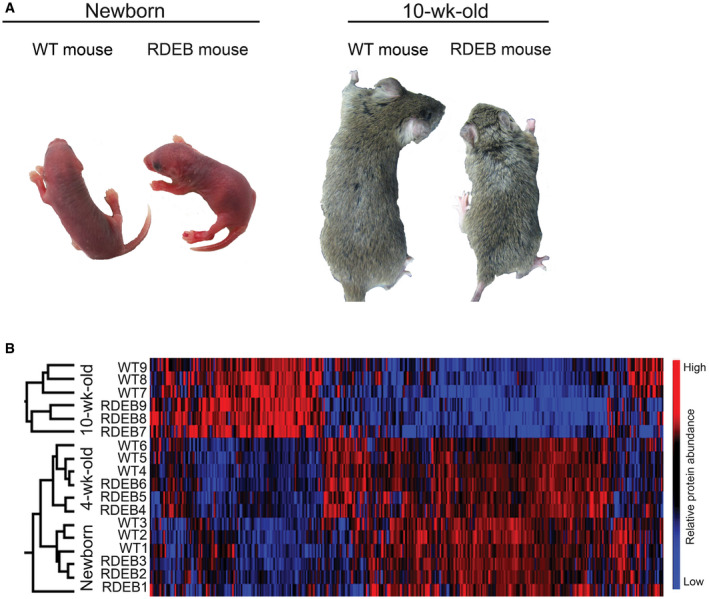
RDEB mouse back skin does not display overt injury or subsequent fibrosis Photographs of back skin from WT and RDEB mice of the indicated ages.Hierarchical clustering of back skin samples of the indicated ages and genotypes based on protein abundances determined by label‐free quantification MS analysis. Limited differences are seen between age‐matched WT and RDEB mice, and most notable differences occur between ages.

The collagen VII hypomorphic (RDEB) mouse model replicates all manifestations of human RDEB (Fritsch *et al*, 2008; Nyström *et al*, 2018), including progressive formation of fibrotic mitten deformities (Fig 1A). Indentation measurements of the skin revealed that fibrotic deformation of paws corresponds to significantly increased dermal stiffness (Fig 1B). Mechanical challenges appear to expedite fibrosis, since areas meeting few frictional challenges, such as the fur‐covered back skin, showed no overt macroscopic or molecular signs of fibrosis (Fig EV1A and (Nyström *et al*, 2015)) and compared to forepaws lesser increase in stiffness (Fig 1B).

To delineate molecular and cellular processes underlying progressive fibrosis in RDEB, we performed MS‐based proteomics of protein lysates from forepaw and back skin of RDEB mice vis‐à‐vis WT littermates. Three ages were selected based on the phenotypic presentation of forepaws: newborns—representing initial injury, 4‐week‐old—representing mid‐stage fibrosis, and 10‐week‐old—representing advanced fibrosis (Fig 1A and (Nyström *et al*, 2015)).

Hierarchical clustering of abundances of proteins identified in all samples from each body site disclosed limited changes between genotypes; most differences appeared between ages (Figs 1C and EV1B). Together with the conspicuous progressive fibrosis of forepaws, this indicated normal postnatal development and aging of RDEB skin and suggested that fibrosis is driven by, and the result of, changes in a rather limited number of proteins and processes. To identify underlying protein changes, the same datasets were first analyzed with Linear Models for Microarray and RNA‐Seq Data (LIMMA) statistics. Newborn WT and RDEB mice could not be discriminated based on their proteomic signatures, which was consistent with the mild disease phenotype at a young age. However, with advancing disease WT and RDEB forepaw samples could be distinctly segregated highlighting significant differences of proteins participating in/contributing to disease‐relevant pathways (Dataset EV1). In contrast, back skin, which is less exposed to frictional challenges and protected by fur, showed milder progression and significant proteome changes were observed only after 10 weeks (Dataset EV2). Principal component analyses supported the observed postnatal developmental changes of the skin proteome and earlier separation between WT and RDEB in affected forepaw compared to back skin (Appendix Fig S1). Collectively, these results underline the progressive nature of fibrosis in RDEB and the importance of mechanical challenges to its propagation.

**Figure EV2:**
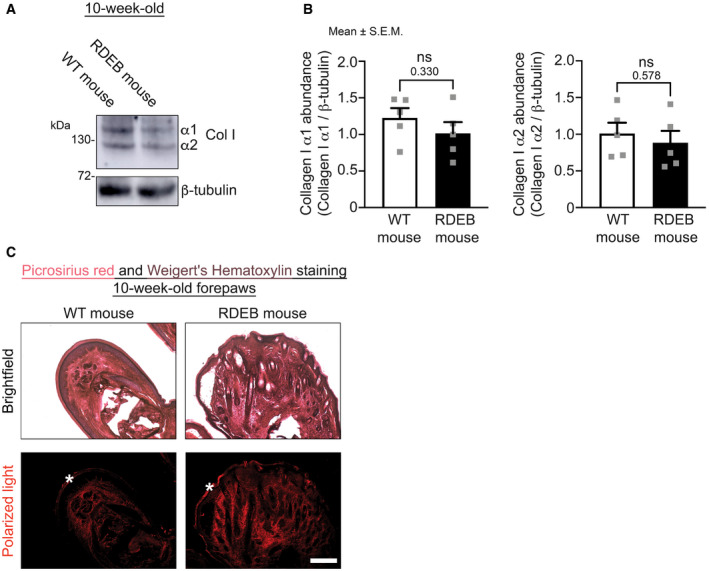
Collagen I abundance is not increased in fibrotic RDEB mouse skin but arrangement of fibrillar collagens is altered Western blot analysis of protein lysates from forepaws of 10‐wk‐old WT and RDEB mice for collagen I. β‐tubulin was used as a loading control.Densitometric quantification of collagen I α1 (left) or α2 (right) polypeptide abundance normalized to expression of β‐tubulin. *N* = 5 forepaws from 10‐wk‐old WT and RDEB mice. Individual values, mean ± SEM are shown. Data were tested with unpaired *t*‐test. *P* values are indicated, ns = not significant.Images of sections of forepaws from 10‐wk‐old WT and RDEB mice stained for picrosirius red and Weigert’s hematoxylin taken under brightfield (top, picrosirius red = pink‐red and Weigert’s hematoxylin = brown) or under polarized light (bottom). Increased red staining under polarized light indicates thickened collagen fibrils or their increased parallel arrangement. Scale bar = 100 μm. Asterisks indicate unspecific epidermal staining.

Pathway enrichment analyses of significantly altered proteins in paws indicated that the downregulated proteins in RDEB were associated with metabolic changes (Dataset EV3). A greater quantity of proteins was increased, and among those there was in 4‐week‐old and 10‐week‐old RDEB paws an enrichment of proteins associated with inflammatory pathways (Table 1 and Dataset EV3). Unexpectedly, no significant changes of major fibrillar collagens were observed in advanced fibrotic 10‐week‐old paws (Dataset EV1). Targeted analyses of collagen I by Western blotting validated the proteomic findings; they revealed no increase of collagen I (Fig EV2A and B). Instead, in the heavily deformed 10‐week‐old RDEB paws terms indicative of fibroblast activation and ECM remodeling were enriched. Upregulated individual proteins associated with these terms included collagen VI, collagen XII, PLOD2, and the transitional ECM proteins tenascin‐C and periostin (Datasets EV1 and EV3). These ECM constituents are known to promote organization and crosslinking of major structural ECM components, ultimately leading to tissue stiffening (Mölleken *et al*, 2009; Maruhashi *et al*, 2010; Bhattacharyya *et al*, 2016; Midwood *et al*, 2016; Murota *et al*, 2017). Analysis of dermal fibrillar collagen matrices by picrosirius red staining visualized under polarized light revealed strongly altered organization of collagen fibrils in fibrotic RDEB skin (Fig EV2C). Thus, fibrosis progression in RDEB is associated with altered ECM organization rather than increased bulk synthesis of major structural ECM proteins.

**Table 1:** Immune‐related pathways are among top‐regulated pathways in RDEB progression.

Term ID	Term description	Observed protein count	Background protein count	False discovery rate
A, Gene Ontology Biological Process				
Forepaws 4‐wk‐old – Proteins increased in RDEB vs. WT				
GO:0002376	Immune system process	25	1703	2.06E‐06
GO:0006952	Defense response	20	1079	2.06E‐06
GO:0052548	regulation of endopeptidase activity	13	387	2.06E‐06
GO:0051707	Response to other organism	19	1050	2.61E‐06
GO:0009605	Response to external stimulus	25	2021	7.93E‐06
GO:0019882	Antigen processing and presentation	6	63	4.50E‐05
GO:0030162	Regulation of proteolysis	14	716	7.05E‐05
GO:0098542	Defense response to other organism	14	735	8.73E‐05
Forepaws 10‐wk‐old—Proteins increased in RDEB vs. WT				
GO:0019882	Antigen processing and presentation	7	63	0.00022
GO:0002376	Immune system process	27	1703	0.00032
GO:0006950	Response to stress	36	2899	0.00033
GO:0006955	Immune response	19	914	0.00033
GO:0043603	Cellular amide metabolic process	16	644	0.00033
GO:0006952	Defense response	20	1079	0.0005
GO:0048002	Antigen processing and presentation of peptide antigen	5	34	0.00068
GO:0002474	Antigen processing and presentation of peptide antigen via MHC class I	4	20	0.002
B, Reactome Pathway				
Forepaws 4‐wk‐old—Proteins increased in RDEB vs. WT				
MMU‐168249	Innate Immune System	9	246	6.72E‐06
MMU‐1266738	Developmental Biology	9	340	4.48E‐05
MMU‐109581	Apoptosis	4	93	0.0027
Forepaws 10‐wk‐old—Proteins increased in RDEB vs. WT				
MMU‐168249	Innate Immune System	10	246	2.78E‐05
MMU‐1430728	Metabolism	19	1346	0.0012
MMU‐109582	Hemostasis	9	484	0.0106

Selection of top eight GO biological processes (A) and top three reactome pathways (B) linked to proteins with significantly increased abundance in 4‐ or 10‐week‐old RDEB compared to wild‐type mouse forepaws. For full lists, see Dataset EV3.

To understand the dynamic regulation of the processes related to dermal fibrosis, we performed fuzzy c means clustering. Abundance differences of the significantly changed proteins between RDEB and WT mice were log2‐transformed and standardized to follow relative changes of RDEB compared to WT mice. Data were split into six clusters of similar size (Fig 1D), clusters 2 and 3 containing proteins that progressively increase or decrease over time in RDEB mice, respectively. Clusters 1 and 4–6 contained proteins with more complex, bimodal abundance changes. Pathway enrichment analyses performed on the proteins within each cluster revealed a dynamic regulation of inflammation during progression of fibrosis in forepaws (Fig 1D, Dataset EV4). These data reiterated principal association of progressive changes in inflammation during fibrosis establishment in RDEB.

### Dynamic changes of inflammation during progression of dermal fibrosis

**Figure 2:**
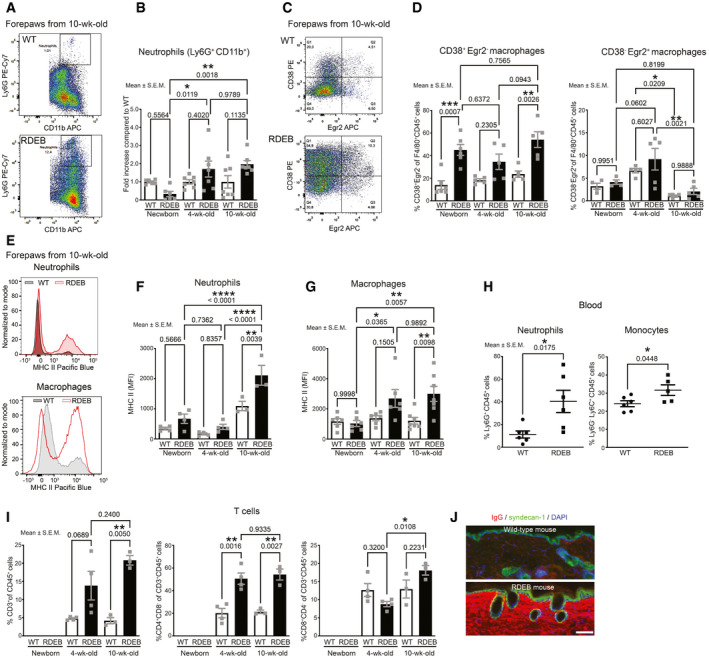
Progressively increased pro‐inflammatory immunity during the course of RDEB APlot from flow cytometry analysis of neutrophils in forepaw skin from 10‐wk‐old WT and RDEB mice.BBar graph of flow cytometry analyses as in A of neutrophils in forepaw skin of newborn, 4‐wk‐old and 10‐wk‐old WT and RDEB mice. Plotted as fold increase compared to mean of age‐matched WT paws; replicates per age = 5–6.CPlot from flow cytometry analysis of CD38^+^ and Egr2^+^ macrophages in forepaw skin from 10‐wk‐old WT and RDEB mice.DBar graph of flow cytometry analyses as in C of inflammatory macrophages (CD38^+^Egr2^−^) or tissue repair macrophages (CD38^−^Egr2^+^) macrophages in forepaw skin of newborn, 4‐wk‐old, and 10‐wk‐old WT and RDEB mice. Plotted as fold increase compared to mean of age‐matched WT paws; replicates per age; *n* = 5‐6 (CD38^+^Egr2^−^); *n* = 4–5 (CD38^−^Egr2^+^).EPlots of MHC II staining intensity in neutrophils (top) and in macrophages (bottom) from flow cytometry analysis of forepaws from 10‐wk‐old WT and RDEB mice.F, GThe mean fluorescence intensity (MFI) of MHC II staining on (F) neutrophils (*n* = 3–4 biological replicates) and (G) macrophages (*n* = 6‐7 biological replicates).HSystemic inflammation in RDEB mice. The percentage abundance of neutrophils and monocytes in blood of 4‐wk‐old WT and RDEB mice, (*n* = 5–7 mice).IAnalyses of percentage CD3ɛ^+^‐positive CD45^+^ cells (T cells) in paws, and percentage CD4 or CD8 single‐positive T cells (*n* = 3–4 biological replicates),JStaining of back skin from 10‐wk‐old WT and RDEB mice for IgG (red). Keratinocytes are stained with syndecan‐1 (green), and 4′,6‐diamidino‐2‐phenylindole (DAPI) (blue) was used to visualize nuclei, scale bar = 100 μm. Data information: Individual data points, mean ± SEM, are shown. The data were analyzed by one‐way ANOVA with Tukey’s correction (B, D, F, G, and I) or unpaired *t*‐test (H). *P* values < 0.05 are considered significant. Source data are available online for this figure.

Based on the above data, we assessed the inflammatory events that link tissue damage to fibrosis in RDEB. Toward this end, we analyzed the immune cell‐subtype composition in forepaw skin of RDEB and age‐matched WT mice by flow cytometry. Interestingly, at birth, neutrophil abundance was substantially lower in RDEB paws than in WT paws. However, with advancing age and severity of the phenotype neutrophils became significantly more abundant in RDEB (Fig 2A and B). Contrastingly to neutrophils, CD38^+^ inflammatory macrophages (F4/80^+^CD38^+^; Jablonski *et al*, 2015) were increased already in newborn RDEB mouse paw skin and remained significantly elevated in relation to WT during the course of the disease (Fig 2C and D). Notability, in the counts of tissue repair macrophages marked by Egr2 positivity (F4/80^+^Egr2^+^), only minimal differences were seen (Fig 2D). Both CD38^+^ inflammatory macrophages and neutrophils had significantly elevated MHC (major histocompatibility complex) II levels from 4 weeks onwards in RDEB paws (Fig 2E–G), indicating an increased activation status of both cell types. Enhanced recruitment and activity of inflammatory cells suggested a systemic response. Indeed, levels of neutrophils and monocytes were elevated in blood of mid‐stage RDEB mice (Fig 2H). Furthermore, analysis of bone marrow‐derived macrophages revealed increased *Tlr4* expression in cells derived from RDEB mice with advanced disease (Appendix Fig S2), indicating systemic pro‐fibrotic priming of immune cells (Jeljeli *et al*, 2019).

Heightened activity and antigen‐presenting abilities of inflammatory macrophages and neutrophils promote adaptive immunity (Buxadé *et al*, 2018). Significant elevation of T cells, specifically CD4 T cells, and deposition of tissue‐bound antibodies occurred with RDEB disease progression (Fig 2I and J, and Appendix Fig S3). Gene expression analysis of selected T‐cell and inflammatory cell markers revealed increased expression of *Tbet* (type 1 immunity), but unchanged expression of *Gata 3* (type 2 immunity) and *Rorc* (type 17 immunity) at mid‐stage and advanced stage of the disease. The mid‐stage appeared to be associated with an elevated T‐cell activation, as indicated by increased *Pdcd1* and *CD27* expression. At advanced stage disease, the increased expression of *Eomes* and continuously elevated *Pdcd1* suggested exhaustion (Minter *et al*, 2005; McLane *et al*, 2019) (Appendix Fig S4). Collectively, these results support a progressive type 1 inflammatory response in RDEB mice.

### Human RDEB skin displays progressive inflammatory changes and enhanced ECM organizer proteins

**Figure 3:**
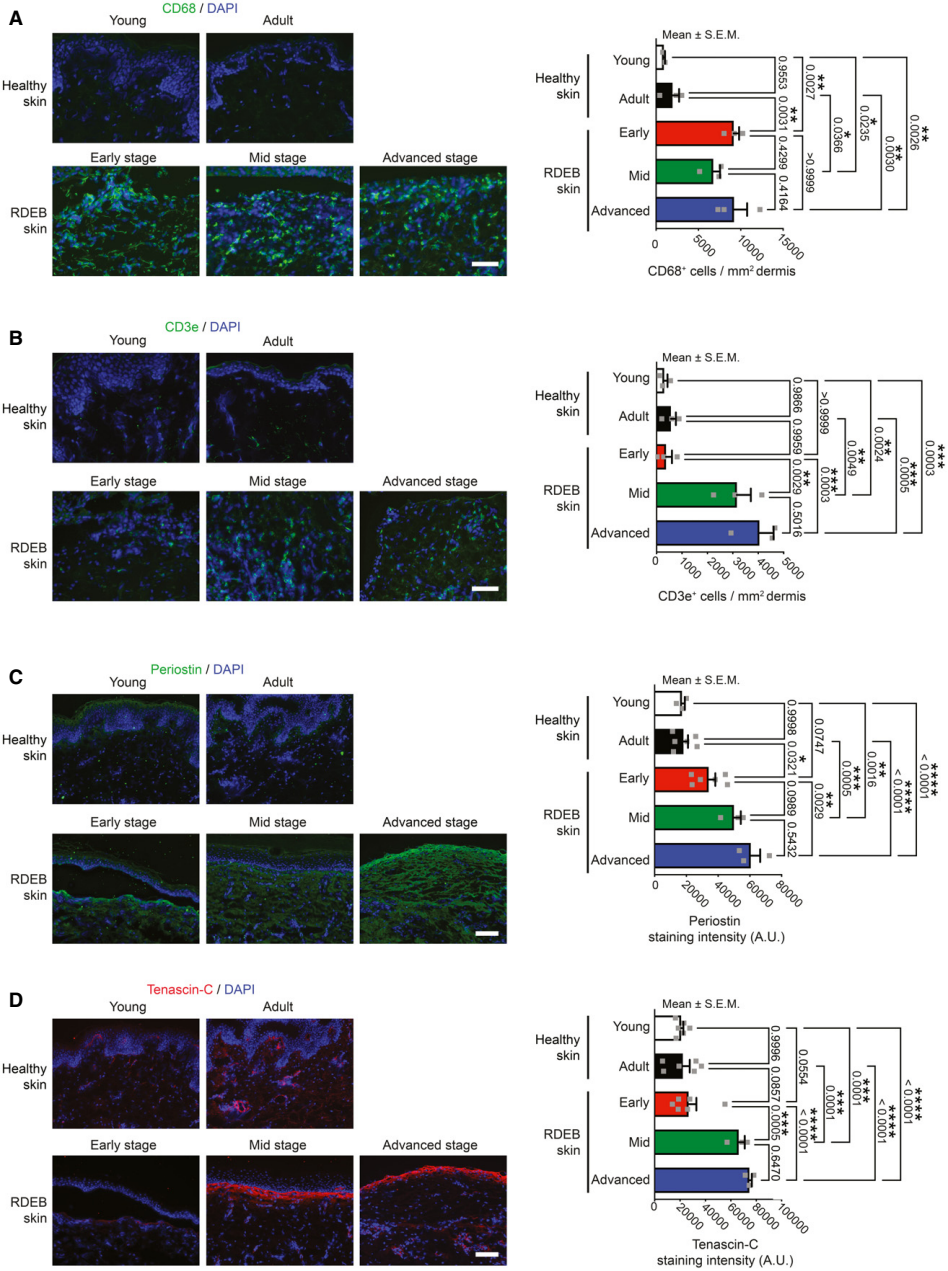
Translational validation of stage‐dependent inflammatory and fibrosis‐associated protein changes in human RDEB skin A–DSkin from healthy young (3–8 years old) and adult donors (> 25 years old) and from donors with RDEB with different stages of fibrosis—early (0‐3 years old), mild (6‐10 years old), and advanced (> 15 years old)—stained for CD68 (green), CD3ɛ (green), periostin (green), and tenascin‐C (red). Nuclei were counterstained with DAPI (blue). A and B, scale bar = 50 μm; C and D, scale bar = 100 μm. The bar graphs at the right to the stained sections, represent for A and B quantification of the number positive cells per mm^2^ analyzed from 3 donors for each group and for C and D, the mean intensity of staining from 3 to 6 donors per group. Individual data points, mean ± SEM, are shown. The data were analyzed by one‐way ANOVA with Tukey’s correction. *P* values < 0.05 are considered significant. Source data are available online for this figure.

To study fibrosis progression of RDEB in humans, we analyzed skin from RDEB donors with differently advanced fibrotic disease. Mimicking our findings in mice, human RDEB skin, compared to healthy skin, displayed dramatically elevated macrophage abundance already at an early disease stage (Fig 3A). This was followed by significantly increased numbers of T cells in advanced disease stages (Fig 3B). These findings are in line with previous analyses of human RDEB skin from undisclosed disease stages showing increase in myeloid cell and T‐cell abundance (Alexeev *et al*, 2017). Importantly, the progression of inflammation as indicated by activation of adaptive immunity correlated with fibrosis‐associated changes of the ECM (Fig 3C and D). As in mice, there was an increase in transitional ECM proteins tenascin‐C and periostin, both are active organizers of the ECM, including a fibrotic collagen matrix. Collectively, these analyses provide translational validation of the results obtained in the RDEB mouse model.

### Ang‐(1‐7) alters inflammatory cell—fibroblast communication

**Figure EV3:**
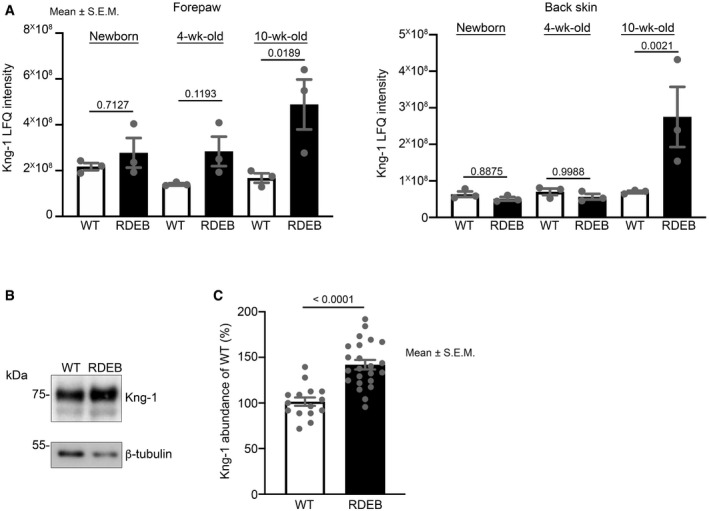
Kininogen‐1 is increased in injured RDEB mouse forepaws Plotted are intensity values of kininogen‐1 derived from label‐free quantification (LFQ) of whole back skin and forepaw protein lysates analyzed by label‐free MS‐based proteomics. Analyzed were forepaws from newborn, 4‐week‐old, and 10‐week‐old WT and RDEB mouse littermates. These ages represent initial injury, mid‐stage fibrosis, and late‐stage fibrosis. *N* = 3 per genotype and age. Statistics calculated by LIMMA and the resulting *P* values are shown. Individual data points, mean ± SEM, are shown.Validation of proteomics by Western blotting. Representative Western blots for kininogen‐1 of forepaw protein lysates from 10‐week‐old WT and RDEB mice are shown. The blots were probed with β‐tubulin as a loading control.Densitometric quantification of Western blots as in B. Kininogen‐1 abundance was normalized to β‐tubulin and expressed as percentage abundance in paired WT samples. Individual data points, mean ± SEM, are shown, *n* = 8 forepaws from 8 different mice per genotype. *P* < 0.0001 (unpaired *t*‐test).

**Figure EV4:**
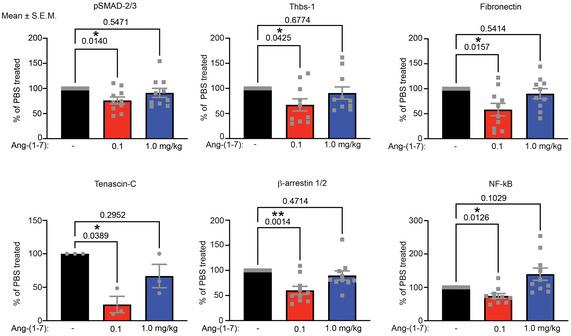
Low dose of Ang‐(1‐7) significantly alters abundances of proteins important for dermal homeostasis Densitometric quantification of Western blots shown in Fig 7C of forepaw lysates from RDEB mice treated with daily injections of 0.1, 1.0 mg/kg Ang‐(1‐7), or PBS for seven weeks. Quantifications after normalization to β‐tubulin or β‐actin for pSMAD‐2/3 (pSer 465/467 SMAD‐2/pSer 423/425 SMAD‐3), thrombospondin‐1 (Thbs‐1), fibronectin, tenascin‐C, β‐arrestin‐1/2, and NF‐κB are shown. Individual data points from individual mice, mean ± SEM, are shown. Data are expressed as the percentage abundance of PBS‐treated and were analyzed by one‐way ANOVA with Dunnett’s correction. *P* values < 0.05 are considered significant. *N* = 6–8 paws (one paw per mouse) per condition.

**Figure EV5:**
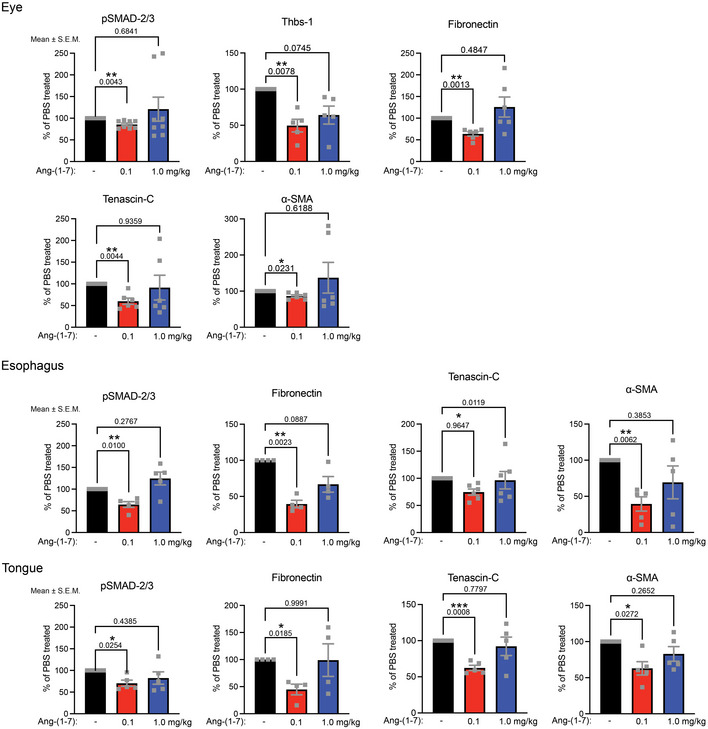
Low dose of Ang‐(1‐7) significantly alters abundances of proteins important for homeostasis of eye, esophagus and tongue Densitometric quantification of Western blots shown in Fig 7E of whole eye, esophagus, and tongue lysates from RDEB mice treated with daily injections of 0.1, 1.0 mg/kg Ang‐(1‐7), or PBS for 7 weeks. Quantifications after normalization to β‐tubulin for pSMAD‐2/3 (pSer 465/467 SMAD‐2/pSer 423/425 SMAD‐3), thrombospondin‐1 (Thbs‐1), fibronectin, tenascin‐C, and α‐smooth muscle actin (α‐SMA). Individual data points from individual mice, mean ± SEM, are shown. Data were analyzed by one‐way ANOVA with Dunnett’s correction. *P* values < 0.05 are considered significant. *N* = 4 organs from four different mice per condition.

Analyses of fibrosis progression in RDEB indicated a trajectory of tissue damage, inflammation, and fibroblast activation. Among the proteins that were commonly dysregulated in both back skin and paws, kininogen‐1 appeared unique by being increased in RDEB mice at both analyzed sites (in 4‐week‐old and 10‐week‐old RDEB paws and in back skin from 10‐week‐old RDEB mice) (Fig EV3, EV4, EV5A–C). Kininogen‐1 is primarily expressed by the liver; its abundance in intact skin is low but notably enhanced after injury (Schremmer‐Danninger *et al*, 2004; Merkulov *et al*, 2008). It is processed by proteases such plasma kallikrein and neutrophil‐derived proteinase 3 into biologically active peptides including the pro‐inflammatory, nonapeptide bradykinin (Kahn *et al*, 2009). Kininogen‐1 and its derived peptides are key components of the KKS which is inter‐connected with the RAS—counter‐regulation follows through joint employment of ACE and receptor heterodimerization (Su, 2014). The RAS is known as a principal regulator of inflammation and tissue repair (Su, 2014; Bernstein *et al*, 2018). The natural RAS heptapeptide Ang‐(1‐7) is anti‐inflammatory and tissue damage‐response weakening; it activates the fibrosis‐limiting axis of the RAS and acts as a bridge between the RAS and the KKS potentiating fibrosis‐limiting KKS signaling (Li *et al*, 1997; Sancho‐Bru *et al*, 2007; Su, 2014; Simões E Silva & Teixeira, 2016). Because Ang‐(1‐7) would engage two anti‐inflammatory axes dysregulated in injured RDEB skin (Fig EV3, EV4, EV5A–C and Nyström *et al*, 2015)—the KKS and RAS—it is an attractive candidate therapeutic and we tested its applicability for RDEB.

**Figure 4:**
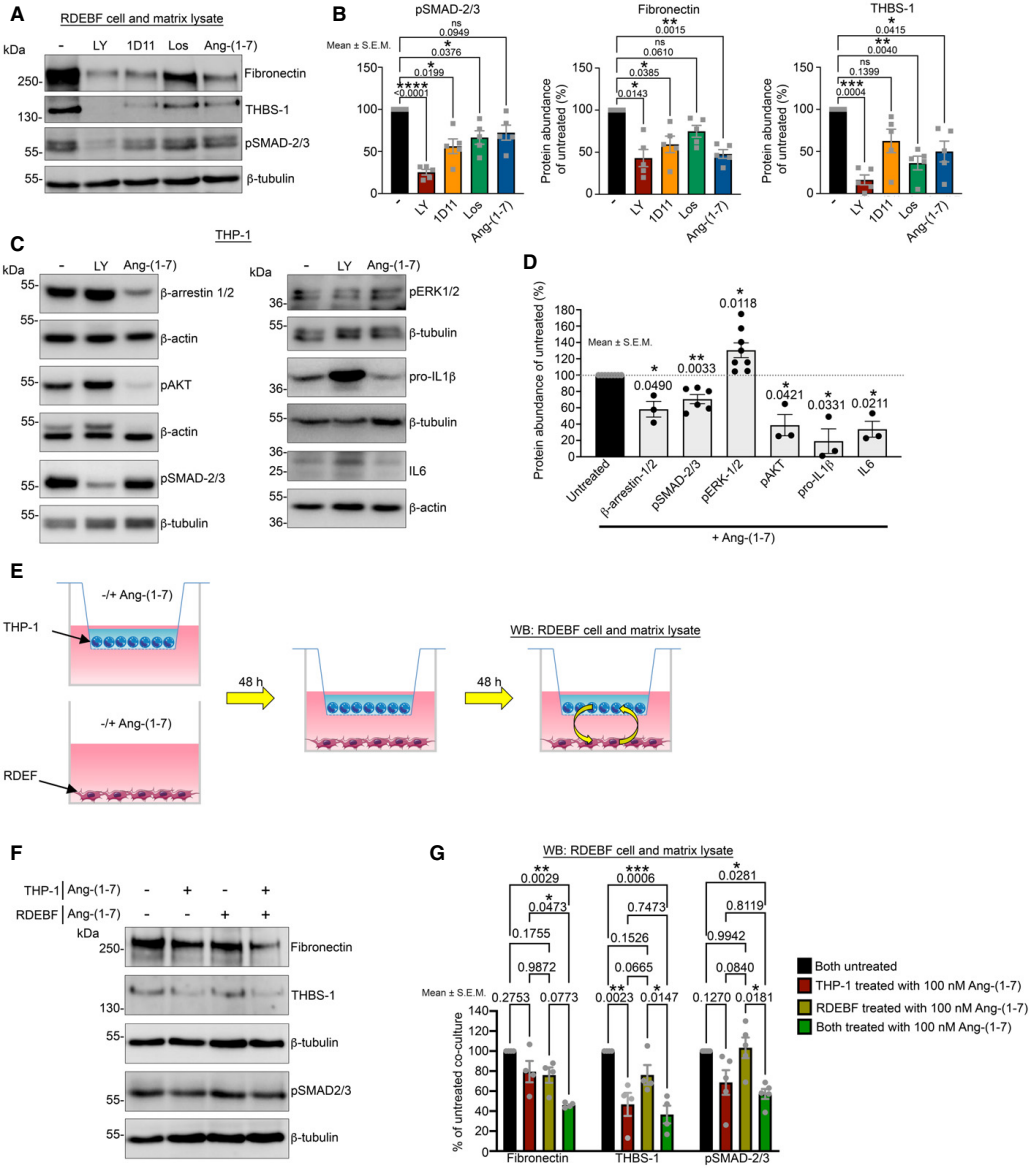
Ang‐(1‐7) exerts sustained, inflammatory cell‐mediated effects to deactivate fibroblasts Human dermal RDEBF treated daily with 10 μM LY2109761 (LY) (TGF‐β receptor I/II inhibitor), 10 μM 1D11 (neutralizing antibody to all TGF‐βs) 100 nM Ang‐(1‐7), or 10 μM losartan (AT1R antagonist). After five days of treatment cell and matrix lysates were extracted and analyzed by Western blotting for the indicated proteins, THBS‐1, thrombospondin‐1, β‐tubulin were used as loading control.Densitometric quantification of blots as in A. The abundance was normalized to β‐tubulin and then expressed as % of untreated (−). *P* values are shown; the data were analyzed with one‐way ANOVA with Dunnett’s correction (*n* = 5 biological replicates).Western blots of cell lysates from THP‐1 monocytes treated daily with 10 μM LY2109761 or 100 nM Ang‐(1‐7) for two days. The blots were probed with antibodies against the indicated proteins. β‐tubulin or β‐actin was used as loading control.Densitometric quantification of Western blots from THP‐1 monocytes treated daily with 10 μM LY2109761 or 100 nM Ang‐(1‐7) for 2 days. The abundance was normalized to β‐tubulin or β‐actin and then expressed as % of untreated (−). *P* values are shown; data were analyzed with one‐way ANOVA with Dunnett’s correction (*n* = 3–8 technical replicates).Schematic illustration of fibroblast‐THP‐1 monocyte co‐culture assay to assess the sustained effect of Ang‐(1‐7) and their principal cellular mediators.Western blots of fibroblast lysates from co‐cultures as described in E. The blots were probed with antibodies detecting fibronectin, thrombospondin‐1 (THBS‐1), and pSMAD‐2/3. β‐tubulin was used as loading control.Densitometric quantification of Western blots of fibroblast cell and matrix lysates as shown in F from multiple independent experiments, *n* = 4−5 using cells from 4 donors with RDEB. The abundance was normalized to β‐tubulin and then expressed as % of both untreated. The data were analyzed by one‐way ANOVA with Tukey’s correction. Data information: *P* values < 0.05 are considered significant and shown, ns = not significant. For B, D, and G individual data points, mean ± SEM, are shown. Source data are available online for this figure.

First, we treated primary human dermal RDEB fibroblasts (RDEBF) with Ang‐(1‐7) in gel contraction assays. Once‐daily addition of Ang‐(1‐7) for two days reduced gel contraction compared to vehicle treatment (Appendix Fig S5). Western blotting of RDEBF showed that Ang‐(1‐7) reduced canonical TGFβ signaling (pSMAD‐2/3) and fibrosis‐associated transitional ECM proteins thrombospondin‐1 and fibronectin—both known to be increased in RDEB (Nyström *et al*, 2015; Atanasova *et al*, 2019)—stronger than angiotensin II type 1 receptor (AT1R) targeting with losartan (Fig 4A and B). However, both compounds were less efficient than direct targeting of TGFβ signaling (TGFβ receptor I/II inhibitor LY2109761 and TGFβ‐ligand binding monoclonal antibody 1D11) (Fig 4A and B). These observations suggested that Ang‐(1‐7) evokes a modest fibroblast‐deactivating response directly in RDEBF.

To address the effect on inflammatory cells, human THP‐1 monocytic cells were exposed to Ang‐(1‐7). THP‐1 cells responded to Ang‐(1‐7) by a reduction in pSMAD‐2/3 and pAKT and a slight increase in pERK (Fig 4C and D). The putative Ang‐(1‐7) receptors MAS and AT1R are G protein‐coupled receptor (GPCR) and show strong association with β‐arrestin‐1 or β‐arrestin‐2 after internalization (Smith & Rajagopal, 2016; Cerniello *et al*, 2017). Depending on the signaling strength and features of the ligand, the GPCR with connected β‐arrestin‐1/2 can be targeted for lysosomal degradation or, inversely, synthesis of β‐arrestin‐1/2 can be promoted (Dale *et al*, 2004; Wang *et al*, 2017). Thus, β‐arrestins are essential influencers of GPCR signaling as they regulate receptor desensitization and internalization, and build signaling scaffolds (Smith & Rajagopal, 2016). Intriguingly, we found that Ang‐(1‐7) reduced β‐arrestin‐1/2 in THP‐1 cells (Fig 4B). Downstream, Ang‐(1‐7) treatment reduced production of pro‐inflammatory cytokines interleukin (IL)1β and IL6 (Fig 4C and D), but did not affect THP‐1 monocyte proliferation (Appendix Fig S6).

### Long‐lasting effects to Ang‐(1‐7) exposure in cells

The above *in vivo* analyses indicated that enhanced inflammatory cell–fibroblast interactions occur during progression of fibrosis in RDEB. This is in line with previous investigations by others and us that showed fibroblast–macrophage interactions to be instrumental for the maintenance of a fibrotic microenvironment (Pakshir & Hinz, 2018; Lodyga *et al*, 2019). To mimic fibroblast‐inflammatory cell communication following injury in a tractable *in vitro* system, THP‐1 cells were activated by collagen on cell‐impermeable inserts (Bhattacharya *et al*, 2018), which were placed in cell culture wells containing serum‐activated RDEBF (Fig 4E). Activation by collagen increased expression of pro‐inflammatory *IL1B* and *IL6* in THP‐1 cells (Appendix Fig S7). Given the relatively long‐lasting effect, we had observed on phosphorylation of signaling molecules in cells exposed to Ang‐(1‐7), we reasoned that the cellular and subsequent tissue response to Ang‐(1‐7) could be long‐lasting. To test this, we treated either RDEBF, or THP‐1 cells, or both once‐daily for two days with 100 nM Ang‐(1‐7) and then co‐cultured the cells for two days without Ang‐(1‐7) (Fig 4E). Subsequent Western blotting of the RDEBF fraction revealed that Ang‐(1‐7) priming of THP‐1 cells was sufficient to induce long‐lasting deactivation of fibrogenic response, as shown by reduction of pSMAD‐2/3 and thrombospondin‐1 (Fig 4F and G). However, for reduction of fibronectin, treatment of both THP‐1 and fibroblasts prior to co‐culture was most efficient (Fig 4F and G). In conclusion, in a fibro‐inflammatory setting, Ang‐(1‐7) stimulation of inflammatory cells and fibroblasts results in long‐lasting fibroblast deactivation. This is intriguing in light of the fact that the short half‐life (in humans ˜30 min after intravenous administration) of Ang‐(1‐7) has been viewed as an obstacle for therapeutic application (Iusuf *et al*, 2008).

### Daily low‐dose Ang‐(1‐7) efficiently halts progressive dermal fibrosis

Based on the promising *in vitro* results, we searched for suitable doses of Ang‐(1‐7) for *in vivo* testing in RDEB mice. The range was predicted based on data in the literature. Clinical trials had used doses between 0.1 and 1.0 mg Ang‐(1‐7)/kg body weight. We approximated the maximal dose in circulation from infusion of 0.1 mg Ang‐(1‐7)/kg and 1.0 mg Ang‐(1‐7)/kg injections to 1 nM and 30 nM Ang‐(1‐7), respectively (Petty *et al*, 2009; Nair & Jacob, 2016). These concentrations are significantly higher than the endogenous circulating Ang‐(1‐7) concentration of 40 pM (Petty *et al*, 2009). To cover this range, monocultures of RDEBF were treated for two days with daily additions of 0.01–1,000 nM Ang‐(1‐7). Intriguingly, using pSMAD‐2/3 as a marker for silencing of fibroblast pro‐fibrotic activity a bell‐shaped response was observed (Appendix Fig S8A). Furthermore, the fibroblast‐deactivating effect mediated by inflammatory cells exposed to Ang‐(1‐7) was also dose‐dependent (Appendix Fig S8B). The strongest pSMAD‐2/3‐reducing effect was mediated by THP‐1 cells exposed to sub‐nM Ang‐(1‐7) concentrations. The MAS receptor is widely considered as the major receptor for Ang(1‐7) but also the AT1R has been implicated in mediating its biological effects (Bernasconi & Nyström, 2018). Studies in RDEBF with losartan and A779 to inhibit AT1R and MAS, respectively, indicated that AT1R appeared important for the deactivating response observed at low Ang‐(1‐7) concentrations (Appendix Fig S9A and B).

**Figure 5:**
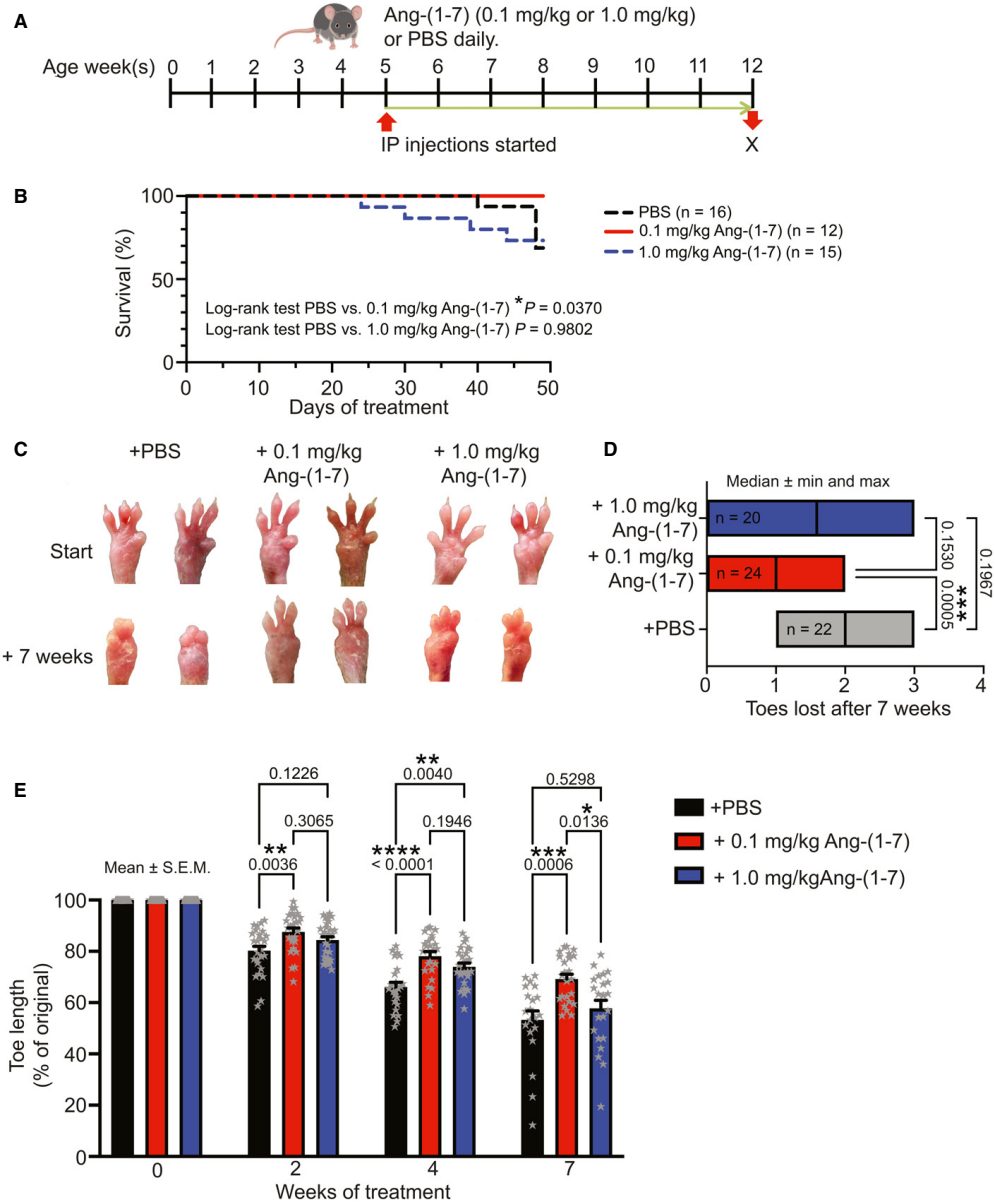
Daily administration of low‐dose Ang‐(1‐7) reduces severity of RDEB in mice Schematic illustration of the treatment regimen. From an average age of 5 weeks, RDEB mice were given daily injections of 0.1 or 1.0 mg/kg body weight Ang‐(1‐7) or an equal volume of PBS.Kaplan–Meier survival curve of mice treated as in A, *P* values as indicated, the data were tested with log‐rank test—PBS‐treated vs. 0.1 mg/kg Ang‐(1‐7)‐treated and PBS‐treated vs. 1.0 mg/kg Ang‐(1‐7)‐treated; *n* = 12–15 mice as indicated in the figure.Photographs of left and right forepaws of RDEB mice before and after 7 weeks of daily injections with 0.1, 1.0 mg/kg Ang‐(1‐7) or PBS.Plot of numbers of toes lost during the 7‐week observation period, data tested with Kruskal–Wallis test, *P* values indicated, *n* = 20–24 paws as indicated in the figure. Median and range are shown.Toe length measurement of forepaws (Nyström *et al*, 2015), expressed as the percentage of original length. Mice were treated as in A, values for treatment start (0) and 2, 4, and 7 weeks of treatment are shown. The data were analyzed by one‐way ANOVA with Tukey’s correction. *P* values < 0.05 are considered significant and shown; *n* = 20–24 paws. Individual data points, mean ± SEM, are shown.

Next, going *in vivo*, we tested two doses, 0.1 mg/kg Ang‐(1‐7) and 1.0 mg /kg Ang‐(1‐7), based on the differential dose responses observed *in vitro*. Because of the extended *in vitro* effects, once‐daily systemic administration of Ang‐(1‐7) was considered sufficient. To stratify the treatment groups, mice were enrolled when they displayed first signs of paw deformities (35 ± 9, 35 ± 8 days and 35 ± 7 days old at treatment start in the PBS, 0.1 mg/kg and 1.0 mg/kg groups, respectively) (Nyström *et al*, 2015). The mice received daily intraperitoneal injections of PBS or Ang‐(1‐7) and were followed for 7 weeks, which is the typical time needed for paw deformities to develop (Fig 5A).

Similar to *in vitro* observations, it appeared that a lower dose was more beneficial in reducing fibrogenic activities than a higher dose. Adult RDEB mice generally die prematurely from esophageal strictures. The lower dose of Ang‐(1‐7) conferred significant protection from early demise of RDEB mice, whereas no statistically significant difference in survival could be detected between higher dose and PBS treatment (Fig 5B). Furthermore, the lower dose of Ang‐(1‐7) evoked rapid, protracted and efficient attenuation of fibrosis progression, as indicated by loss of fewer toes (Fig 5C and D). Careful morphometric measurement of toe length (Nyström *et al*, 2015) disclosed sustained protective effects by the lower Ang‐(1‐7) dose and a mild transient protection by the higher Ang‐(1‐7) dose (Fig 5E).

### Slight modulation of tissue inflammation by Ang‐(1‐7)

**Figure 6:**
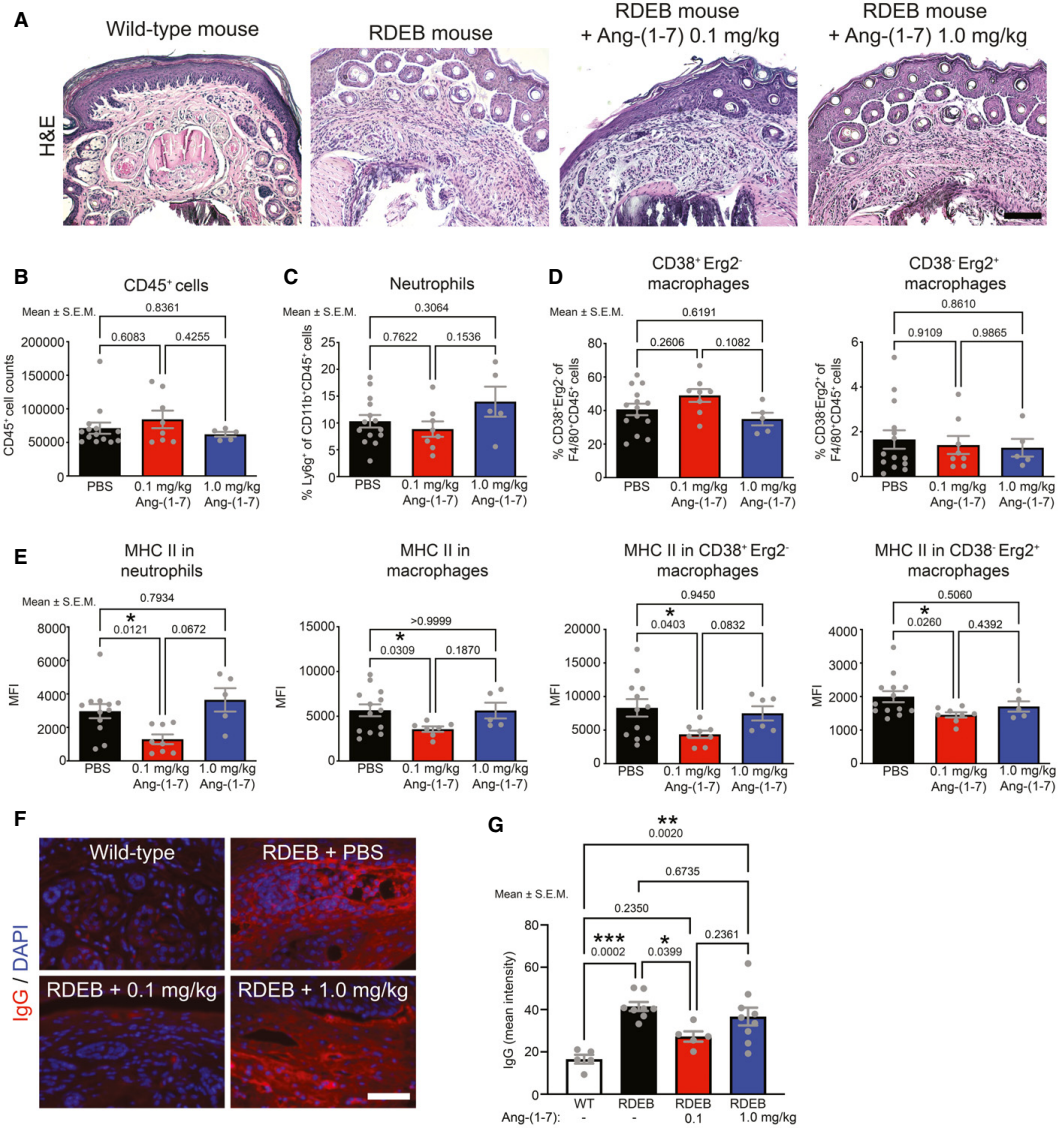
Modulation of dermal inflammation by Ang‐(1‐7) AHematoxylin and eosin (H&E) staining of toes from forepaws from WT and RDEB mice treated with daily injections of 0.1, 1.0 mg/kg Ang‐(1‐7) or PBS for seven weeks. Inflammatory cells are elevated in the toes from the PBS and the two Ang‐(1‐7)‐treated RDEB mice compared to the WT counterpart. Scale bar = 100 μm.B–DFlow cytometry analysis of the number of CD45‐positive cells (B), percentage of neutrophils (C) and of inflammatory macrophages (CD38^+^Egr2^−^) and tissue repair macrophages (CD38^−^Egr2^+^) (D) in forepaw skin of RDEB mice treated as in A.EThe mean fluorescence intensity (MFI) of MHC II staining on neutrophils and macrophages, inflammatory macrophage (CD38^+^Egr2^−^), and tissue repair macrophages (CD38^−^Egr2^+^) from mice as in B–D.FStaining for IgG (red) in forepaws, nuclei counterstained with DAPI.GQuantification of fluorescence staining intensity of samples as in F. Data information: Panels A and F, scale bar = 100 μm. For B–E and G, individual data points, mean ± SEM, are shown. The data were analyzed by one‐way ANOVA with Tukey’s correction, *P* values < 0.05 are considered significant and shown; *n* = 5–14 biological replicates. Source data are available online for this figure.

Since Ang‐(1‐7) reduced inflammatory activity *in vitro* (Fig 4), we postulated that Ang‐(1‐7) would alleviate tissue inflammation *in vivo*. Histological analyses of forepaws from mice treated for 7 weeks showed that the high‐dose Ang‐(1‐7) marginally reduced dermal inflammation as compared to PBS‐treated RDEB mice (Fig 6A). However, the low dose of Ang‐(1‐7) restored normal tissue histology to a certain extent, although not majorly affecting the dermal cell count. To test whether this improvement was due to altered presence of inflammatory cells, we performed flow cytometry analyses of forepaws. Consistent with histology, flow cytometry did not reveal major differences in the total number of inflammatory cells between Ang‐(1‐7)‐ and PBS‐treated RDEB forepaws (Fig 6B). Neither neutrophils (Fig 6C), nor inflammatory macrophages, nor tissue repair macrophages (Fig 6D) exhibited altered abundance in RDEB mouse forepaws.

Because Ang‐(1‐7) only moderately reduced inflammatory cells in tissue but substantially protected against fibrosis‐driven toe loss, we next assessed whether Ang‐(1‐7) controls inflammatory cell activation using expression of MHC II in neutrophils and macrophages as a proxy. Systemic delivery of 0.1 mg/kg Ang‐(1‐7), but not 1.0 mg/kg, caused significant decrease in MHC II on neutrophils and inflammatory and tissue repair macrophages in paw skin (Fig 6E). Subsequent analyses of blood suggested limited effect of the low dose of Ang‐(1‐7) on systemic inflammation (Appendix Fig S10A). This led us to investigate if the benefit of Ang‐(1‐7) depended on the level of tissue damage and toward that end analyzed two further tissues, the mildly affected back skin and the spleen. The low dose of Ang‐(1‐7) significantly reduced MHC II abundance on macrophages in back skin but not in spleen (Appendix Fig S10B and C). This alludes to specific effects of Ang‐(1‐7) in a destabilized tissue microenvironment. In accordance with lower antigen‐presenting and adaptive immune‐activating capabilities via reduction of MHC II, a concomitantly reduced abundance of dermal tissue‐bound IgG (Fig 6F and G) was noted in the paws from RDEB mice that had received 0.1 mg/kg Ang‐(1‐7).

### Low‐dose Ang‐(1‐7) reduces multi‐organ fibrosis in vivo

**Figure 7:**
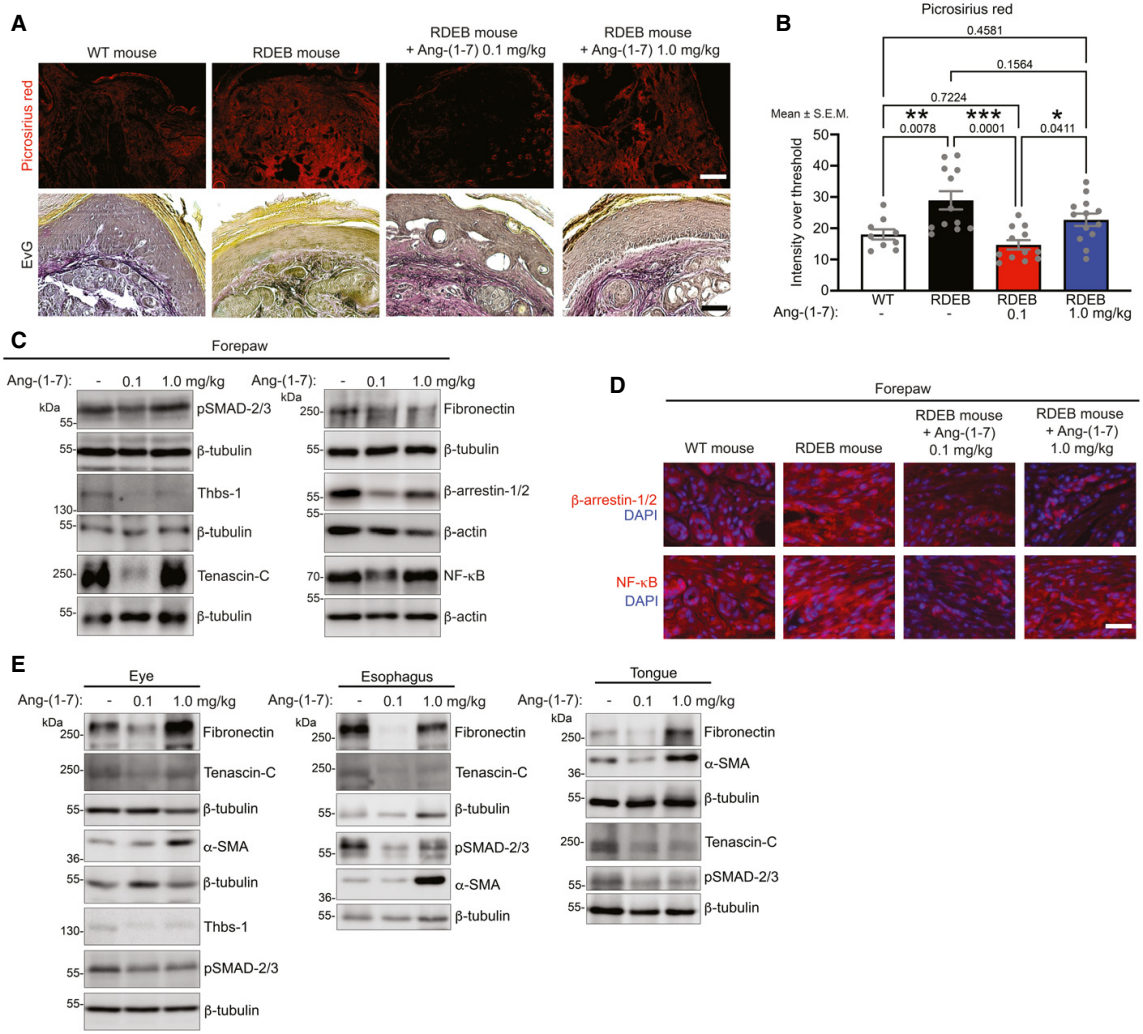
Dermal and systemic fibrosis in RDEB is effectively attenuated by low‐dose Ang‐(1‐7) Picrosirius red staining visualized under polarized light and Elastica van Gieson (EvG) staining) of forepaw toes from 12‐wk‐old WT or RDEB mice treated with daily injections of 0.1, 1.0 mg/kg Ang‐(1‐7), or PBS for 7 weeks. Note the reduced signal for picrosirius red in RDEB mice treated with 0.1 mg/kg Ang‐(1‐7) indicating less parallel alignment of and/or thinner collagen fibrils, and additionally the more organized and less disrupted appearance of elastic fibers (black in the EvG staining).Quantification of picrosirius red stained forepaws visualized as in A. A digital threshold was applied to the red channel before quantification. The data were analyzed by one‐way ANOVA with Tukey’s correction, *P* values < 0.05 are considered significant; *n* = 10–15. Individual data points, mean ± SEM, are shown.Western blotting for the indicated proteins on tissue lysates from forepaws from RDEB mice treated as in A. α‐SMA, α‐smooth muscle actin; Thbs‐1, thrombospondin‐1. Quantifications of multiple samples are shown in Fig EV4.Dermal β‐arrestin‐1/2 (red) and NFκB (red) staining in forepaws from WT and RDEB mice treated as in A. Nuclei counterstained with DAPI (blue).Western blot for the indicated proteins on tissue lysates from eyes, esophagus, or tongue from RDEB mice treated as in A. Quantifications of multiple samples are shown in Fig EV5. Data information: β‐actin or β‐tubulin was used as loading control for C and E. A, scale bar = 100 μm; D, scale bar = 20 μm. Source data are available online for this figure.

Fibrotic tissue remodeling was visualized by picrosirius red staining for organization of fibrillar collagens and by Elastica van Gieson staining for the organization of elastic fibers (Fig 7A and B). In accordance with the macroscopic observations, the low dose of Ang‐(1‐7) effectively reduced fibrotic remodeling, as seen by less pronounced, parallelly aligned or thickened collagen fibrils and by stronger staining of elastic fibrils (Fig 7A and B). Biochemical analyses of forepaws supported the macroscopic and histological observations. First, again the higher Ang‐(1‐7) dose rendered no discernable effects by histological (Fig 7A and B), biochemical, or proteomic analyses (Fig 7C, Appendix Fig S11, Tables EV1 and EV2). On the other hand, daily administration of 0.1 mg Ang‐(1‐7)/kg for seven weeks was associated with significant reduction in canonical TGFβ signaling, as evidenced by lower pSMAD‐2/3 levels, and reduced abundance of the transitional ECM proteins thrombospondin‐1, tenascin‐C, and fibronectin (Figs 7C and EV4). In alignment with *in vitro* data and reduced inflammatory potential, β‐arrestin‐1/2 and NF‐κB were significantly reduced in Ang‐(1‐7)‐treated forepaws compared to PBS‐treated forepaws (Figs 7C and EV5). This finding was validated by immunofluorescence staining, which revealed a decrease of β‐arrestin‐1/2 and NF‐κB in dermal cells in treated forepaws (Fig 7D).

Lastly, wider assessment of the fibrosis‐delaying effect of Ang‐(1‐7) was conducted by analyzing additional organs frequently affected by fibrosis in RDEB—eye, tongue, and esophagus. Also in these organs, the low dose of Ang‐(1‐7) reduced abundance of fibrosis‐associated proteins (Figs 7E and EV5), indicating that the fibrosis‐delaying effects of Ang‐(1‐7) were systemic.

In sum, the natural heptapeptide Ang‐(1‐7) in lower doses potently ameliorates disease severity and multi‐organ fibrosis associated with RDEB. It mediates these effects through dual targeting of fibroblasts and inflammatory cells resulting in altered cell–cell communication on the tissue level. This holds promise for therapeutically translatable symptom relief in RDEB.

## Discussion

Tissue destabilization reducing the threshold of damage resistance is a common early event in fibrotic diseases. However, the mechanistic and molecular progression of these events to established fibrosis is incompletely mapped. Detailed understanding of such mechanisms in time and space is important, since it will allow for the development of targeted treatment options for a broad spectrum of diseases. The current, incomplete understanding is to some extent a consequence of the models used to simulate fibrogenic responses in tissue, which are often based on chemical induction of extensive tissue damage and inflammation (Jun & Lau, 2018). Although such models have undoubtedly been useful for delineation of certain fibrotic pathomechanisms and treatment evaluation, they do not replicate the self‐sustaining progressive aspects of fibrosis which depend, at least in part, on subtle changes of the ECM (Herrera *et al*, 2018).

Here, we employed a clinically relevant genetic model predisposed to multi‐organ fibrosis—RDEB—to conduct comprehensive analyses of the natural progression of fibrosis. Importantly, in RDEB, conspicuous fibrosis developed without increased deposition of major structural ECM proteins. Rather, fibrosis in RDEB was driven by altered organization of the ECM through few and selected changes of minor components as identified by MS‐based proteomics. Due to the chosen peptide‐based, relative protein quantification, we cannot exclude that an altered extent of crosslinking of ECM proteins in the different genotypes affects our MS‐based quantification. However, as we observed the same trends by Western blot approaches, we are confident that our data reflect the *in vivo* situation. In pre‐ and post‐fibrotic skin, inflammation emerged as the most prominently altered process. Previous analyses of adult RDEB mice with advanced disease but partially differential disease severity had shown a positive correlation between presentation of fibrosis and the number of inflammatory cells in skin (Nyström *et al*, 2015).

There are clear links between inflammation and regulation of ECM organization in the context of scarring and fibrosis. For example, macrophage‐produced Relm‐α stimulates collagen crosslinking by inducing production of lysyl hydroxylase 2 in fibroblasts (Knipper *et al*, 2015). However, these actions commonly depend on tissue repair macrophages, which do not seem to play an important role in the fibrosis we describe. Instead, macrophages that contained molecular markers of inflammatory cells were ominous. With a progressively enhanced surface expression of MHC II, they showed similarities to pro‐fibrotic trained inflammatory macrophages (Jeljeli *et al*, 2019). Macrophages, via MHC II‐mediated antigen presentation, may promote the establishment of tissue tolerance. However, depending on the context this presentation may also induce an adaptive immune response through presentation of antigens from necrotic cells (Buxadé *et al*, 2018). Increased self‐antibody stimulation of innate immune cells in skin through Fcγ receptors has been associated with skewing macrophages from an inflammatory to a tissue repair phenotype (Andreu *et al*, 2010). In the present study, despite enrichment of tissue‐deposited antibodies we did not detect tendencies of macrophage phenotype switching at later stages, and a type 1 immune response seemed to predominate throughout the course of the disease. Thus, fibrosis in this context appears to be a pro‐inflammation‐driven fibrosis rather than a tissue repair‐driven fibrosis.

Since RDEB is a disease associated with the development of aggressive squamous cell carcinomas and immunotherapies are emerging as potential effective treatment option for those (Bardhan *et al*, 2020), it is important to avoid strong suppression of the immune system when targeting inflammation in RDEB. Toward this end, we selected Ang‐(1‐7) as a candidate to downmodulate tissue inflammation. Indeed, low‐dose Ang‐(1‐7) protractedly dampened fibrosis‐promoting communication between inflammatory cells and fibroblasts in a humanized *in vitro* model of RDEB. In RDEB mice, it attenuated progression of fibrosis in multiple organs through subtle targeting of tissue inflammation and fibroblast activity, thus leading to better‐organized dermal ECM. Functionally, this translated into retained dexterity of forepaws.

Low‐dose Ang‐(1‐7) injections resulted on the tissue level in changes of proteins that account for various aspects of the fibrosis‐delaying effects observed. The abundance of NF‐κB, which coordinates fibroblast‐driven tissue inflammation (Erez *et al*, 2010), was decreased in paws from Ang‐(1‐7)‐treated RDEB mice. This is in line with the subtly dampened inflammatory activity in skin. Both *in vitro* and *in vivo* analyses revealed that lower doses of Ang‐(1‐7) displayed stronger fibrosis‐limiting effects than higher doses. We did not further go into the mechanisms behind these dose‐dependent differences. However, previous studies on the decarboxylated form of Ang‐(1–7)‐Ala1‐Ang‐(1–7) have also shown a bell‐shaped dose response and alluded to dose‐dependent differential coupling of Gαi and Gαs proteins behind this response (Tetzner *et al*, 2018). Importantly, higher concentration of a ligand has been reported to lead to lower recruitment of β‐arrestin to GPCRs (Hübner *et al*, 2016). Interestingly, the low dose of Ang‐(1‐7) significantly reduced β‐arrestin‐1/‐2 in RDEB mouse skin. Deletion of β‐arrestin‐1/‐2 has been reported to protect against fibrosis without reducing the number of inflammatory cells (Lovgren *et al*, 2011). Thus, these data align well with our observation that Ang‐(1‐7) does not mediate clear reduction of the activities in monocultured dermal fibroblasts or inflammatory cells. Collectively, by targeting multiple pathways in fibroblasts and inflammatory cells Ang‐(1‐7) efficiently reduces the pro‐fibrotic aptitude of the tissue microenvironment in injury‐induced fibrosis.

Taken together, the global and molecular delineation of naturally progressing injury‐evoked fibrosis and its inhibition via Ang‐(1‐7) in a preclinical setting represent a substantial step forward to effective therapies for fibrotic diseases. Specifically at this stage, our work supports the initiation of clinical testing of Ang‐(1‐7) for RDEB. Importantly, by revealing a fibrosis independent of the increase in major structural proteins and driven by inflammatory immunity, our data underscore mechanistic diversity among fibrotic diseases which is already an active area of research (Schaefer, 2018 and references therein). This knowledge will have direct implications for the design of therapies to combat fibrosis.

## Materials and Methods

### Studies using mouse and human material

Studies using mice were approved by the regional review board (Regierungspräsidium Freiburg, Freiburg, Germany; approval numbers 35/9185.81/G15‐140, G14‐93, G10‐118). For *in vitro* studies, primary dermal fibroblasts from six human control donors and six human donors with RDEB were used. The latter were selected based on absence of collagen VII abundance as detected by Western blotting (Küttner *et al*, 2013). Human skin sections for immunofluorescence analysis were derived from surplus material biopsies taken for diagnostic purposes, from healthy young (3–8 years old) and adult human donors (> 25 years old) and from human donors with RDEB with different stages of fibrosis—early (0–3 years old), mild (6–10 years old), and advanced (> 15 years old). The RDEB patients were selected based on total collagen VII deficiency by immunofluorescence staining. The patients gave informed consent for use of the materials for research, and the study was approved by the ethics committee of the University of Freiburg (approval no. 318/18) and was performed in agreement with the principles of the Declaration of Helsinki and the Department of Health and Human Services Belmont Report.

The collagen VII hypomorphic mice—RDEB mice (Nyström *et al*, 2015), were kept on mixed C57BL/6 129sv background. After weaning, RDEB mice were followed weekly for appearance of forepaws deformities. At the first sign of toe loss, the mice were randomized into three treatment groups: 1, daily intraperitoneal injections with PBS (16 mice, 11 females and 5 males); 2, 0.1 mg Ang‐(1‐7) /kg body weight (12 mice, 6 females and 6 males); 3, 1 mg Ang‐(1‐7)/kg body (15 mice, 10 females and 5 males). Ang‐(1‐7) in the form of TXA127 was provided by Constant Therapeutics, Boston, Massachusetts, United States. All mice were daily monitored and weighed and weekly photographed. At treatment start the mice were on average 35 ± 9 days old in the PBS group, 35 ± 8 days in the 0.1 mg/kg Ang‐(1‐7)‐treated group, and 35 ± 7 days old in the 1 mg/kg Ang‐(1‐7)‐treated group. Morphometric quantification of mutilating deformities was performed as previously described on photographs, using ImageJ (NIH, Bethesda, MD, USA; Nyström *et al*, 2015; Cianfarani *et al*, 2019). The study was designed to comply with the ARRIVA guidelines (Kilkenny *et al*, 2010). The sample size was calculated on the variance from previous studies (Nyström *et al*, 2015) by performing a log‐rank test with a power of 80% and for a *P* value < 5%. Data collection was stopped once the sample size had reached the estimated number; no outliers were removed.

### Stiffness measurements

A MACH‐1™ Mechanical Testing System (Biomomentum) with a flattened needle was used for indentation measurement of stiffness of palm skin. 10‐week‐old WT and RDEB mice (three per group) were used and per paw six areas were measured.

### Western blotting

Cell and matrix lysates were lyzed in radioimmunoprecipitation assay buffer. Tissue was frozen on dry‐ice, crushed, and boiled under harsh vortexing for 20 min in 4× blue sample buffer containing 4% SDS and 8 M Urea, boiled 20 min. Western blotting was performed under standard conditions as previously described (Nyström *et al*, 2015) and images captured with Amersham Imager 600 (GE healthcare). Image J (NIH) was used for densitometric quantification.

### Reagents and antibodies

The following antibodies were used: primary antibodies: rabbit anti‐pSMAD‐3 (S423/425) (ab52903) (for Western blotting (WB) 1:1,000), rabbit anti‐fibronectin (ab2413) (for WB 1:1,000), mouse anti‐IL6 (ab9324) (for WB 1:500), rabbit anti‐IL1‐β (ab9722) (for WB 1:500), rabbit anti‐kininogen‐1 (ab175386) (for WB 1:1,000), rabbit anti‐periostin (ab14041) (for immunofluorescence (IF): 1:50), rabbit anti‐β‐tubulin (ab6046) (for WB 1:1,000) all from Abcam; rabbit anti‐pSMAD‐2 (Ser 465/467) (138D4) (for WB 1:1,000), rabbit anti‐NF‐kB p65 (D14E12) (for IF: 1:50; for WB 1:1,000), rabbit anti‐β‐arrestin‐1/2 (D24H9) (for IF: 1:50; for WB 1:1,000), rabbit anti‐pAkt (Ser 473) (D9E) (for WB 1:1,000) from Cell Signaling; rat anti‐mouse tenascin‐C clone 578 from R&D Systems) (for IF: 1:100; for WB 1:1,000); CD68 mouse monoclonal Antibody (KP1) (for IF: 1:1,000), CD3 ɛ mouse monoclonal Antibody (UCH‐T1) (for IF: 1:200); mouse anti‐α‐smooth muscle actin (clone 1A4) (for WB 1:2,000) and mouse anti‐activated MAP kinase (diphosphorylated Erk‐1/2) (for WB 1:5,000) from Sigma; mouse anti‐thrombospondin‐1 clone A6.1 (for WB 1:1,000) and rat anti‐mouse syndecan‐1 (clone DL‐101) (for IF 1:200) from Thermo Fisher Scientific, rabbit anti‐collagen I (Origene) (for WB 1:500); secondary antibodies: HRP‐conjugated goat anti‐rabbit (KPL, cat. Nr. 474‐1506) (1:10,000), HRP‐conjugated mouse anti‐β‐actin (C4) (sc‐47778) (Santa Cruz Biotechnology) (1:1,000), HRP‐conjugated goat anti‐mouse (Calbiochem, cat. Nr. 401253‐2ML) (1:5,000), HRP‐conjugated goat anti‐rat (Jackson Immuno Research, cat. Nr. 112‐035‐167) (1:5,000), Alexa Fluor 594 goat anti‐rat IgG (Invitrogen, ref. A11007) (1:1,000) and Alexa Fluor 488 goat anti‐rat IgG (Invitrogen, ref. A11006) (1:1,000).

For flow cytometry analyses: anti‐CD45 APC/Cy7 clone 30‐F11 (BioLegend, ref. 103116) (1:50), anti‐CD11b V450 clone M1/70 (BDHorizon, ref. 560455) (1:50), anti‐CD11b FITC clone M1/70 (BD Biosciences ref. 557396) (1:50), anti‐F4/80 PerCP/Cy5.5 clone BM8 (BioLegend, ref. 123128) (1:50), anti‐mouse Ly6G Pe/Cy7 clone 1A8 (BioLegend, ref. 127618) (1:50), anti‐CD38 PE clone 90 (Invitrogen, ref. 12‐0381‐82) (1:50), anti‐Ly6C PeLy7 clone 1 HK1.4 (BioLegend ref. 128017) (1:50), anti‐CD62L biotinylated clone MEL14 (BD Biosciences ref. 104403) (1:50) stained together with APC‐streptavidin (1:200), anti‐MHCII eFluor 450 clone AF6‐120.1 (Thermo Fischer Scientific ref. 48‐5320‐82) (1:50), anti‐Egr2 APC (eBioscience, ref. 17‐6691‐82) (1:50), anti‐NK 1.1 APC clone PK136 (BioLegend) (1:50), anti‐CD3ɛ PE clone eBio500A2 (eBioscience) (1:100), anti‐CD8 PeCP clone 53‐7.6 (BioLegend) (1:200), anti‐CD4 Brilliant Violet 421 clone GK 1.5 (BioLegend) (1:200).

### Histological and immunofluorescence analyses

Cryo‐sectioned tissue was fixed in ice‐cold acetone or 4% paraformaldehyde, and paraffin sections were subjected to antigen‐mediated retrieval using 0.05% pronase, or heat and 10 mM sodium citrate buffer before blocking and staining with primary and secondary antibodies.

Hematoxylin and eosin and Elastica von Gieson staining followed standard protocols. Picrosirius red staining was performed as previously described (Cianfarani *et al*, 2019) and visualized under polarized light. ImageJ was used for quantification of staining intensity after a digital threshold had been applied (Cianfarani *et al*, 2019).

An Axioplan2 fluorescence microscope (Zeiss) and Axiocam camera were used for the analyses and image capture. For all parallel analyses, images were captured using identical settings and exposure time.

### Flow cytometry analysis

Tissue was carefully dissected and cut into small pieces transferred to tubes. Liberase™ (Roche, ref. 05‐401‐127‐001) was added, and the tubes were incubated at 37°C under agitation for 1 h. The lysates were passed through 40 μm nylon meshes. After centrifugation, supernatants were removed by aspiration, pellets resuspended in 100 μl of fluorescence‐activated cell sorting (FACS) buffer (DPBS 3% fetal bovine serum (FBS)), and the samples were transferred to FACS tubes. Blocking was performed using FC Block solution (clone 2.4G2 made by the Max Planck Institute, Freiburg, Germany) followed by staining with primary antibodies coupled to fluorophores. After washing, intracellular staining was performed using the BD intracellular staining kit (Catalog No. 554714) after manufacturer’s instructions.

Blood samples (about 100 μl) were collected from the left ventricle of the heart immediately after sacrifice. Erythrocyte lysis was performed using ACK lysis buffer. After incubating 2 min, the lysis was stopped by adding PBS, samples were centrifuged, pellets resuspended in FACS buffer and transferred to FACS tubes. Spleen samples were passed through 100 μm cell strainers and then prepared as blood samples. Blocking and staining steps were carried out as described above. Analyses were performed with BD FACSCantoTM II cytometer and FlowJo software.

### MS sample preparation

Skin specimens or whole forepaws were obtained from three age‐matched RDEB mice and three WT mice per time point. For Ang‐(1‐7) treatment studies forepaws of three age‐matched WT, RDEB mice treated with PBS and three RDEB mice treated with 1.0 mg Ang‐(1‐7) /kg were collected. After being sacrificed, the back skin was shaved and skin and paws snap‐frozen. Tissue was frozen on dry‐ice, crushed, and tissue homogenate boiled in an SDS–PAGE loading buffer containing 4% SDS with 100 mM Tris pH 7.6, 1 mM DTT, and 4 mM Pefabloc (Sigma) under harsh vortexing for 20 min. Proteins were separated by 4–12% gradient SDS–PAGE. The gel lanes were cut into 10 equal slices. Proteins were in‐gel digested with trypsin, and the resulting peptide mixtures were processed on STAGE tips and analyzed by LC‐MS/MS (Rappsilber *et al*, 2007).

### Mass spectrometry

Mass spectrometry measurements were performed on an LTQ Orbitrap XL mass spectrometer coupled to an Agilent 1200 nanoflow–HPLC. HPLC–column tips (fused silica) with 75 µm inner diameter were self‐packed with Reprosil–Pur 120 ODS–3 to a length of 20 cm (Gruhler *et al*, 2005). Samples were applied directly onto the column without a pre‐column. A gradient of A (0.5% acetic acid in water) and B (0.5% acetic acid in 80% acetonitrile in water) with increasing organic proportion was used for peptide separation (loading of sample with 2% B; separation ramp: from 10–30% B within 80 min). The flow rate was 250 nL/min and for sample application 500 nl/min. The mass spectrometer was operated in the data‐dependent mode and switched automatically between MS (max. of 1 × 10^6^ ions) and MS/MS. Each MS scan was followed by a maximum of five MS/MS scans in the linear ion trap using normalized collision energy of 35% and a target value of 5,000. Parent ions with a charge state from z = 1 and unassigned charge states were excluded for fragmentation. The mass range for MS was m/z = 370–2,000. The resolution was set to 60,000. MS parameters were as follows: spray voltage 2.3 kV; no sheath and auxiliary gas flow; ion–transfer tube temperature 125°C. Identification and quantification of proteins were performed with MaxQuant software version 1.4.1.2 as previously described (Nyström *et al*, 2015).

### Cluster analysis by fuzzy C‐means‐clustering

To obtain a list of proteins significantly altered in age‐matched WT and RDEB mice or RDEB mice +/− Ang‐(1‐7) treatment, the data were processed using limma‐voom (Smyth *et al*, 2005) with Galaxy. Label‐free protein abundance values based on extracted ion currents of peptides (LFQ) were analyzed using a limma test applying sample weights and Benjamini and Hochberg p‐value adjustment with a threshold of 0.05. Ratios of average LFQ values were log2‐transformed and z‐normalized with Perseus (Tyanova *et al*, 2016) and clustered with G‐Prox (Rigbolt *et al*, 2011). Fuzzy C‐Means‐Clustering was obtained via G‐Prox choosing six clusters and Fuzzification of 2.

### For principle component analysis and heatmap

Label‐free protein abundance values based on extracted ion currents of peptides were log2‐transformed and z‐normalized. Samples were hierarchically clustered, and protein abundances were k‐means‐clustered. To address the biological implications of the proteins in each cluster, gene ontology (GO) terms were retrieved using STRING (www.string‐db.org). Significantly enriched GO terms in each cluster were determined using Benjamini–Hochberg‐adjusted *P* values (< 0.05).

### Cell culture

The cell culture facility was routinely tested for mycoplasma contamination, and no mycoplasma contamination was detected during the period this work was performed. Human THP‐1 cells (TIB‐202) were purchased from ATCC, which have authenticated the cell line. Ang‐(1‐7) was dissolved in DPBS and added every day at 100 nM in accordance with previous publications (Acuña *et al*, 2014) to the growth medium for the indicated time. To determine a dose‐dependent effect, concentrations of 0.01 nM, 0.5 nM, 10 nM, 100 nM, and 1 μM Ang‐(1‐7) were used. For analyses of receptors, involved cells were pre‐incubated with losartan (10 μM) or A779 (1 μM) 1 h before adding Ang‐(1‐7).

Primary dermal human fibroblasts were isolated and controlled as previously described (Kruppa *et al*, 2021). For co‐culture experiment, 200,000 human RDEBF were seeded per well in 6‐well plates. Ang‐(1‐7) was added every 24 h to the medium for 2 days. THP‐1 monocytes were seeded on 0.4 μm‐pore rat tail collagen I‐coated (100 μg/ml) cell culture inserts (ThinCertTM, Greiner) and treated every 24 h for 2 days with Ang‐(1‐7). After two days, the inserts with THP‐1 cells were placed on top of RDEBF in 6‐well plates. THP‐1 cells and RDEBF were co‐cultured for 48 h in RPMI medium (Gibco) without addition of Ang‐(1‐7).

Murine bone marrow‐derived macrophages were prepared from flushed femoral bone marrow of newborn, 4‐wk‐old, and 10‐wk‐old WT and RDEB mice in accordance with previously published protocols (Weischenfeldt & Porse, 2008).

### Collagen‐lattice contraction assay

Collagen‐lattice contraction assays were performed in gels containing a final concentration of 0.4 mg/ml rat tail collagen I (Corning) and 0.1% FBS as previously described (Nyström *et al*, 2015) and quantified using ImageJ. Ang‐(1‐7) at a concentration of 1 nM or an equal volume PBS was added daily.

### RT–PCR

RNA was isolated using RNeasy Plus Mini Kit (QIAGEN), and concentration was measured a NanoDrop spectrophotometer. 500 ng was retrotranscribed to cDNA with First‐Strand cDNA Synthesis Kit (Thermo Fisher Scientific). qPCR analyses were performed using SYBR green (Bio‐Rad) and a CFX96 Real‐Time system (Bio‐Rad).

The primers used were as follows:

*Pdcd1*F TGCCCTAGTGGGTATCCCTG; *Pdcd1*R AAGGCTCCTCCTTCAGAGTG; *Cd27*F ACTCGGTACAAGCAGTTGGG; *Cd27*R ACAAAGAATGTACCTGGCTCACA; *Eomes*F GGACAACTACGATTCATCCCA; *Eomes*R GGCTTGAGGCAAAGTGTTGAC; *Tbet*F ATGCCAGGGAACCGCTTAT; *Tbet*R ATTGTTGGAAGCCCCCTTGT; *Gata3*F GCGCCGTCTTGATAGTTTCAG; *Gata3*R CTTCCGATCACCTGAGTAGCA; *Rorc*F AGAAGACCCACACCTCACAA; *Rorc*R GTGCAGGAGTAGGCCACATT; *Tlr4*F TGGTTGCAGAAAATGCCAGG; *Tlr4*R TAGGAACTACCTCTATGCAGGGAT; *Gapdh*F TTGATGGCAACAATCTCCAC; *Gapdh*R CGTCCCGTAGACAAAATGGT. *IL1B*F AGCTACGAATCTCCGACCAC; *IL1B*R CGTTATCCCATGTGTCGAAGAA; *IL6*F ACTCACCTCTTCAGAACGAATTG; *IL6*R CCATCTTTGGAAGGTTCAGGTTG; *GAPDH*F CCCATCACCATCTTCCAG; *GAPDH*R ATGACCTTGCCCACAGCC.

### Statistical analysis

The GraphPad Prism 9 software was used for statistical analysis. The datasets were tested for normality and equal variance using Shapiro–Wilk and F tests. Statistical analyses were performed using unpaired one‐way ANOVA with Tukey’s or Dunnett’s correction, Kruskal–Wallis test, and paired or unpaired two‐tailed Student’s t‐test as indicated. For all analyses, *P* < 0.05 was considered statistically significant.

## Author contributions

Conceptualization: AN, LBT, JD, BH; experimentation: RB, KT, ER‐F, CG, TK, MM, PN, SK, MS, A‐CR‐M, SFM; data interpretation: RB, KT, ER‐F, TK, MM, PN, SK, MS, A‐CR‐M, SFM, DK, SFM, BH, LBT, JD, AN; providing key materials: DK, SFM; funding acquisition: DK, BH, LBT, JD, AN; supervision: SFM, BH, LBT, JD, AN; manuscript writing: AN, RB, LBT, JD; Manuscript editing: RB, KT, ER‐F, TK, MM, PN, SK, MS, A‐CR‐M, DK, SFM, BH, LBT, JD, AN.

## Conflict of interest

The authors declare that they have no conflict of interest.

## Supporting information



Appendix

Expanded View Figures PDF

Table EV1

Table EV2

Dataset EV1

Dataset EV2

Dataset EV3

Dataset EV4

Source Data for Figure 2

Source Data for Figure 3

Source Data for Figure 4

Source Data for Figure 6

Source Data for Figure 7

## Data Availability

The MS proteomics data have been deposited to ProteomeXchange Consortium via the PRIDE partner repository with the dataset identifiers PXD019957 and PXD019929 and can be reached at https://www.ebi.ac.uk/pride/archive/projects/PXD019957 and https://www.ebi.ac.uk/pride/archive/projects/PXD019929, respectively.
