# Supplementary material for: Pro‐inflammatory immunity supports fibrosis advancement in epidermolysis bullosa: intervention with Ang‐(1‐7)
Source: EMBO Mol Med. 2021 Aug 30;13(10):e14392. doi: 10.15252/emmm.202114392 (PMC8495454; doi:10.15252/emmm.202114392)
Supplement: Supplementary file 1 — Appendix [file EMMM-13-e14392-s008.pdf]

Appendix Bernasconi et al.,

Appendix Table S1. List of mass spectrometry-based quantifications of protein abundances of age-matched WT and PBS-treated or 1.0 mg/kg Ang-(1-7)-treated RDEB mice.

Appendix Table S2. LIMMA statics of data from Appendix Table S1 of PBS-treated vs. 1.0 mg/kg Ang-(1-7)-treated RDEB mice.

Appendx Fig S1. Principal component analyses (PCA) illustrate different timing of fibrosis progression of mechanically challenged forepaw skin compared to protected back skin in RDEB mice.

Appendix Fig S2. *Tlr4* expression is increased in bone marrow-derived macrophages from mice with advanced RDEB.

Appendix Fig S3. Tissue-bound IgG is increased with progression of dermal fibrosis in RDEB.

Appendix Fig S4. Indications of progressively increased inflammatory immunity in RDEB forepaws.

Appendix Fig S5. Ang-(1-7) reduces the activity of human dermal RDEBFs.

Appendix Fig S6. Ang-(1-7) does not affect proliferation of THP-1 monocytes.

Appendix Fig S7. Collagen I-coating evokes expression of pro-inflammatory cytokines in THP-1 cells.

Appendix Fig S8. Ang-(1-7) evokes a bell-shaped concentration-dependent response.

Appendix Fig S9. AT1R mediates the response to low Ang-(1-7) doses in fibroblasts.

Appendix Fig S10. Ang-(1-7) subtly affects inflammation in back skin in RDEB mice.

Appendix Fig S11. High-dose of Ang-(1-7) has no global molecularly discernable protective effect on the dermal proteome of RDEB mice.

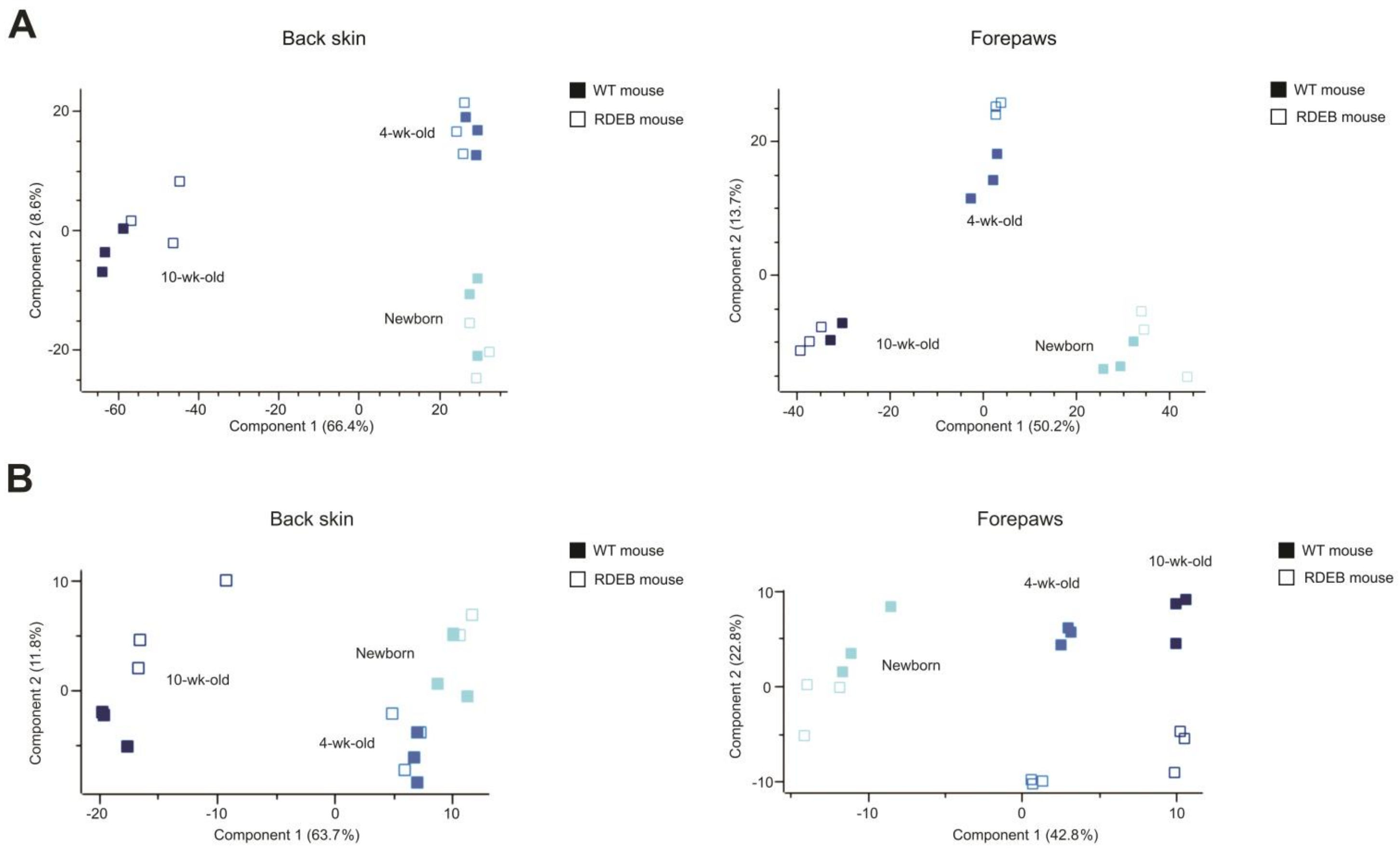

Appendix Fig S1. Principal component analyses (PCA) illustrate different timing of fibrosis progression of mechanically challenged forepaw skin compared to protected back skin in RDEB mice. (**A**) PCA was performed on all quantifiable proteins detected by MS-based analyses of back skin and forepaws from wild type (WT) and RDEB mice of the indicated ages. Left, the plot shows PCA for back skin. Right, the plot shows PCA for forepaws. Note the clear segregation of different ages as well as a closer but distinct segregation in forepaws of WT and RDEB mice starting from week 4. In back skin partial segregation between genotypes is observed in samples from 10-wk-old mice. (**B**) PCA was performed on proteins that were significantly changed in abundance between the different genotypes in at least one age group. Left, the plot shows PCA of significantly changed proteins in back skin. Right, the plot shows PCA for significantly changed proteins in forepaws. Segregation between RDEB and WT is for forepaws evident after 4 weeks, whereas in back skin this occurs later after 10 weeks. 3 WT and 3 RDEB samples were analyzed per time-point; in the plot of all quantifiable proteins of forepaws two data points of 10-wk-old samples overlap and cannot be discriminated.

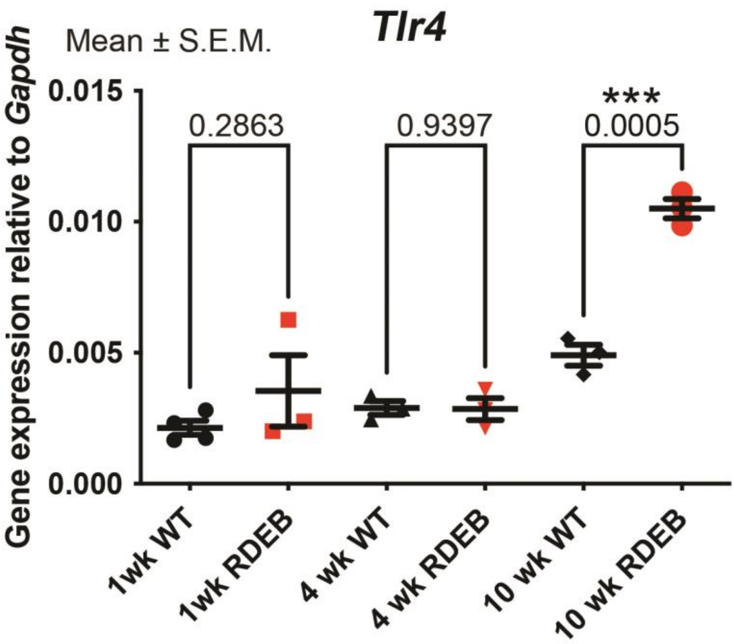

Appendix Fig S2. *Tlr4* expression is increased in bone marrow-derived macrophages from mice with advanced RDEB. Gene expression analyses of mRNA extracted from bone marrow-derived macrophages isolated from mice of indicated genotype and gene expression was normalized to *Gapdh*. Individual values, mean ± S.E.M are shown. The data for each genotype for age group were compared and tested with unpaired t-test. *P* values < 0.05 are considered significant; n = 3-4 mice.

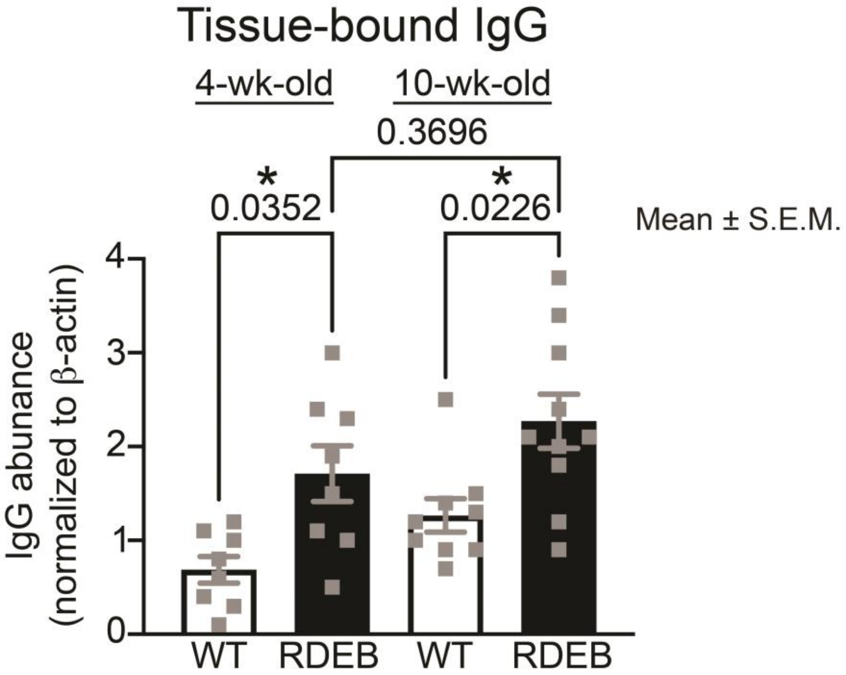

Appendix Fig S3. Tissue-bound IgG is increased with progression of dermal fibrosis in RDEB. IgG levels in forepaw lysates from mice of indicated ages and genotypes were analyzed by western blotting. The levels were normalized to β-actin. Individual data points, mean ± S.E.M are shown. The data were tested by one-way ANOVA with Tukey's correction. *P* values < 0.05 are considered significant. N = 7-10 mice per genotype and age.

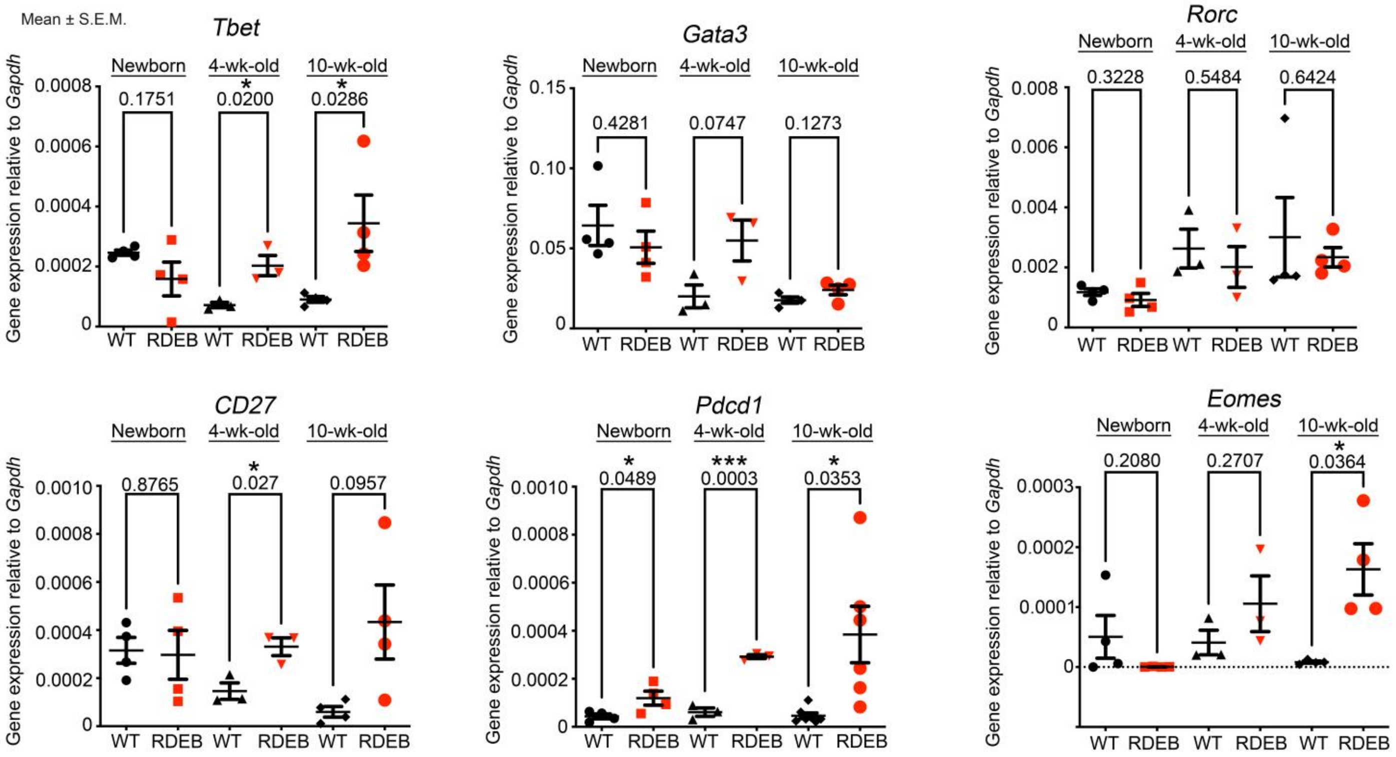

Appendix Fig S4. Indications of progressively increased pro-inflammatory immunity in RDEB forepaws. Gene expression analyses of mRNA extracted from forepaws of mice of indicated genotype and ages for *Tbet*, *Gata3*, *Rorc*, *Cd27*, *Pdcd1* and *Eomes* gene expression normalized to *Gapdh*. Individual data points, mean ± S.EM. are shown. The data for each genotype for age group were compared and tested with unpaired t-test. *P* values < 0.05 are considered significant; n = 3-6 mice.

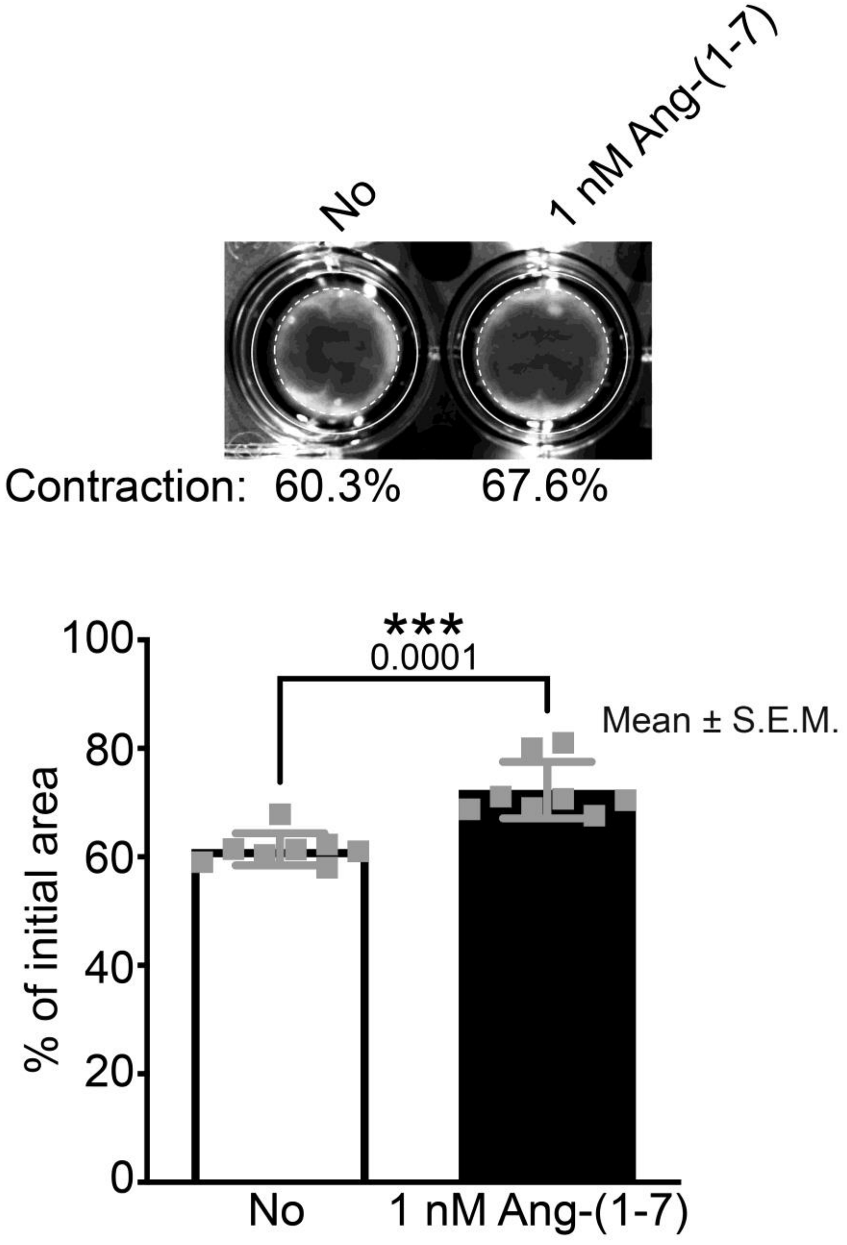

Appendix Fig S5. Ang-(1-7) reduces the activity of human dermal RDEBF. Collagen lattice-contraction assays are shown of free-floating collagen lattices populated with RDEBF. The lattices were allowed to contract for 24 h in the absence or presence of 1 nM Ang-(1-7). The quantification shows percentage area of original area of the lattices, written below the figure and shown in the bar graph. Individual data points, mean ± S.E.M are shown. Data were tested with unpaired t-test; P = 0.0001. N = 8 experiments using RDEBF from four donors.

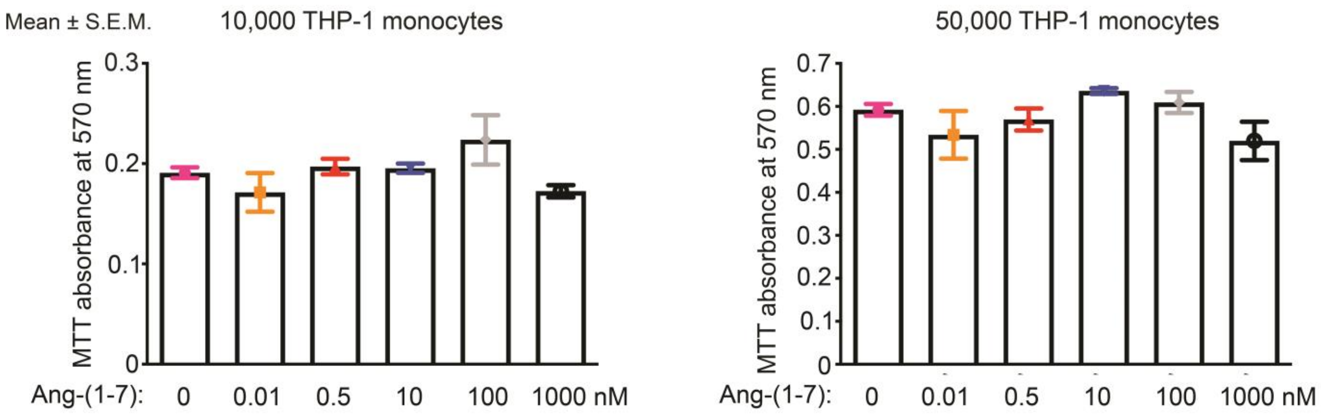

Appendix Fig S6. Ang-(1-7) does not affect proliferation of THP-1 monocytes. 10,000 or 50,000 THP-1 monocytes were seeded and treated with the indicated Ang-(1-7) concentrations daily for 48 h. Cell numbers were measured by MTT assay. Values represent mean ± S.E.M, n = 3 replicates. Data were statistically evaluated by one-way ANOVA with Tukey's correction.

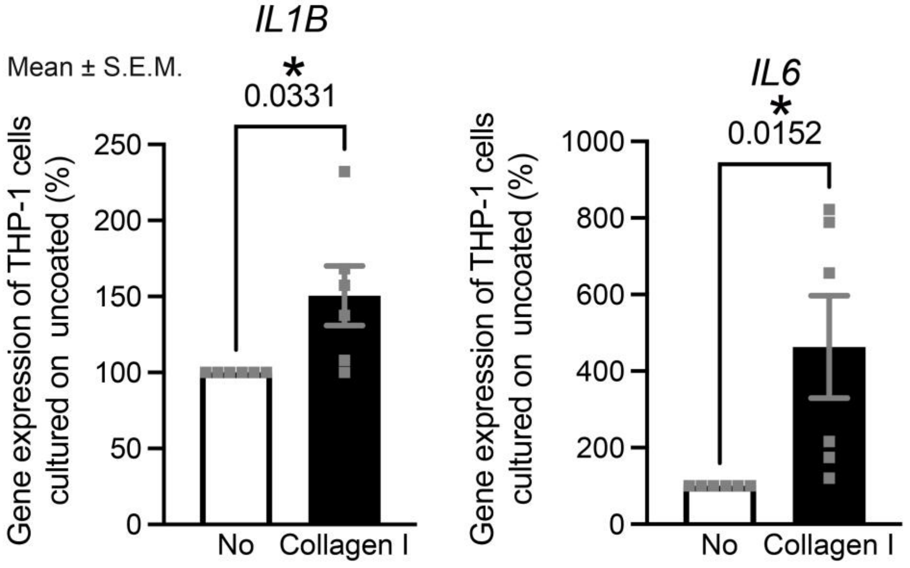

Appendix Fig S7. Collagen I-coating evokes expression of pro-inflammatory cytokines in THP-1 cells. THP-1 monocytic cells were seeded on uncoated 0.4 µm-pore cell culture inserts or 0.4 µm-pore cell culture inserts coated with rat tail collagen I (100 µg/ml) and placed in 12-well-plates with RPMI medium with 10% FCS. After 2 days, RNA was isolated from THP-1 cells, reverse transcribed to cDNA and analyzed for *IL1B* and *IL6* expression. Their expression was normalized to *GAPDH* and expressed as the percentage of expression on non-coated insert (no) inserts. Individual data points from independent experiments (n = 6), mean ± S.E.M are shown. The data were analyzed by paired t-test and *P* values are shown

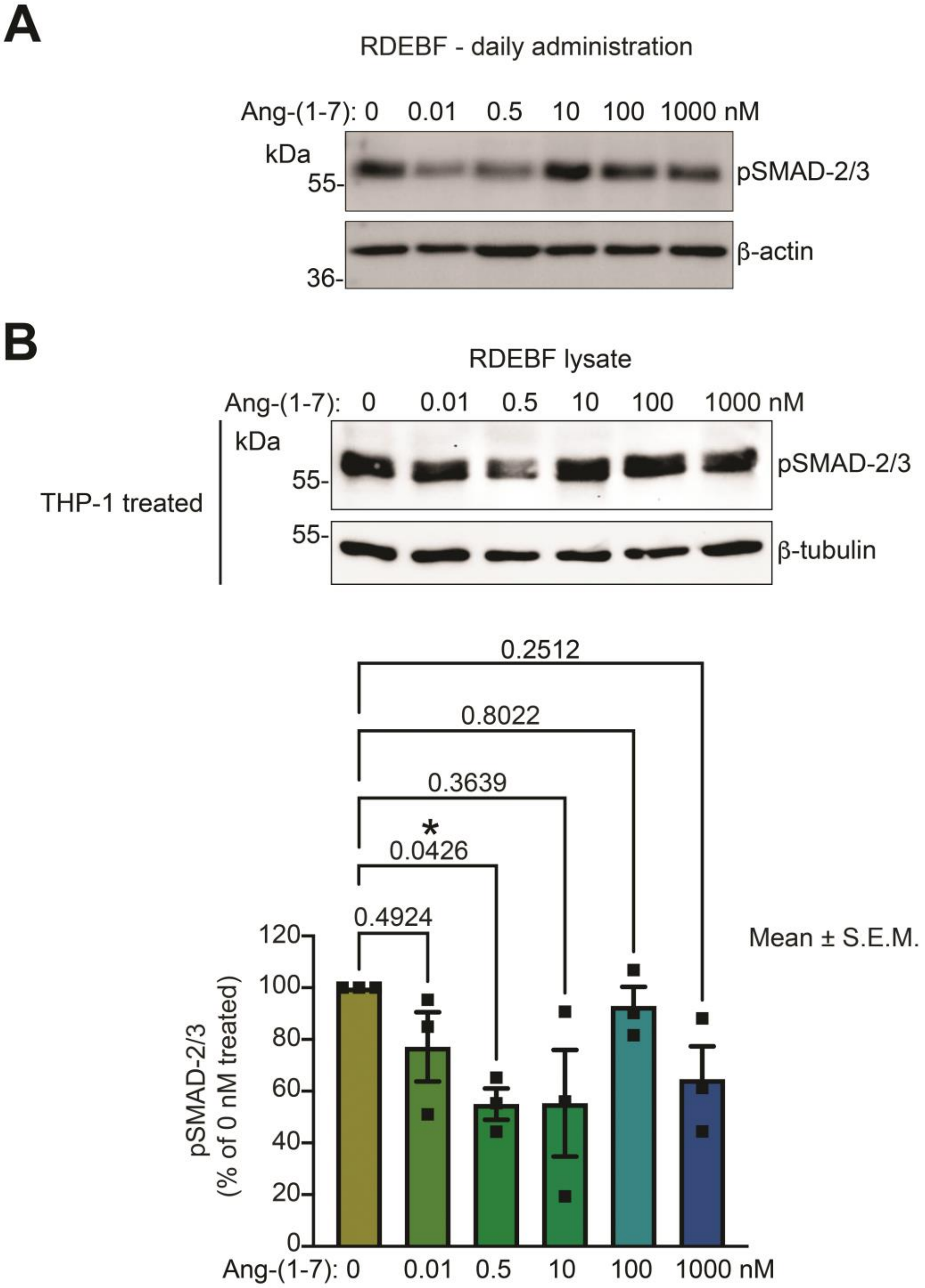

Appendix Fig S8. Ang-(1-7) evokes a bell-shaped concentration-dependent response. (**A**) Monocultures of RDEBF treated with the indicated Ang-(1-7) concentrations daily for two days and analyzed by western blotting for pSMAD-2/3. β-actin was used as loading control. (**B**) Western blotting of cell and matrix lysates for RDEBF co-cultured with THP-1 monocytes for two days, prior of which THP-1 monocytes had been treated with the indicated Ang-(1-7) daily for two days. Blots show pSMAD-2/3 (pSer 465/467 SMAD-2 / pSer 423/425 SMAD-3) and β-tubulin as loading control. The graph below shows values from densitometric quantification of blots from three experiments Individual data points, mean ± S.E.M are shown. Data were analyzed by one-way ANOVA with Dunnett's correction. *P* values < 0.05 are considered significant.

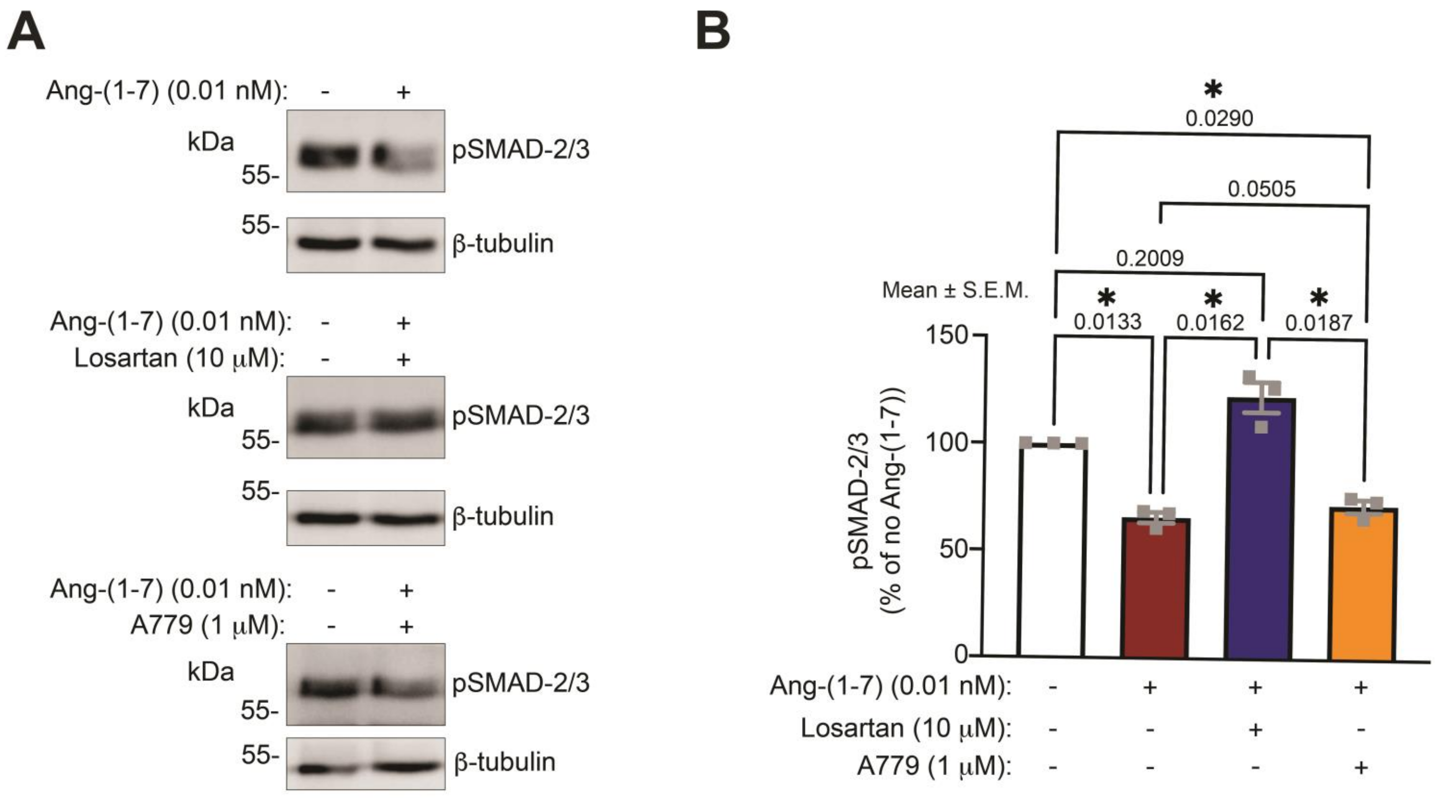

Appendix Fig S9. AT1R mediates the response to low Ang-(1-7) doses in fibroblasts. Subconfluent RDEBF were treated with the AT1R antagonist losartan (10 μM) or the MAS receptor inhibitor A779 (1 μM) for 1h before addition of 0.01 nM Ang-(1-7). (**A**) One hour after addition of Ang-(1-7) the cells were harvested and analyzed by western blotting for pSMAD-2/3 using β-tubulin as loading control. (**B**) Densitometric quantification of blots as in A from treatment of three different RDEBF donors. The abundance of pSMAD-2/3 was normalized to β-tubulin and expressed as the percentage of untreated (-). Individual data points, mean ± S.E.M are shown. The data were analyzed by one-way ANOVA with Tukey's correction. *P* values < 0.05 are considered significant.

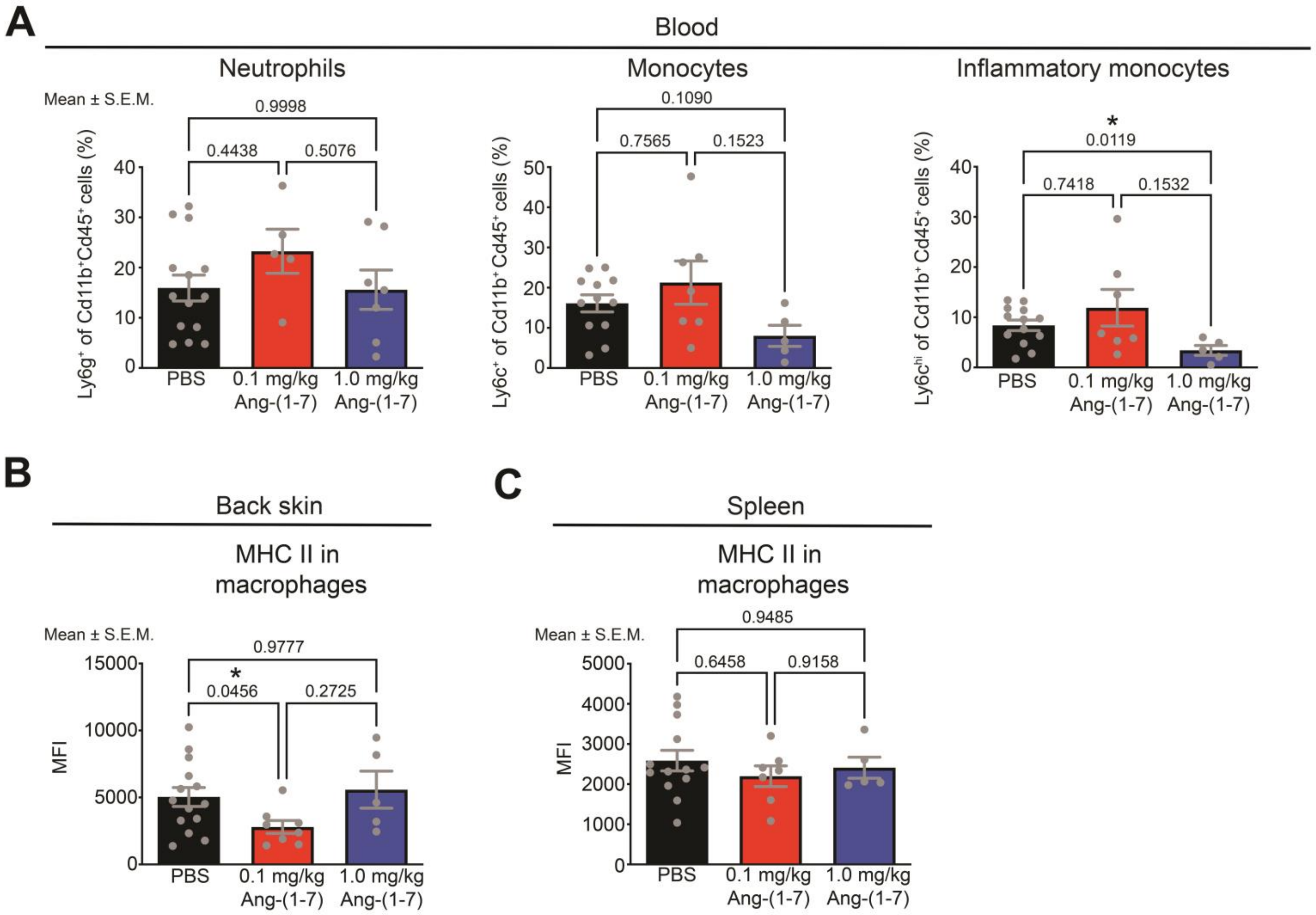

Appendix Fig S10. Ang-(1-7) subtly affects inflammation in back skin in RDEB mice. (**A**) Flow cytometry analyses of neutrophils, monocytes and inflammatory monocytes (high Ly6C expressing) in blood from RDEB mice treated with daily injections of 0.1, 1.0 mg/kg Ang-(1-7) or PBS for seven weeks. (**B**) The mean fluorescence intensity (MFI) of MHC II staining on macrophages from back skin of mice treated as in A. (**C**) MHC II staining on macrophages isolated from spleen from mice treated as in A. Individual data points, mean ± S.E.M are shown. The data were analyzed by one-way ANOVA with Tukey's correction. *P* values < 0.05 are considered significant. N = 5-14 mice.

A

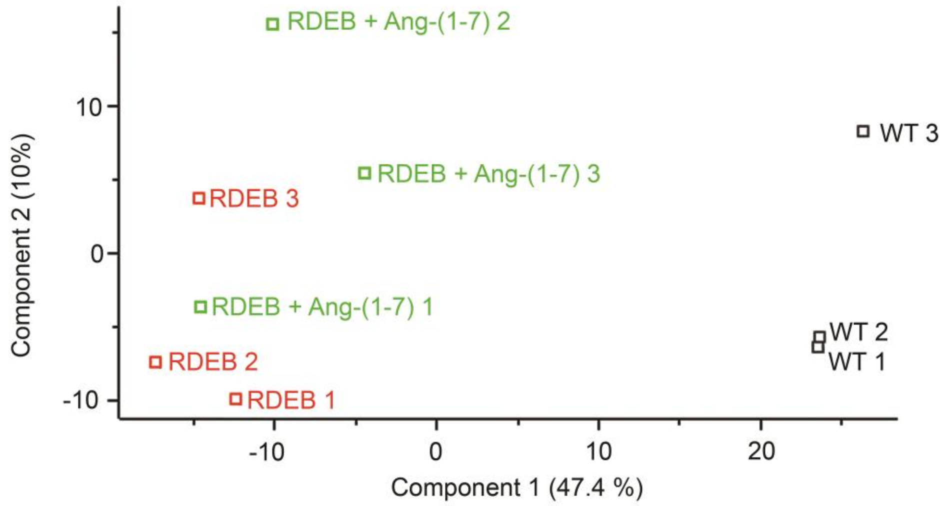

B

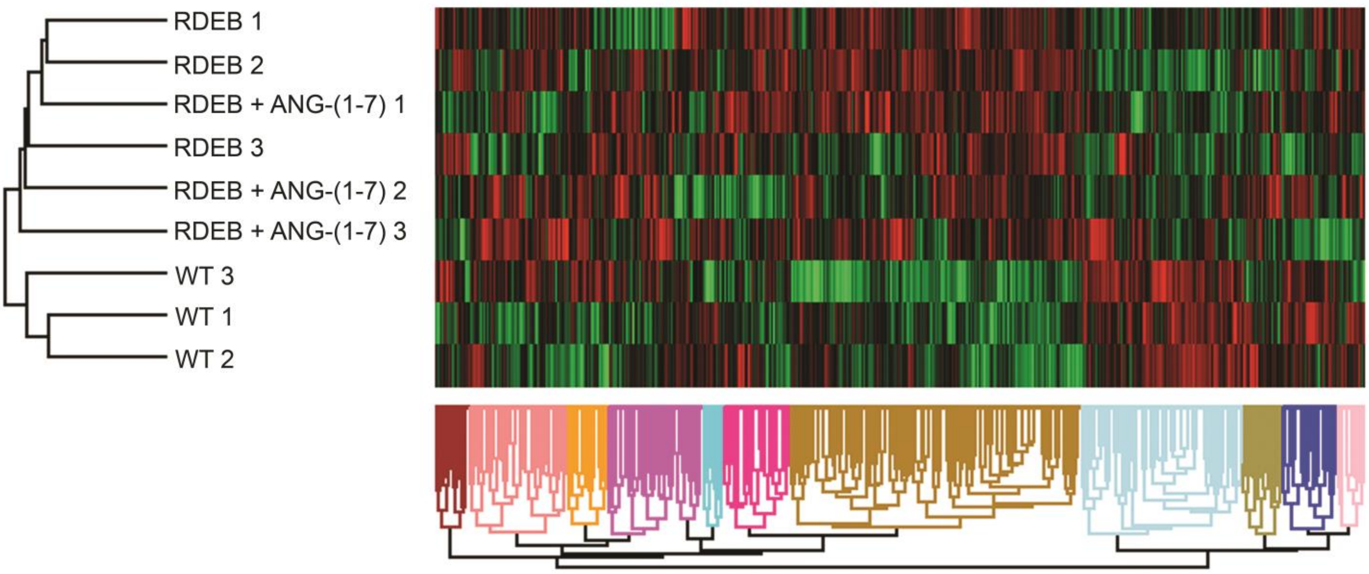

Appendix Fig S11. High-dose of Ang-(1-7) has no global molecularly discernable protective effect on the dermal proteome of RDEB mice. (**A**) Principal component analysis plot of forepaw skin lysates from WT mice or RDEB mice treated with daily injections of 1.0 mg/kg Ang-(1-7) or PBS for seven weeks analyzed by MS-based proteomics. Note that WT samples are distinctly separated from RDEB mouse samples but RDEB samples from mice treated with PBS or Ang-(1-7) do not separate. (**B**) Heat map from hierarchical clustering of protein abundances for samples as in A. Again WT samples clearly separate from RDEB samples but samples from Ang-(1-7) treated mice are intertwined with samples from RDEB mice that had received PBS injections only.
