# Supplementary material for: Pro‐inflammatory immunity supports fibrosis advancement in epidermolysis bullosa: intervention with Ang‐(1‐7)
Source: EMBO Mol Med. 2021 Aug 30;13(10):e14392. doi: 10.15252/emmm.202114392 (PMC8495454; doi:10.15252/emmm.202114392)
Supplement: Supplementary file 2 — Expanded View Figures PDF [file EMMM-13-e14392-s013.pdf]

Expanded View Figures

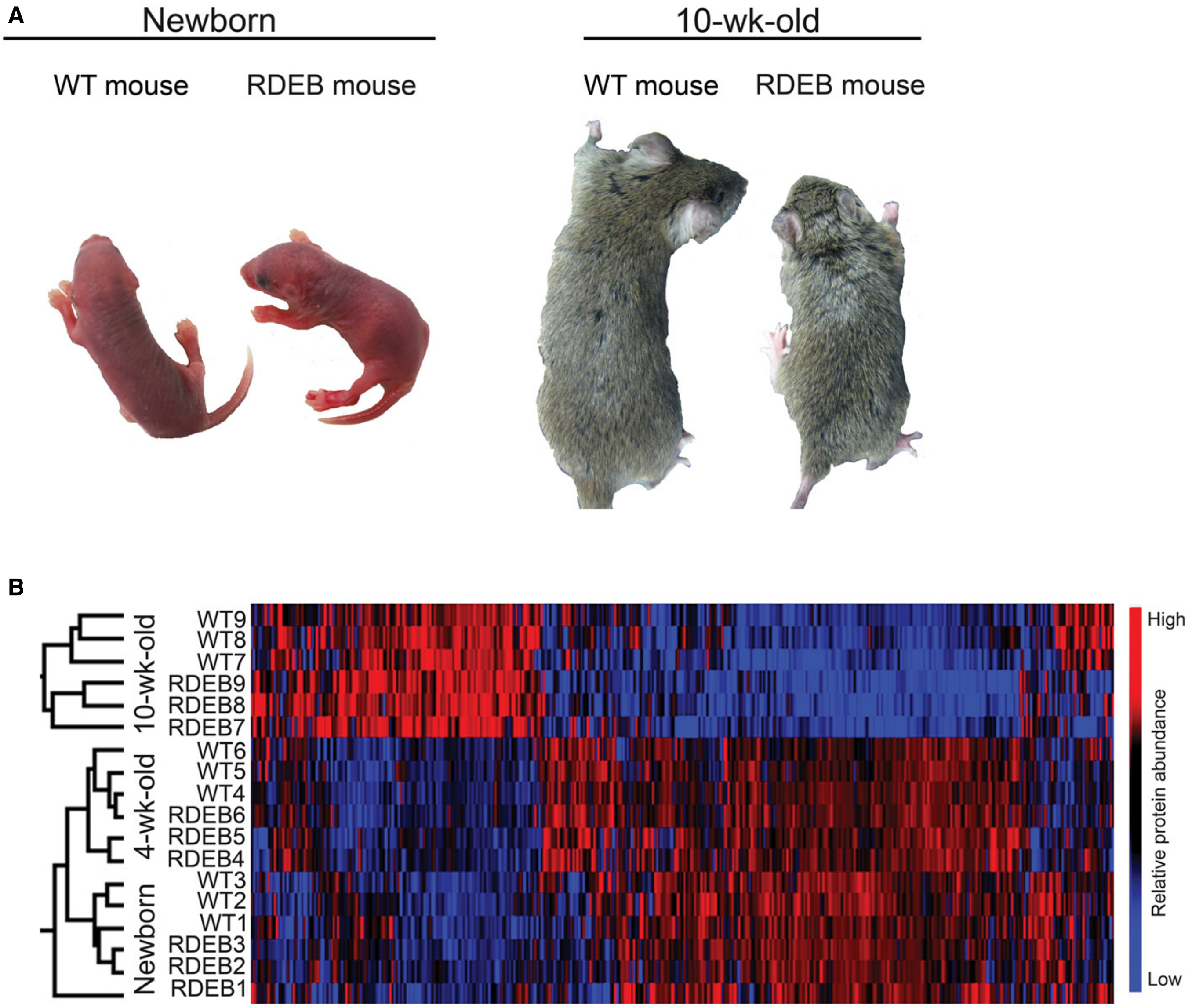

Figure EV1. RDEB mouse back skin does not display overt injury or subsequent fibrosis.

A Photographs of back skin from WT and RDEB mice of the indicated ages.

B Hierarchical clustering of back skin samples of the indicated ages and genotypes based on protein abundances determined by label-free quantification MS analysis. Limited differences are seen between age-matched WT and RDEB mice, and most notable differences occur between ages.

© 2021 The Authors EMBO Molecular Medicine 13: e14392 | 2021 21 of 20

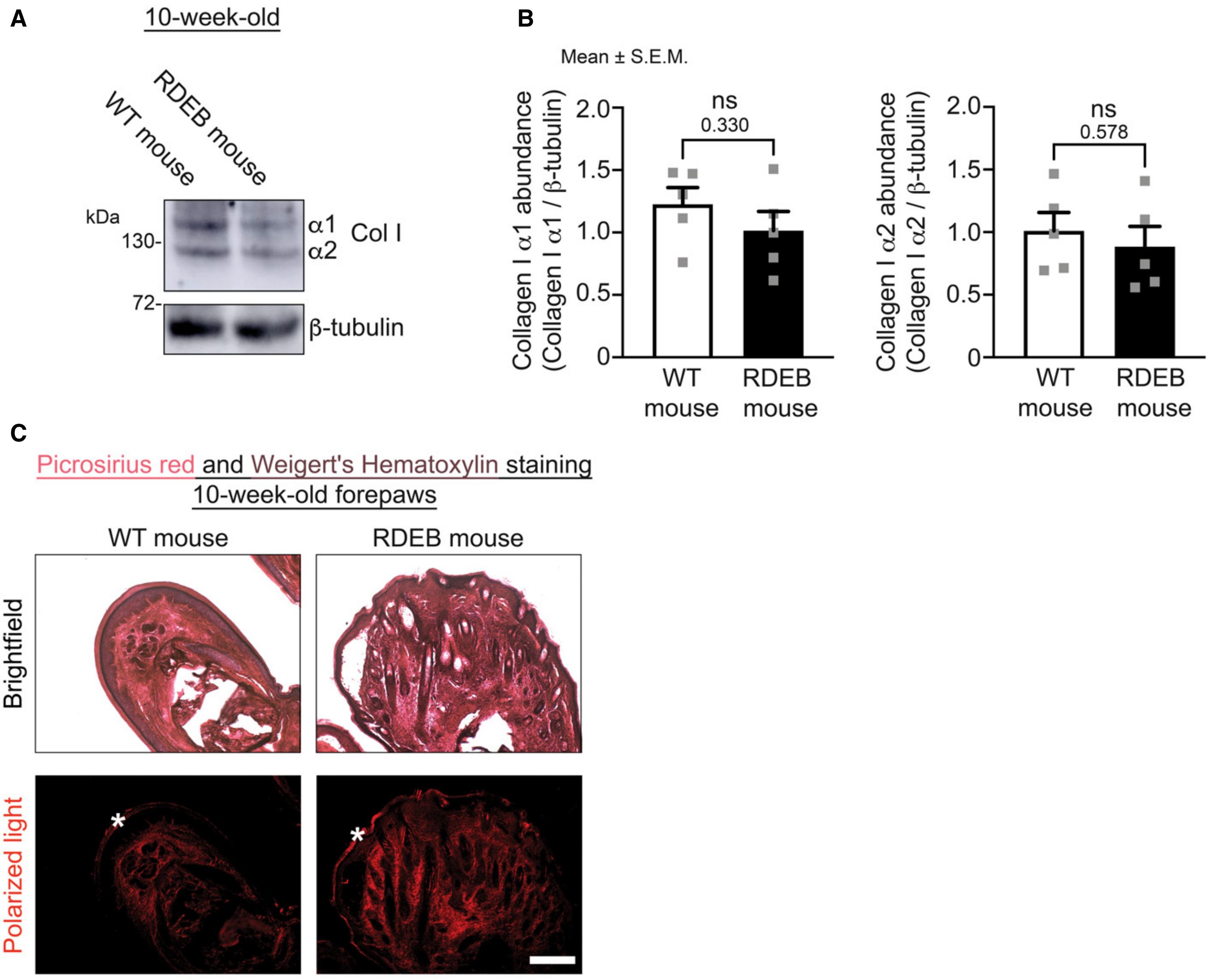

Figure EV2. Collagen I abundance is not increased in fibrotic RDEB mouse skin but arrangement of fibrillar collagens is altered.

A Western blot analysis of protein lysates from forepaws of 10-wk-old WT and RDEB mice for collagen I. β-tubulin was used as a loading control.

B Densitometric quantification of collagen I α1 (left) or α2 (right) polypeptide abundance normalized to expression of β-tubulin. $N = 5$ forepaws from 10-wk-old WT and RDEB mice. Individual values, mean ± SEM are shown. Data were tested with unpaired *t*-test. *P* values are indicated, ns = not significant.

C Images of sections of forepaws from 10-wk-old WT and RDEB mice stained for picrosirius red and Weigert's hematoxylin taken under brightfield (top, picrosirius red = pink-red and Weigert's hematoxylin = brown) or under polarized light (bottom). Increased red staining under polarized light indicates thickened collagen fibrils or their increased parallel arrangement. Scale bar = 100 µm. Asterisks indicate unspecific epidermal staining.

 © 2021 The Authors

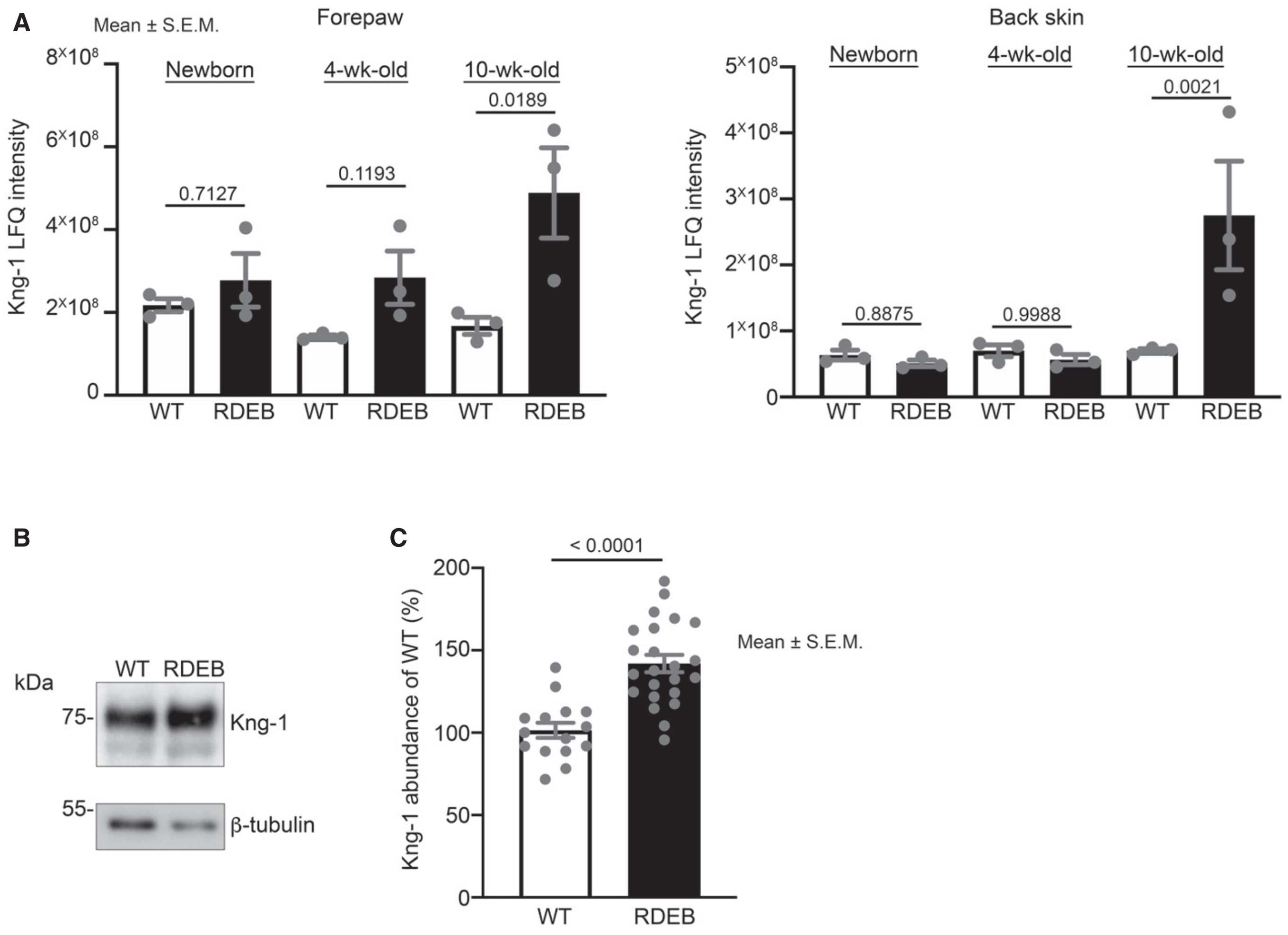

Figure EV3. Kininogen-1 is increased in injured RDEB mouse forepaws.

A Plotted are intensity values of kininogen-1 derived from label-free quantification (LFQ) of whole back skin and forepaw protein lysates analyzed by label-free MS-based proteomics. Analyzed were forepaws from newborn, 4-week-old, and 10-week-old WT and RDEB mouse littermates. These ages represent initial injury, mid-stage fibrosis, and late-stage fibrosis. $N = 3$ per genotype and age. Statistics calculated by LIMMA and the resulting P values are shown. Individual data points, mean ± SEM, are shown.

B Validation of proteomics by Western blotting. Representative Western blots for kininogen-1 of forepaw protein lysates from 10-week-old WT and RDEB mice are shown. The blots were probed with β-tubulin as a loading control.

C Densitometric quantification of Western blots as in B. Kininogen-1 abundance was normalized to β-tubulin and expressed as percentage abundance in paired WT samples. Individual data points, mean ± SEM, are shown, $n = 8$ forepaws from 8 different mice per genotype. $P < 0.0001$ (unpaired t-test).

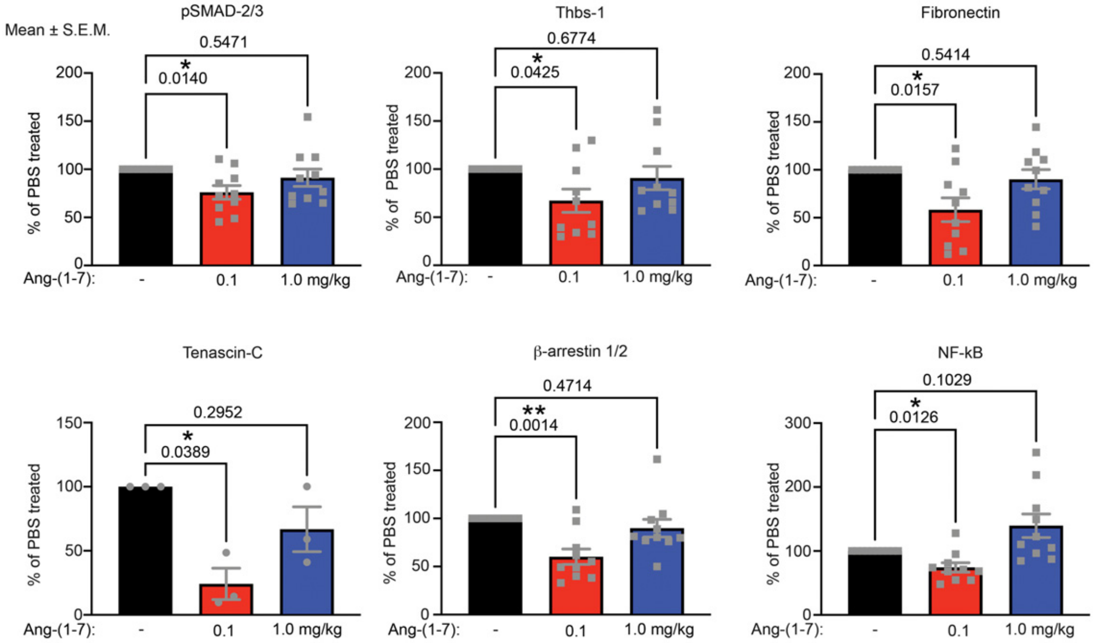

Figure EV4. Low dose of Ang-(1-7) significantly alters abundances of proteins important for dermal homeostasis.

Densitometric quantification of Western blots shown in Fig 7C of forepaw lysates from RDEB mice treated with daily injections of 0.1, 1.0 mg/kg Ang-(1-7), or PBS for seven weeks. Quantifications after normalization to β-tubulin or β-actin for pSMAD-2/3 (pSer 465/467 SMAD-2/pSer 423/425 SMAD-3), thrombospondin-1 (Thbs-1), fibronectin, tenascin-C, β-arrestin-1/2, and NF-κB are shown. Individual data points from individual mice, mean ± SEM, are shown. Data are expressed as the percentage abundance of PBS-treated and were analyzed by one-way ANOVA with Dunnett's correction. *P* values < 0.05 are considered significant. $N = 6$–8 paws (one paw per mouse) per condition.

© 2021 The Authors

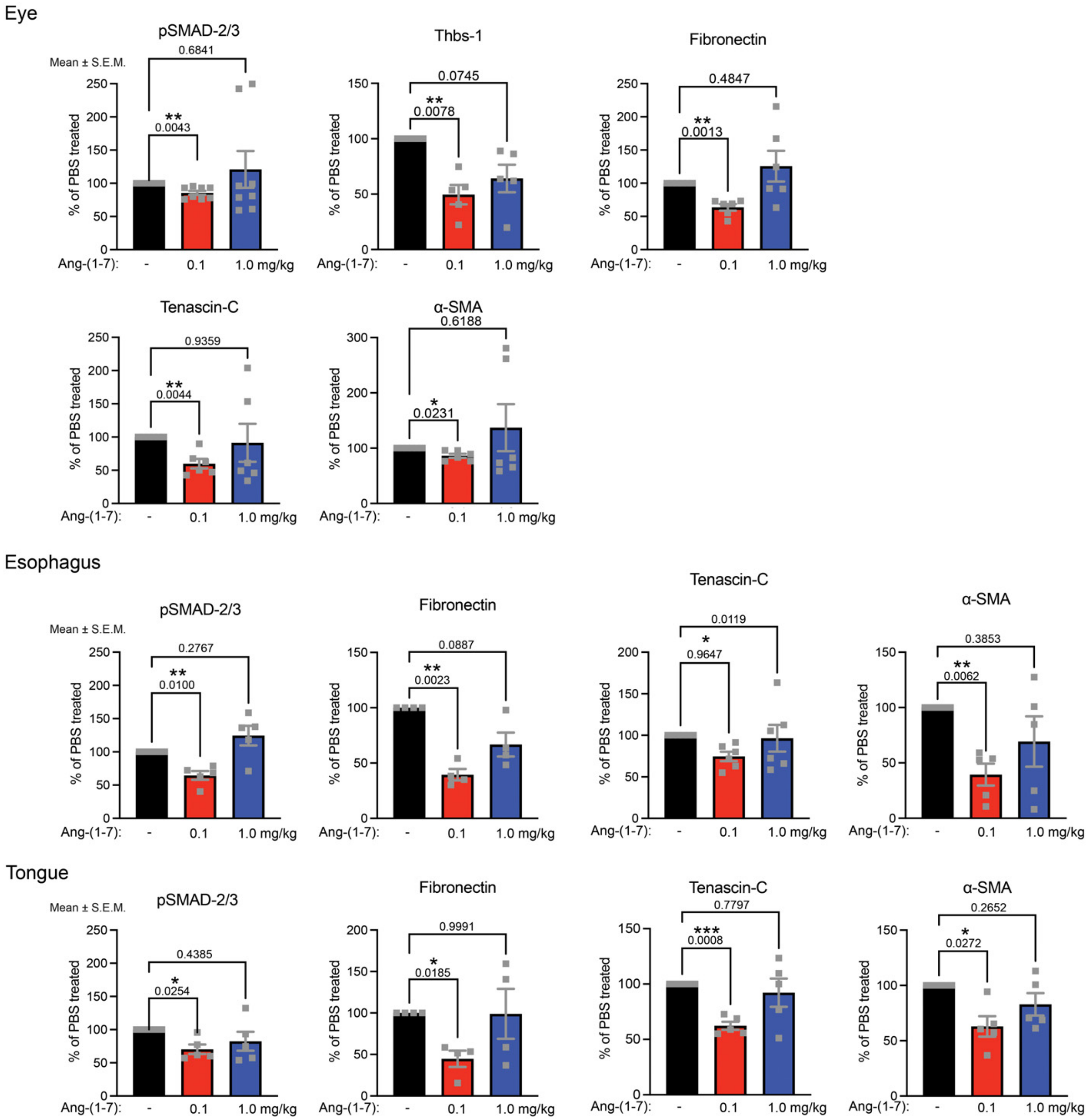

Figure EV5. Low dose of Ang-(1-7) significantly alters abundances of proteins important for homeostasis of eye, esophagus and tongue.

Densitometric quantification of Western blots shown in Fig 7E of whole eye, esophagus, and tongue lysates from RDEB mice treated with daily injections of 0.1, 1.0 mg/kg Ang-(1-7), or PBS for 7 weeks. Quantifications after normalization to β-tubulin for pSMAD-2/3 (pSer 465/467 SMAD-2/pSer 423/425 SMAD-3), thrombospondin-1 (Thbs-1), fibronectin, tenascin-C, and α-smooth muscle actin (α-SMA). Individual data points from individual mice, mean ± SEM, are shown. Data were analyzed by one-way ANOVA with Dunnett's correction. *P* values < 0.05 are considered significant. *N* = 4 organs from four different mice per condition.

© 2021 The Authors
