# Supplementary figures and images for: Pro‐inflammatory immunity supports fibrosis advancement in epidermolysis bullosa: intervention with Ang‐(1‐7)
Source: EMBO Mol Med. 2021 Aug 30;13(10):e14392. doi: 10.15252/emmm.202114392 (PMC8495454; doi:10.15252/emmm.202114392)

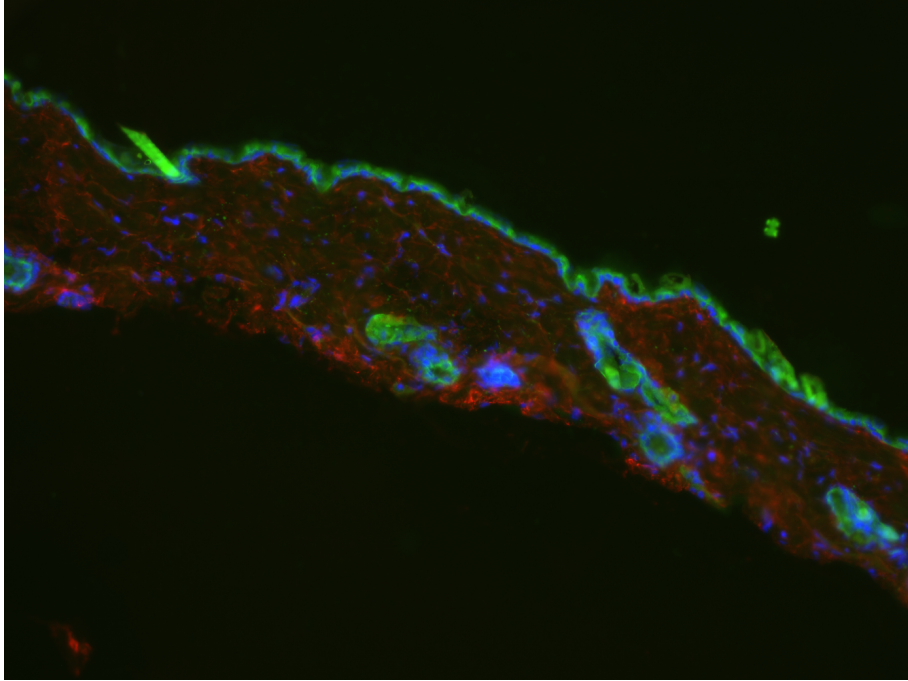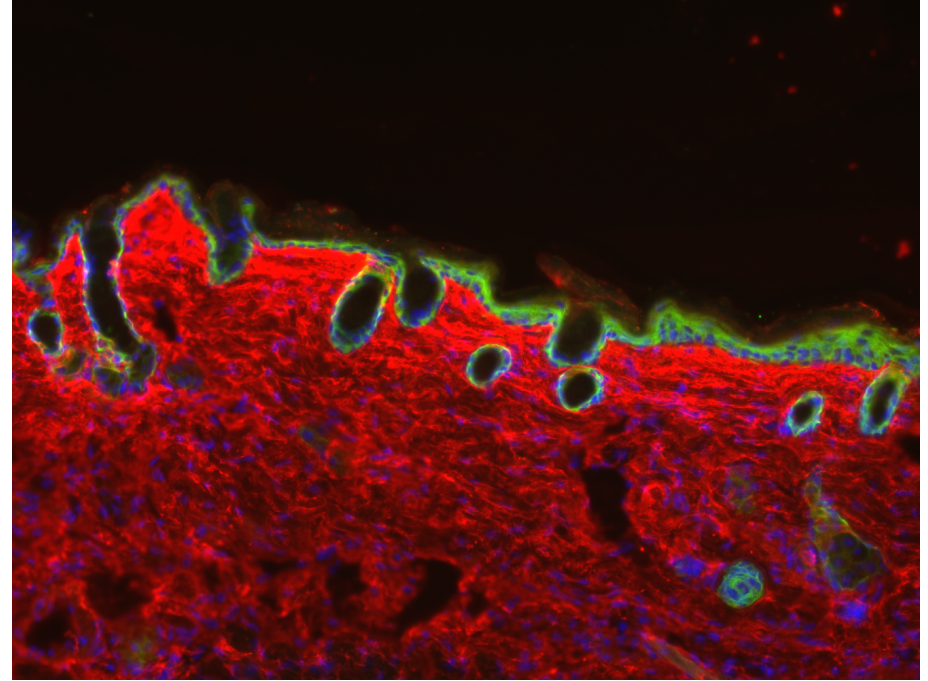

**Figure 2J**

Supplement: Supplementary file 9 — Source Data for Figure 2 [file EMMM-13-e14392-s011.pdf]

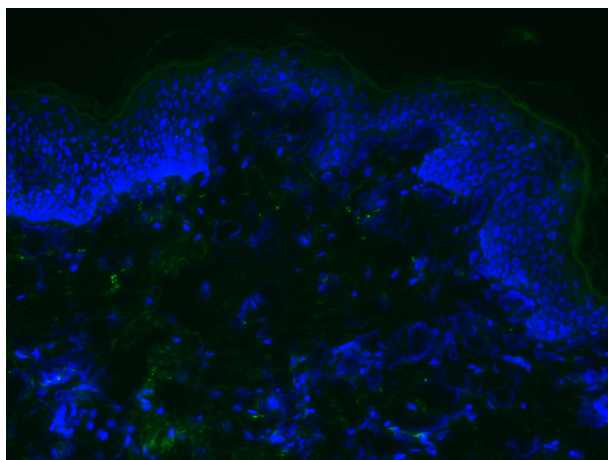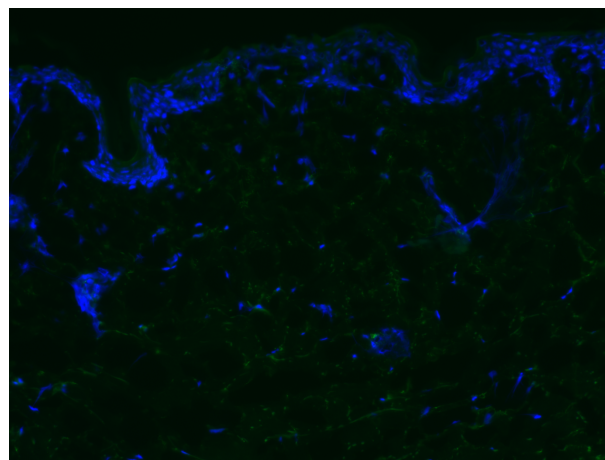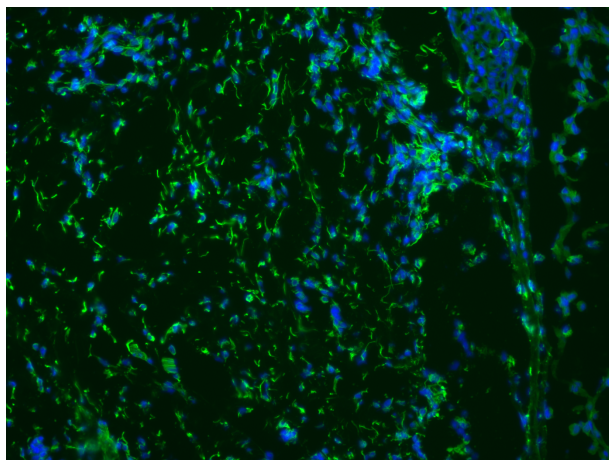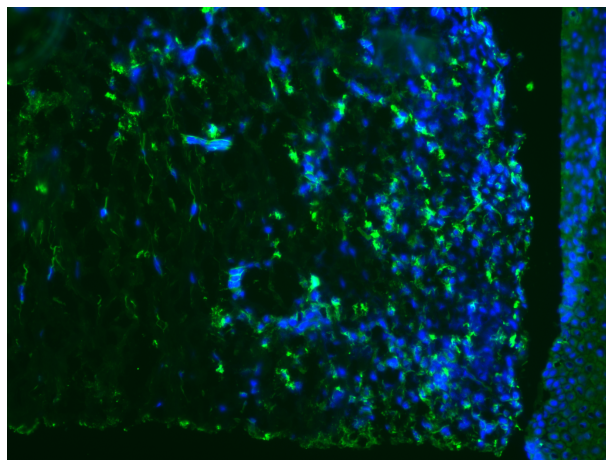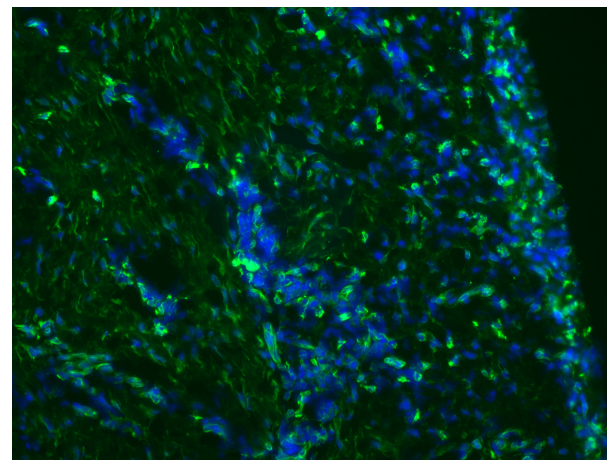

**Figure 3A**

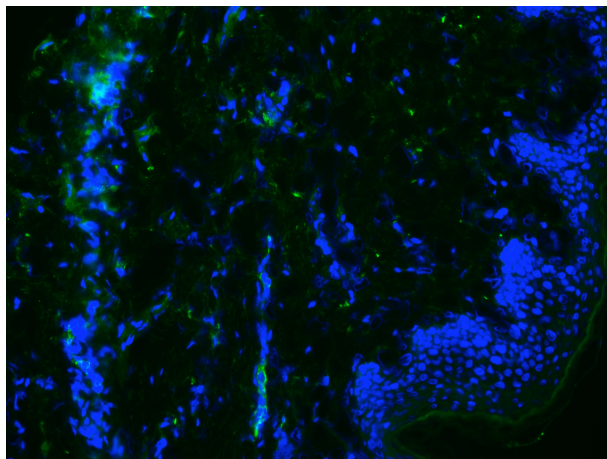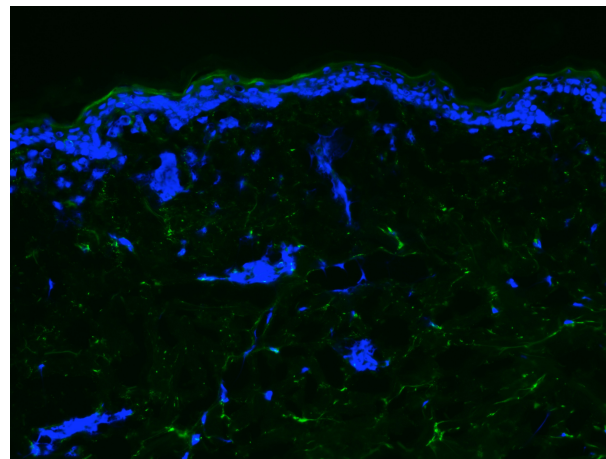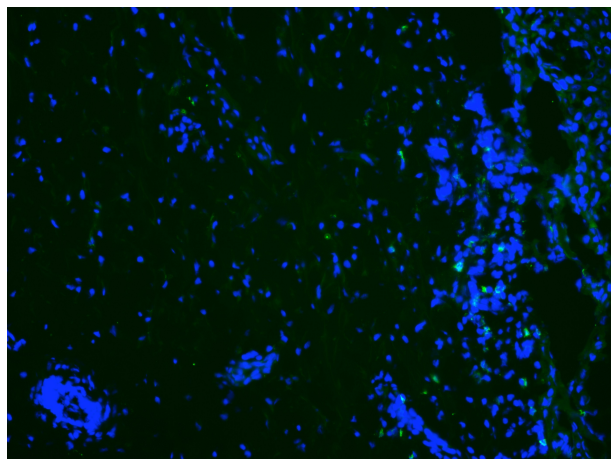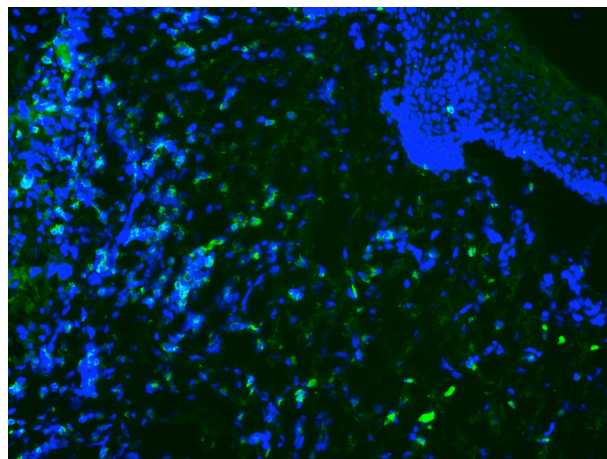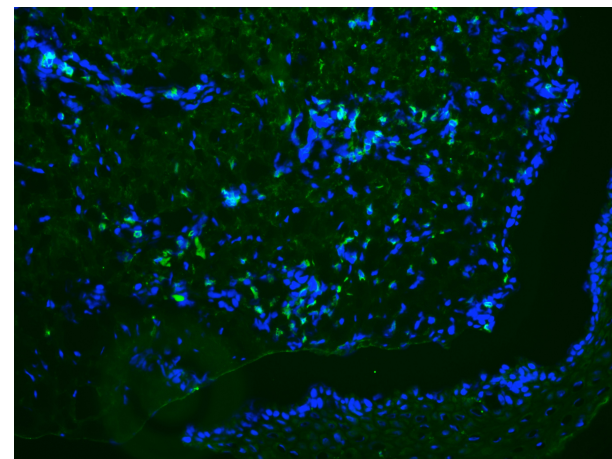

**Figure 3B**

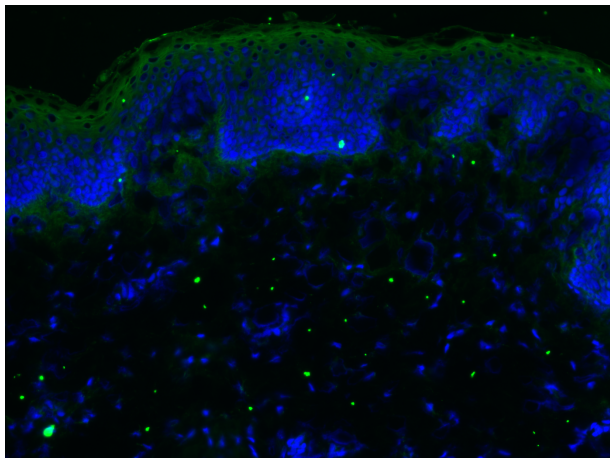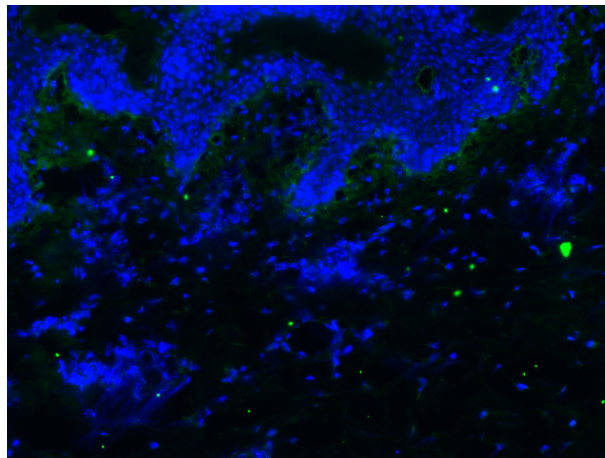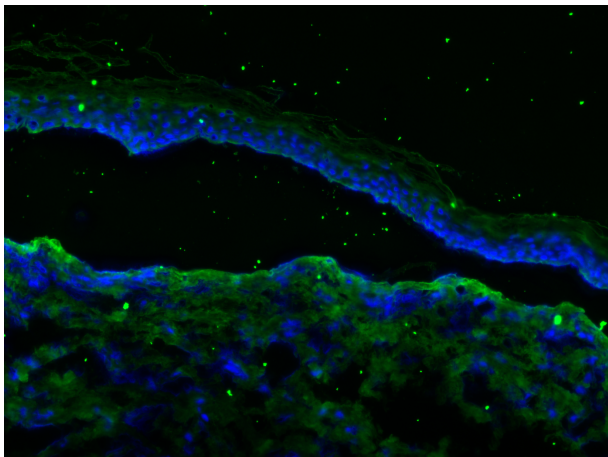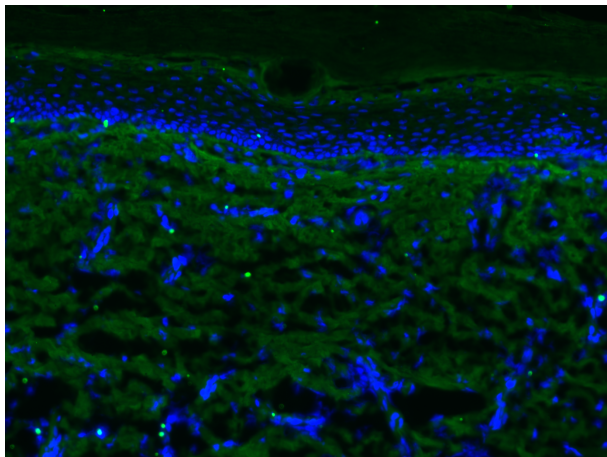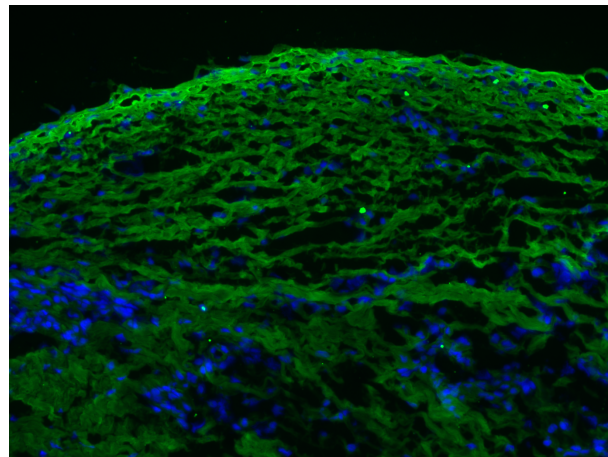

**Figure 3C**

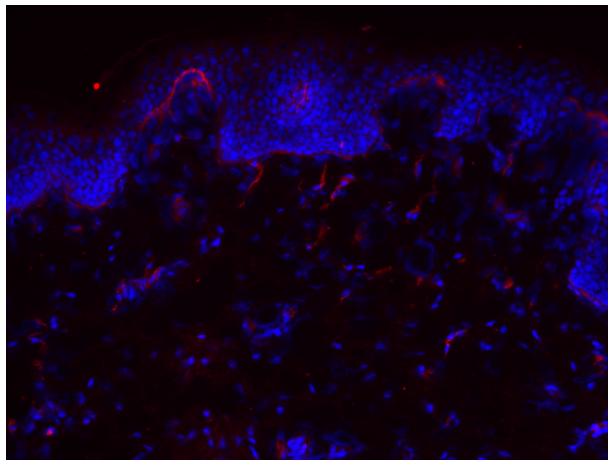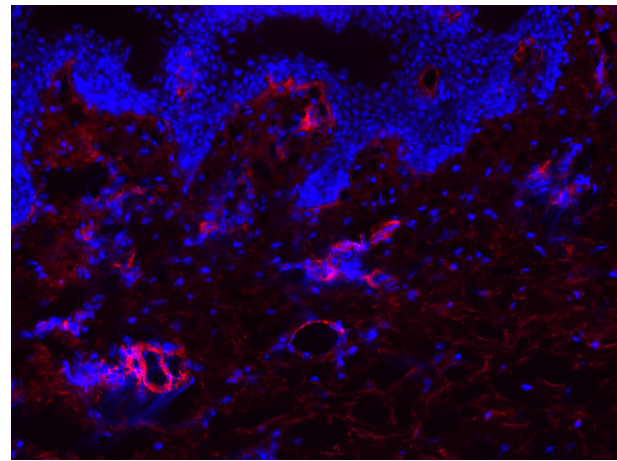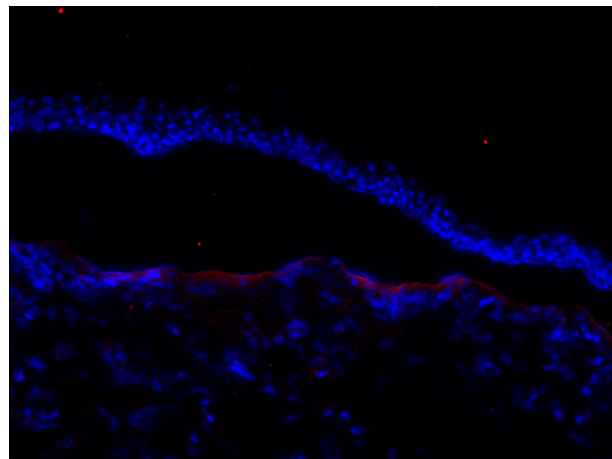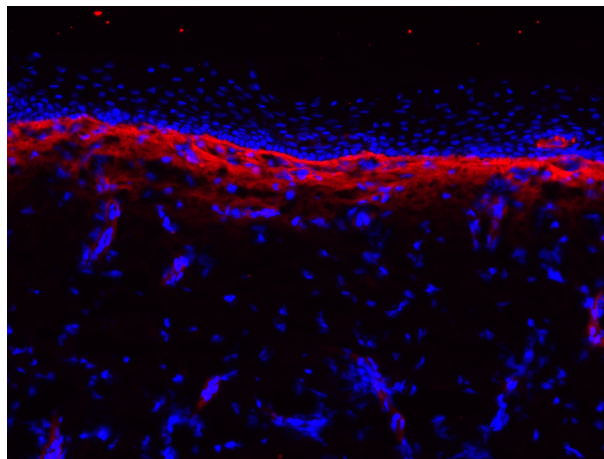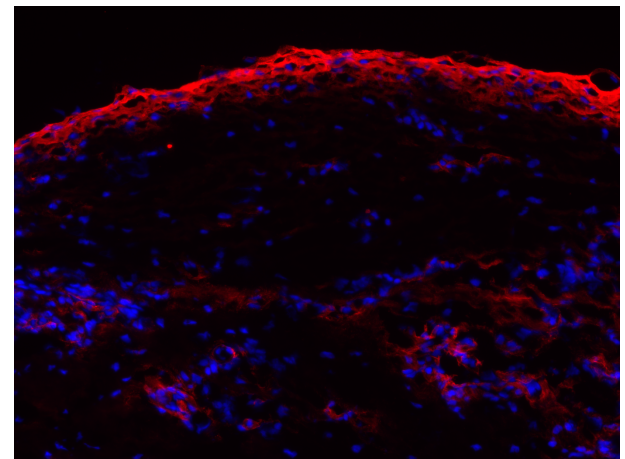

**Figure 3D**

Supplement: Supplementary file 10 — Source Data for Figure 3 [file EMMM-13-e14392-s004.pdf]

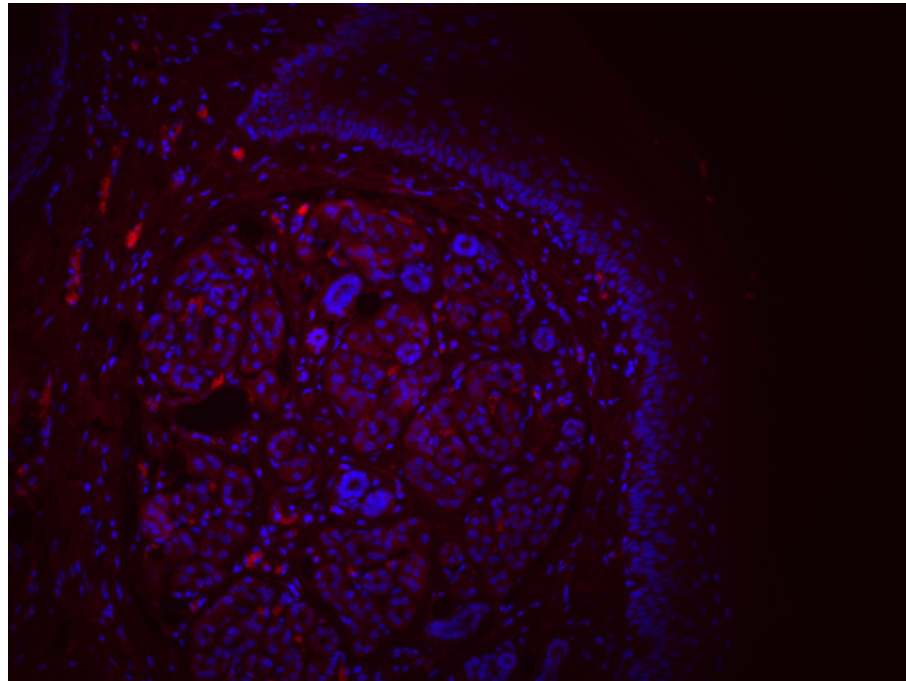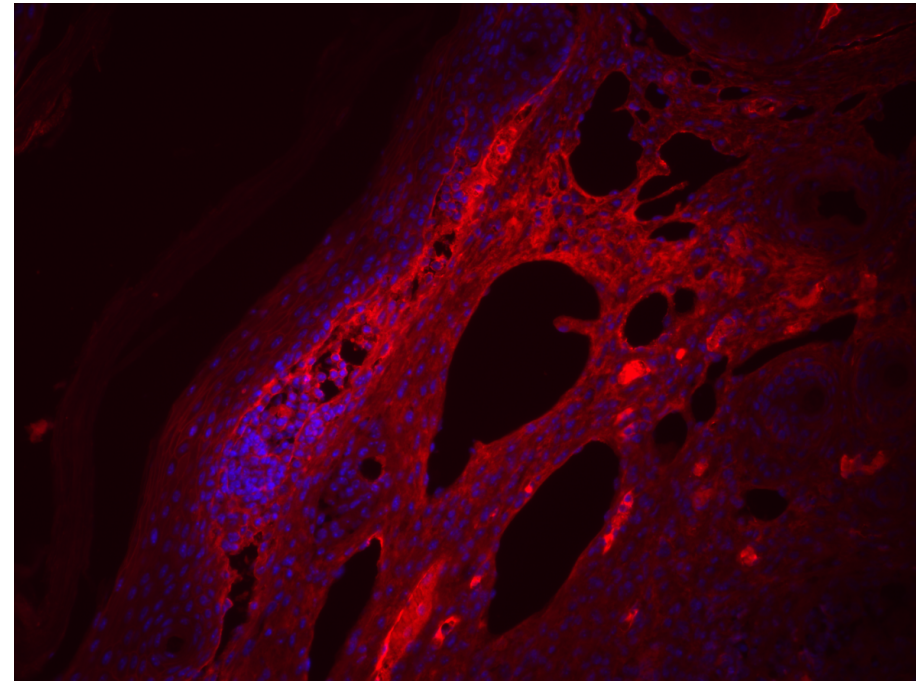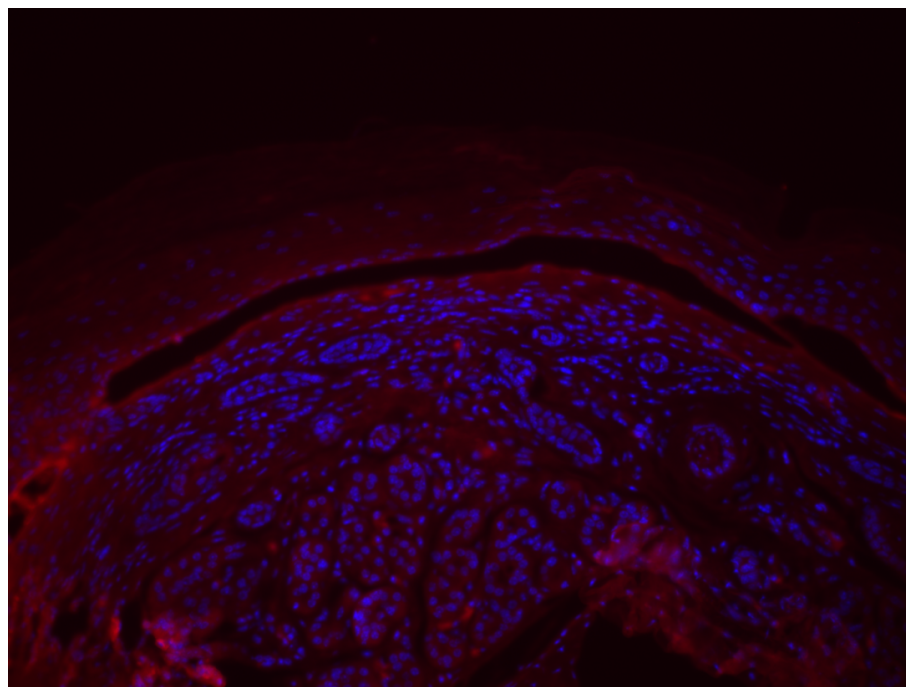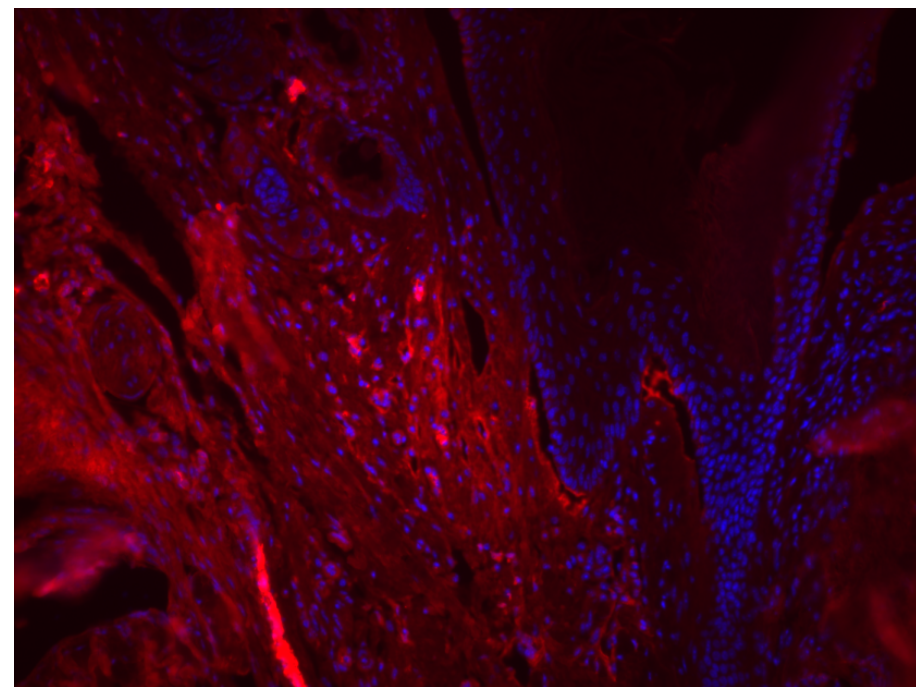

Figure 6F

Supplement: Supplementary file 12 — Source Data for Figure 6 [file EMMM-13-e14392-s001.pdf]
