# Supplementary material for: Pro‐inflammatory immunity supports fibrosis advancement in epidermolysis bullosa: intervention with Ang‐(1‐7)
Source: EMBO Mol Med. 2021 Aug 30;13(10):e14392. doi: 10.15252/emmm.202114392 (PMC8495454; doi:10.15252/emmm.202114392)
Supplement: Supplementary file 11 — Source Data for Figure 4 [file EMMM-13-e14392-s003.pdf]

Fibronectin

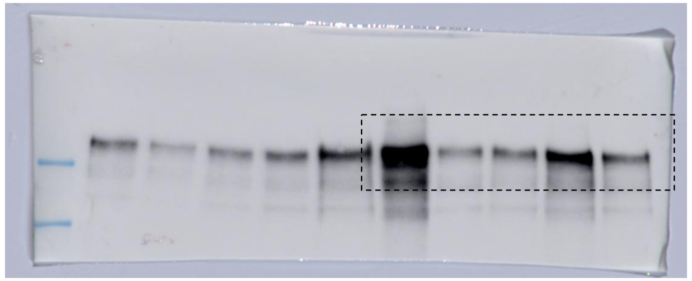

pSMAD-2/3

Same blot cut in two

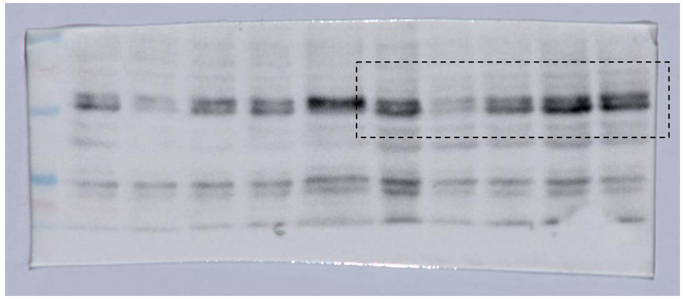

THBS-1

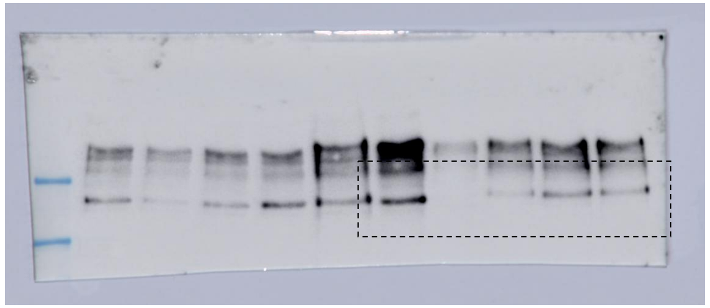

β-tubulin

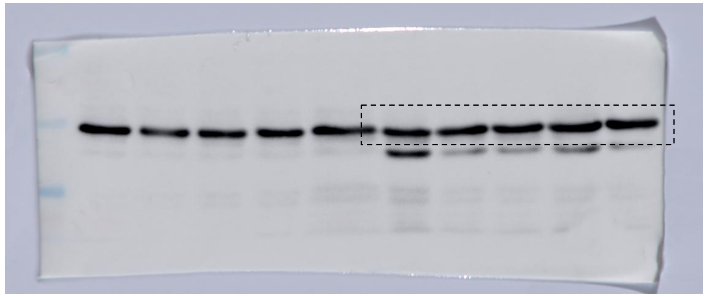

Figure 4A

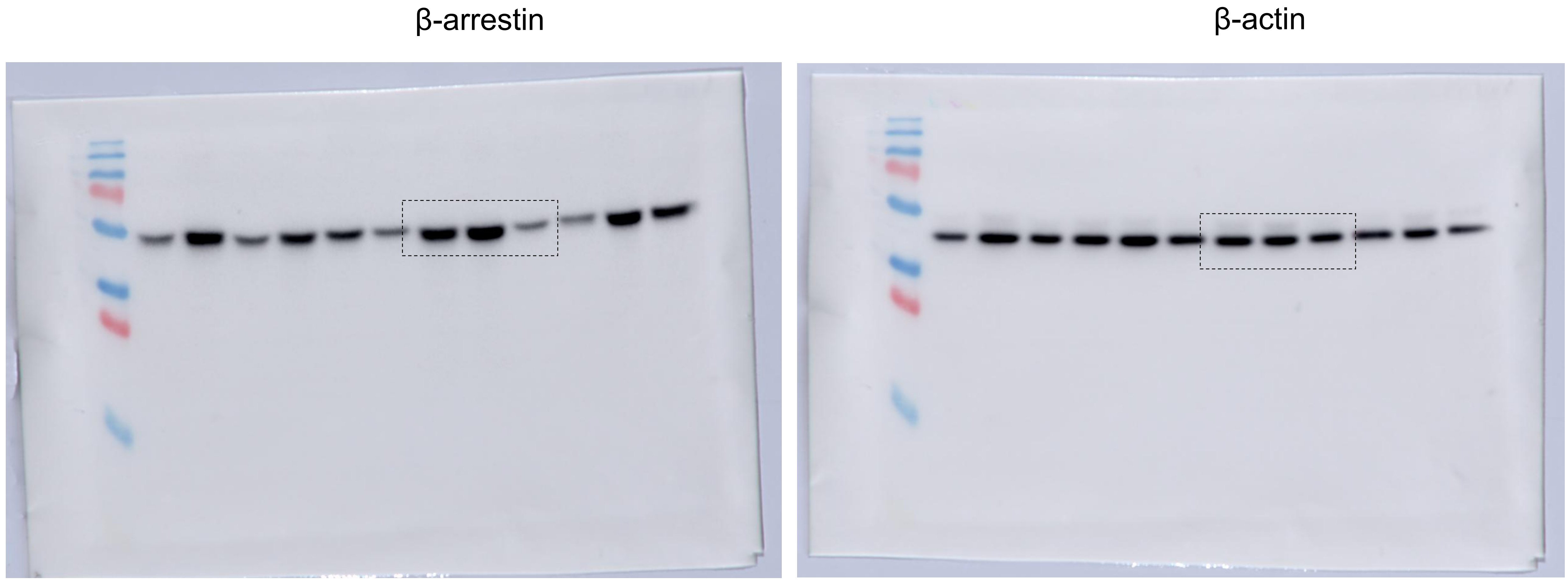

Figure 4C

pAKT

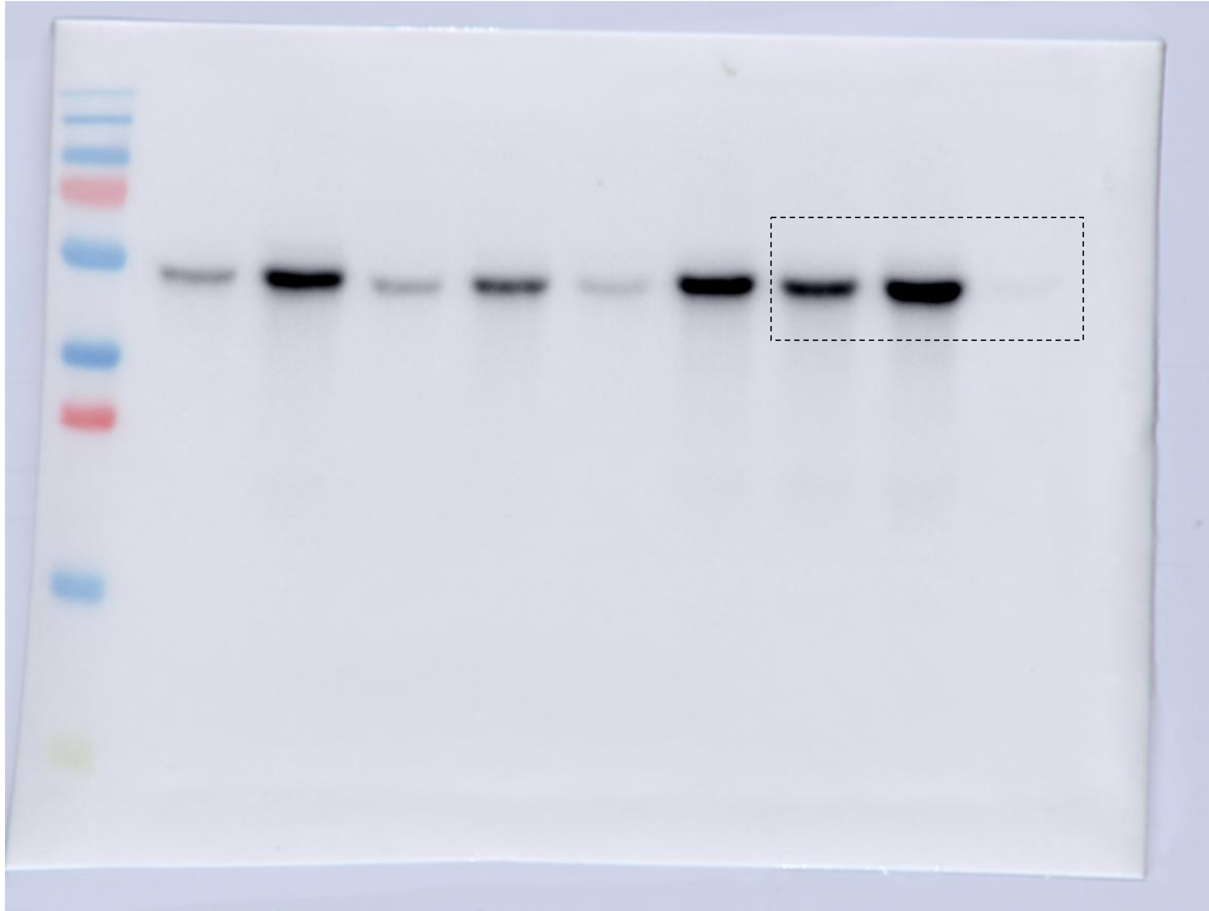

β-actin

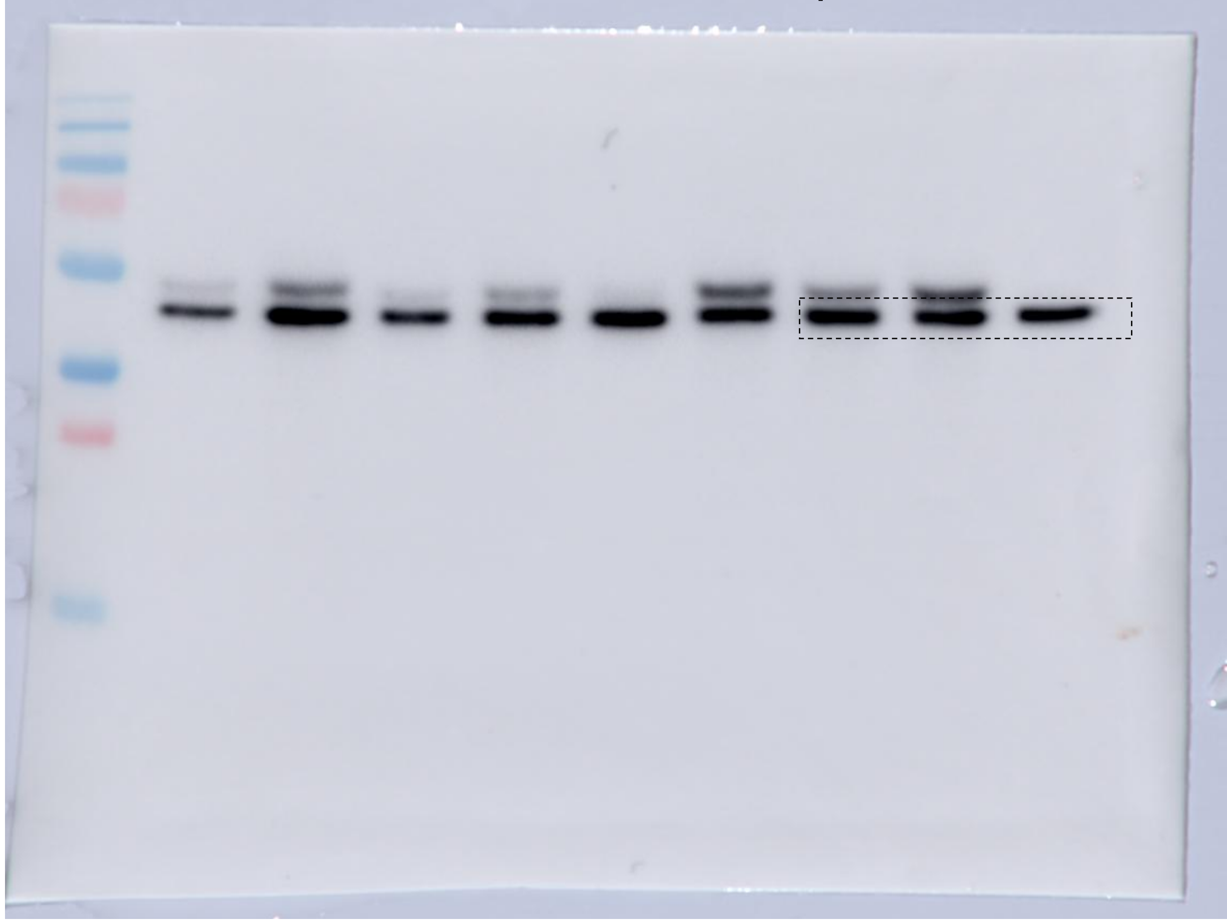

Figure 4C

pSMAD-2/3

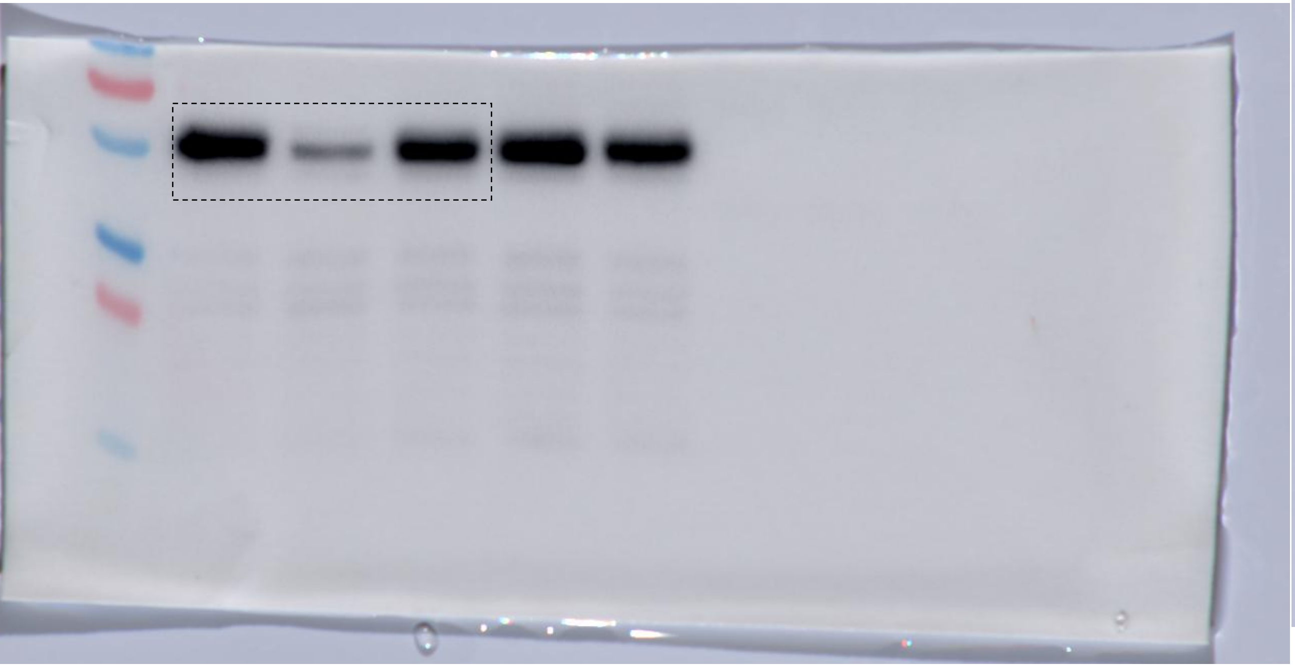

β-tubulin

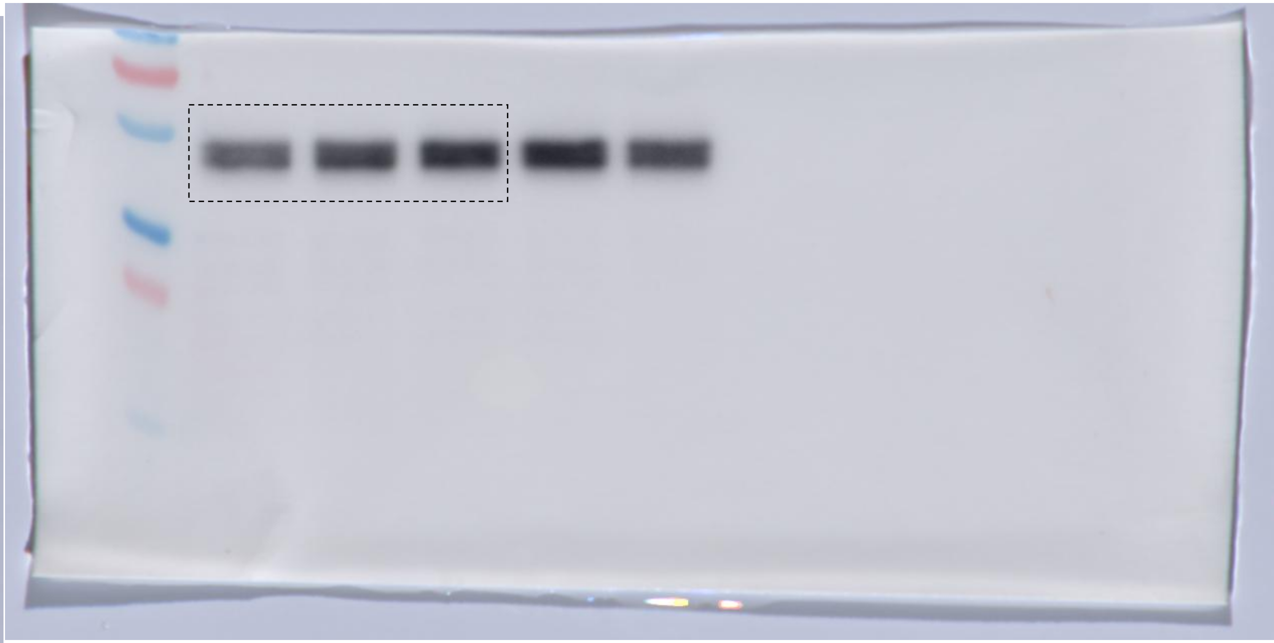

Figure 4C

pERK1/2

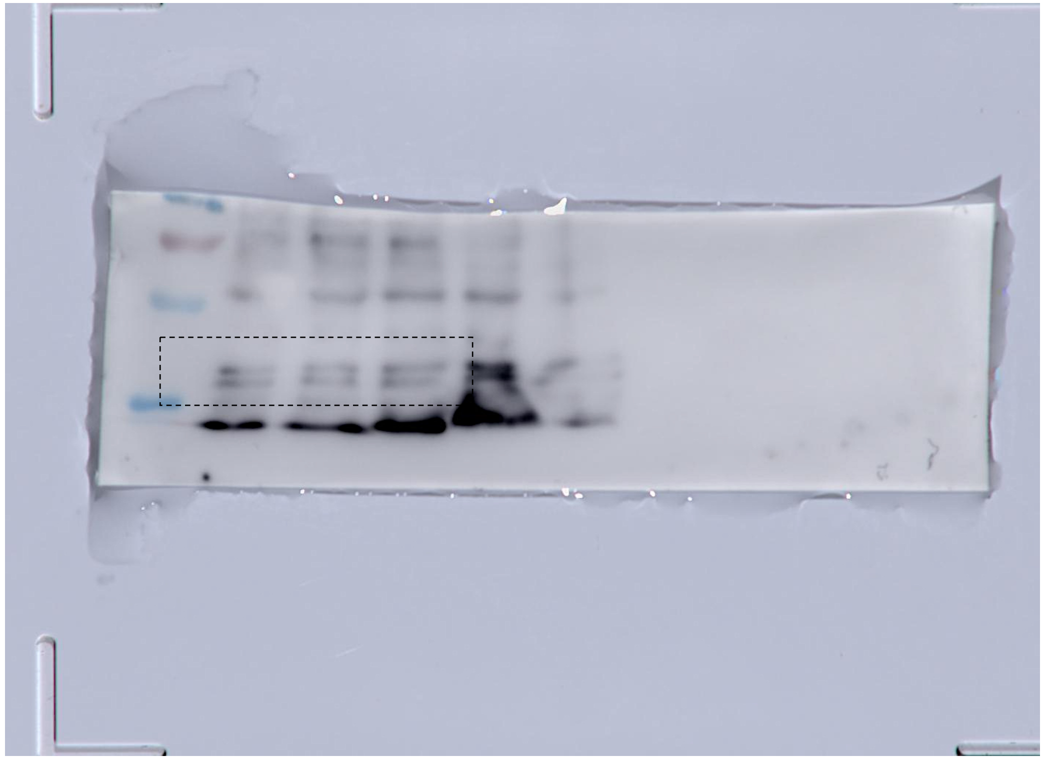

β-tubulin

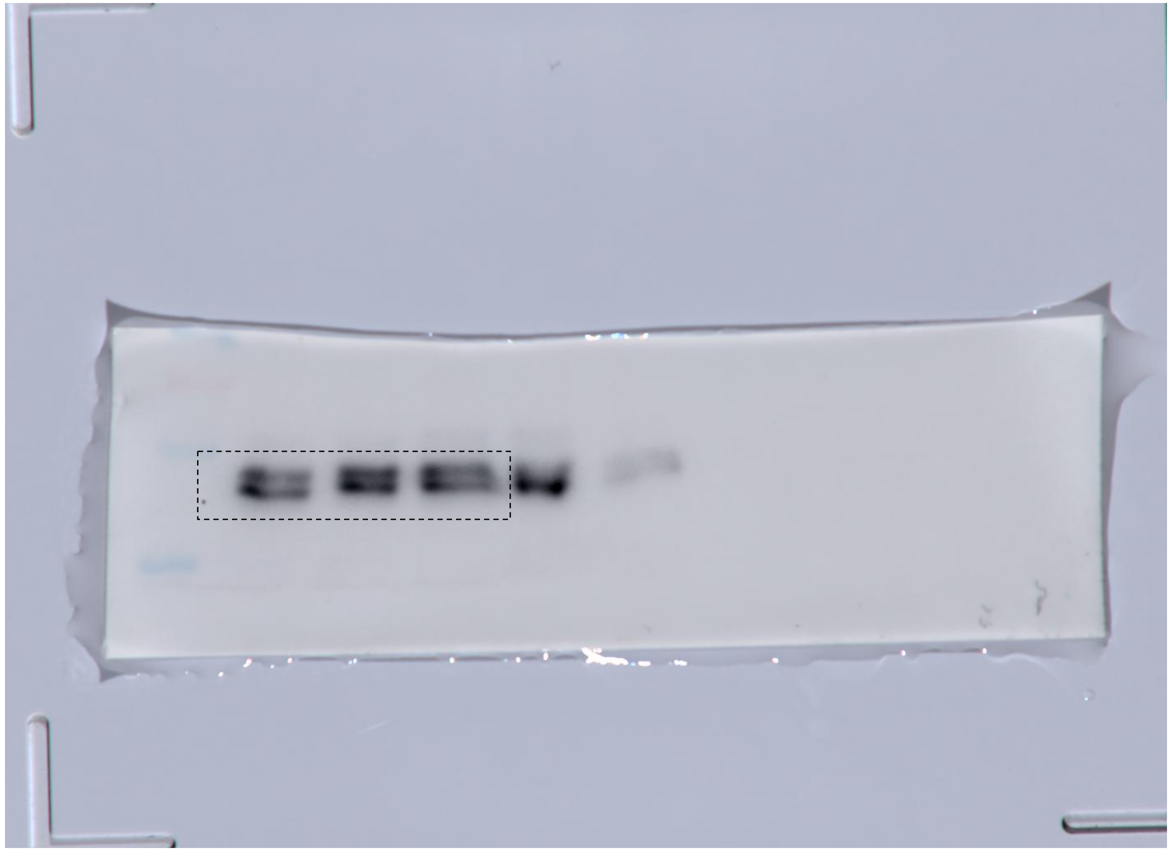

Figure 4C

Pro-IL1β

β-tubulin

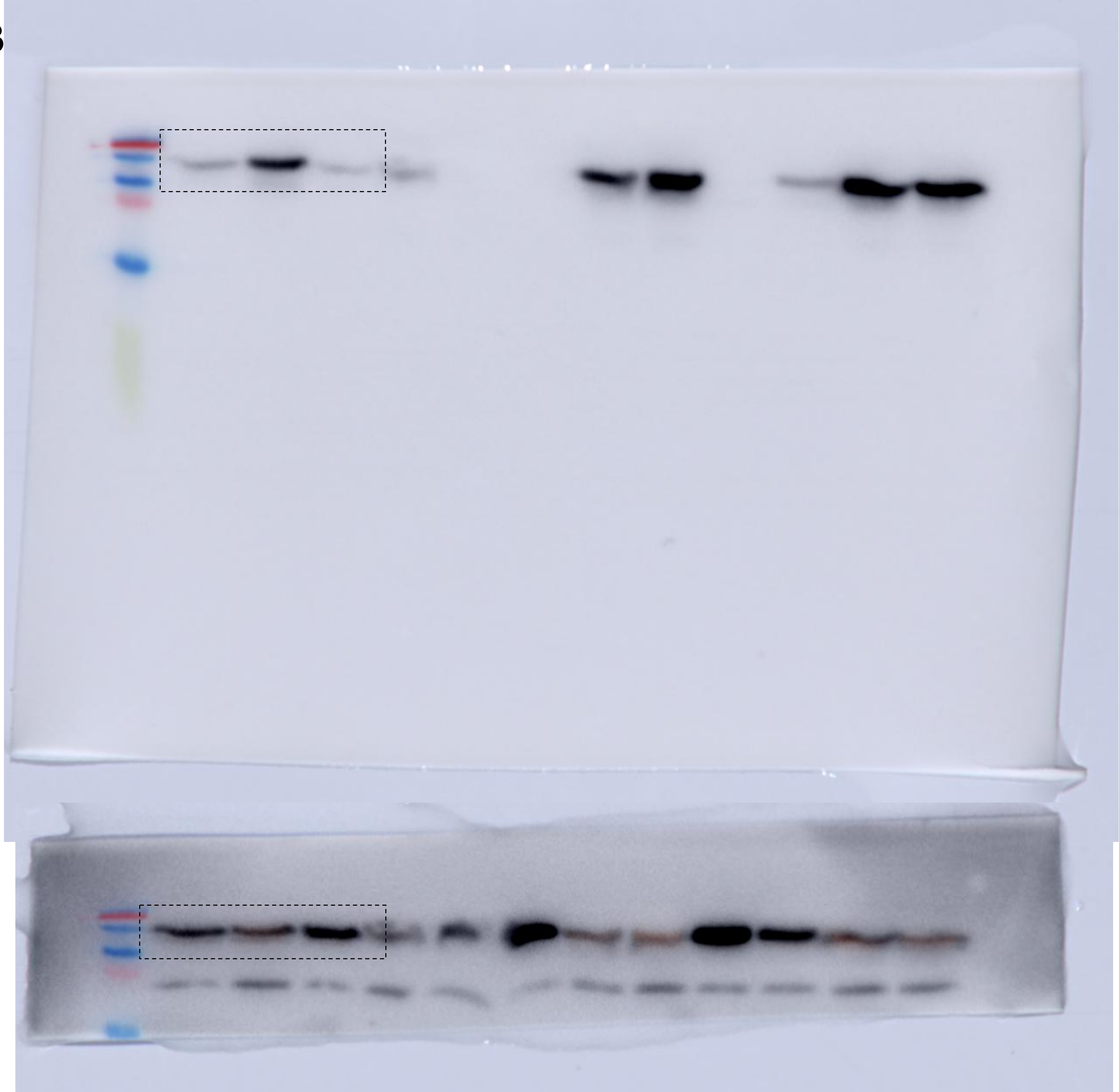

Figure 4C

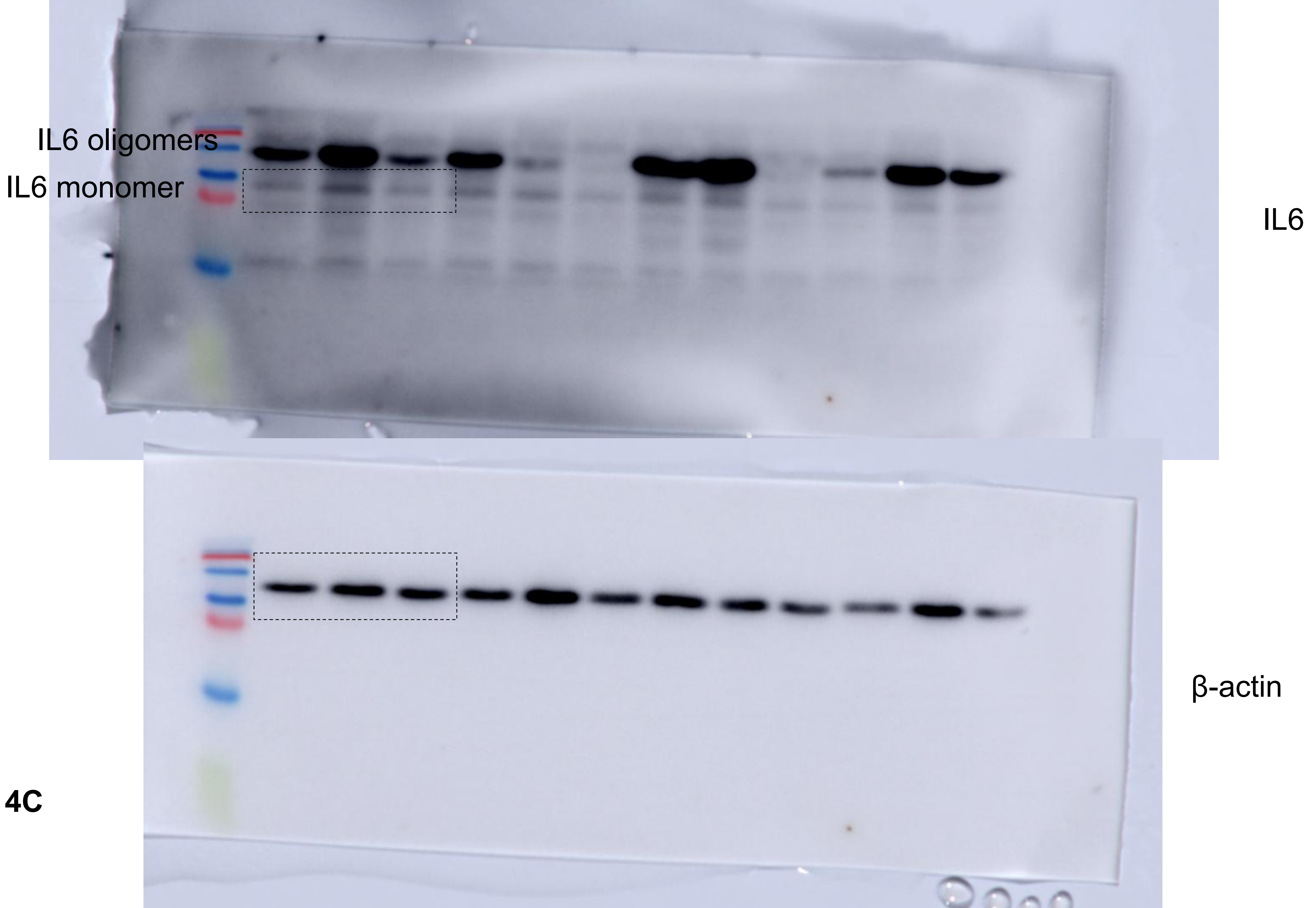

Figure 4C

Fibronectin

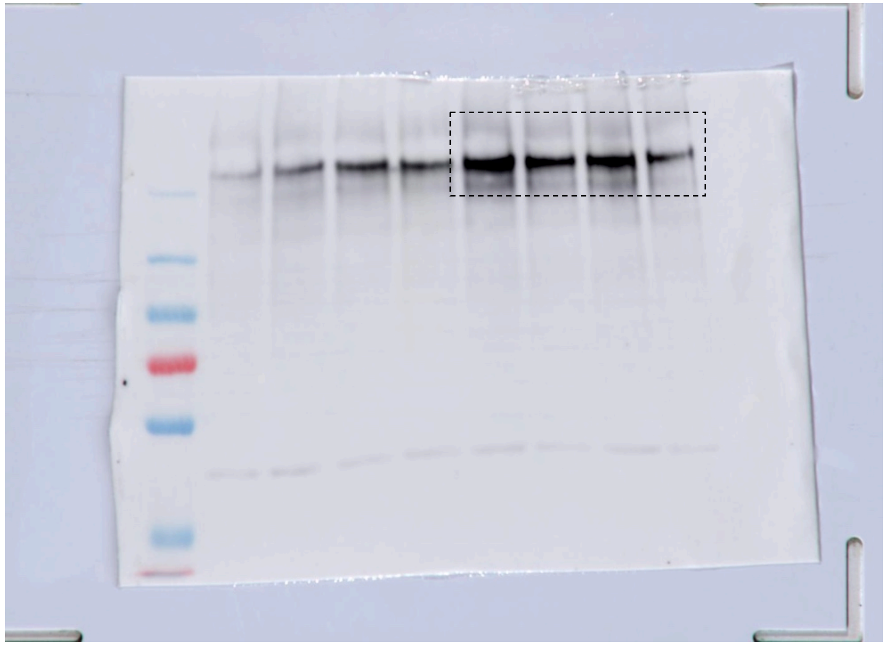

THBS-1

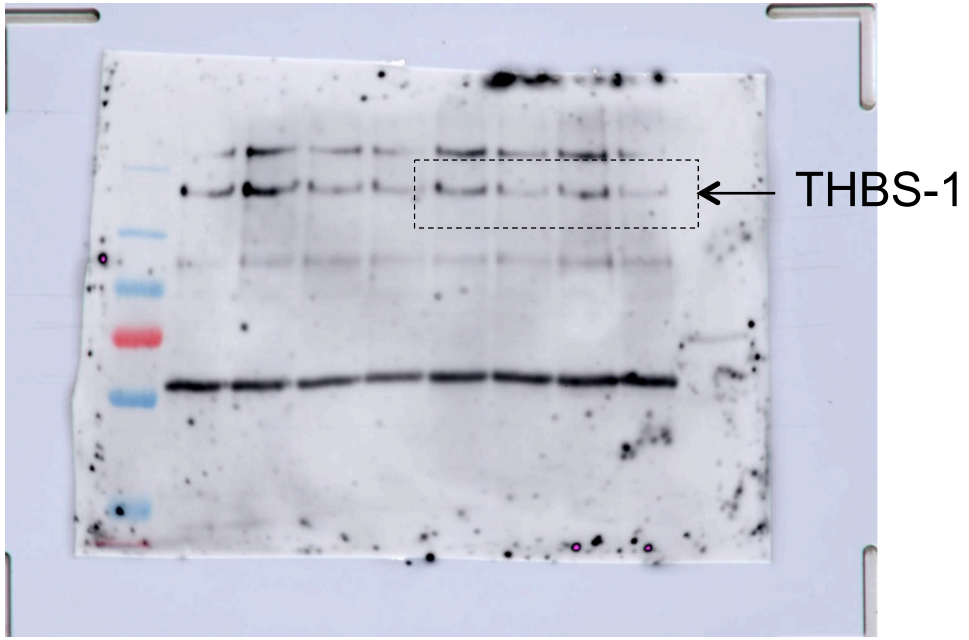

β-tubulin

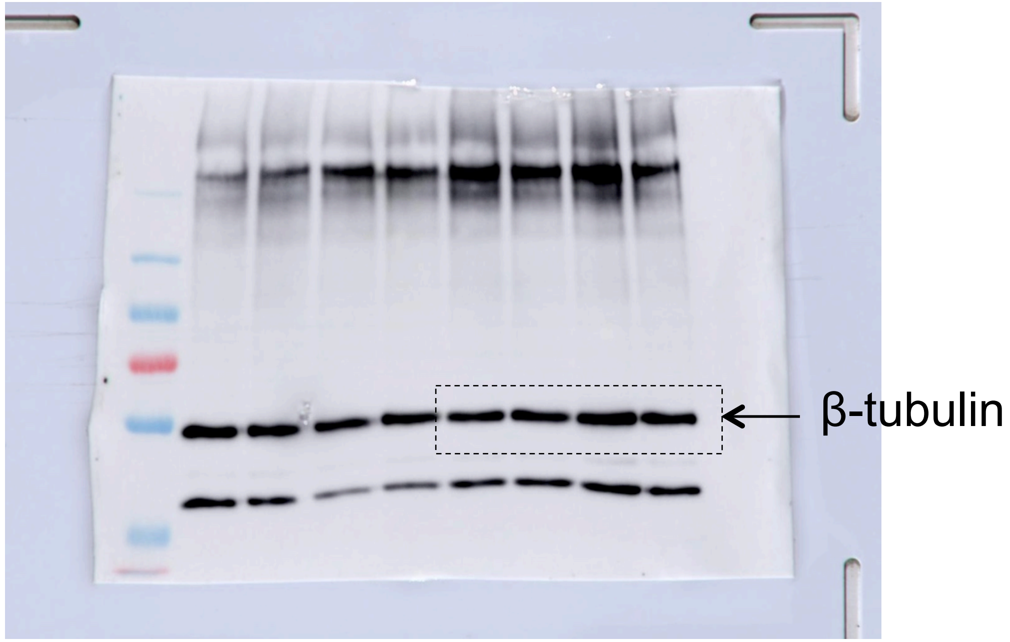

Figure 4F

pSMAD-2/3

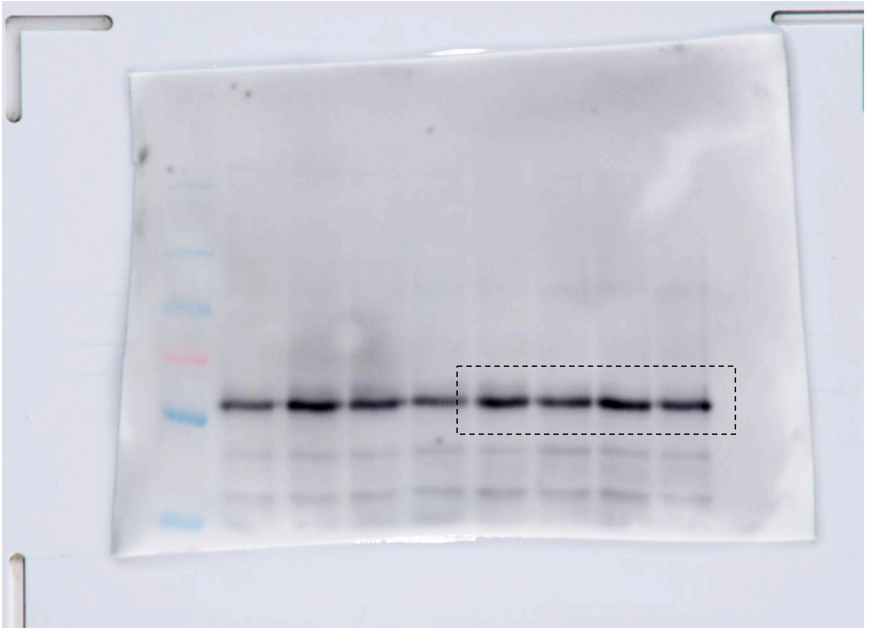

β-tubulin

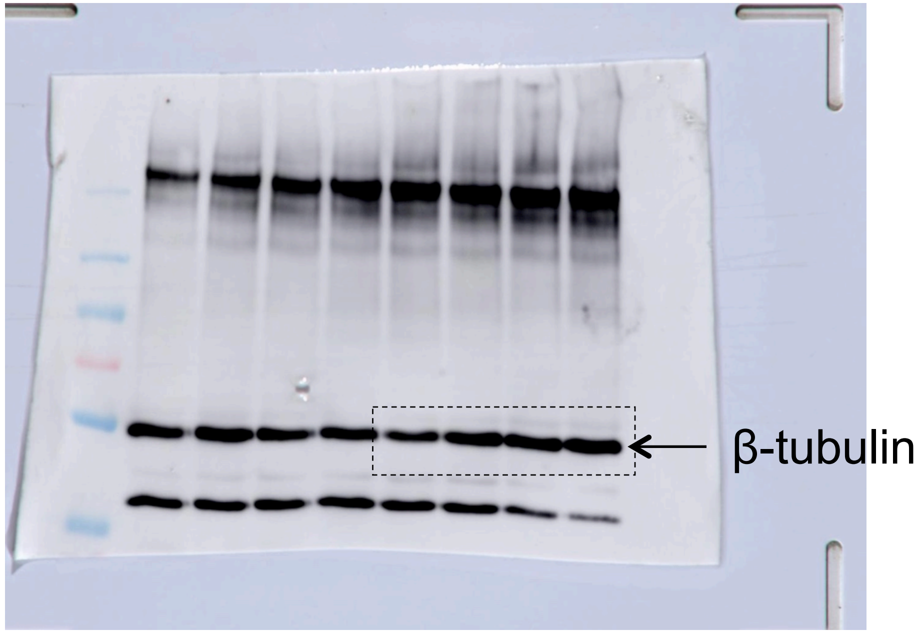

Figure 4F
