# Supplementary material for: Pro‐inflammatory immunity supports fibrosis advancement in epidermolysis bullosa: intervention with Ang‐(1‐7)
Source: EMBO Mol Med. 2021 Aug 30;13(10):e14392. doi: 10.15252/emmm.202114392 (PMC8495454; doi:10.15252/emmm.202114392)
Supplement: Supplementary file 13 — Source Data for Figure 7 [file EMMM-13-e14392-s010.pdf]

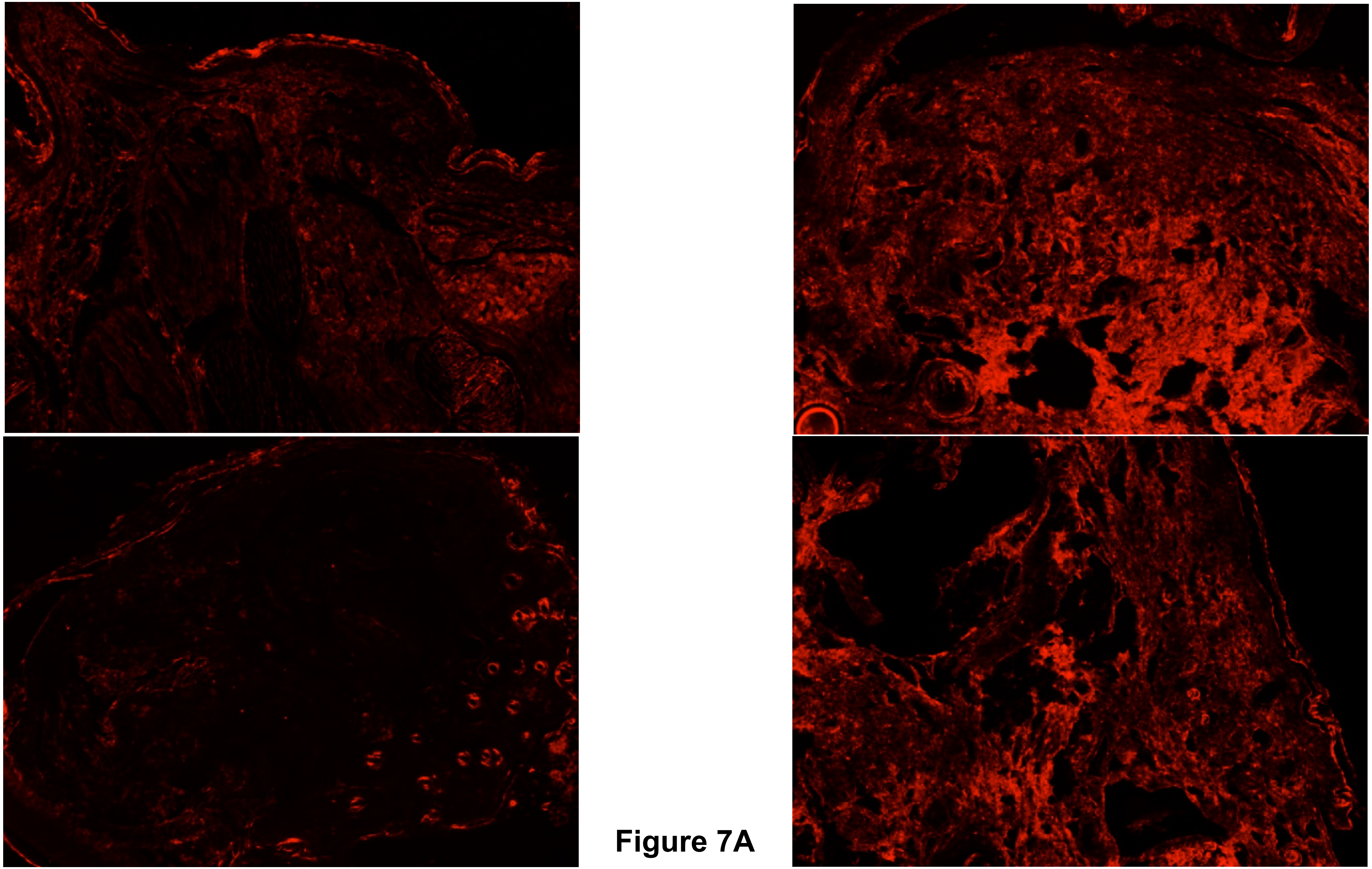

Figure 7A

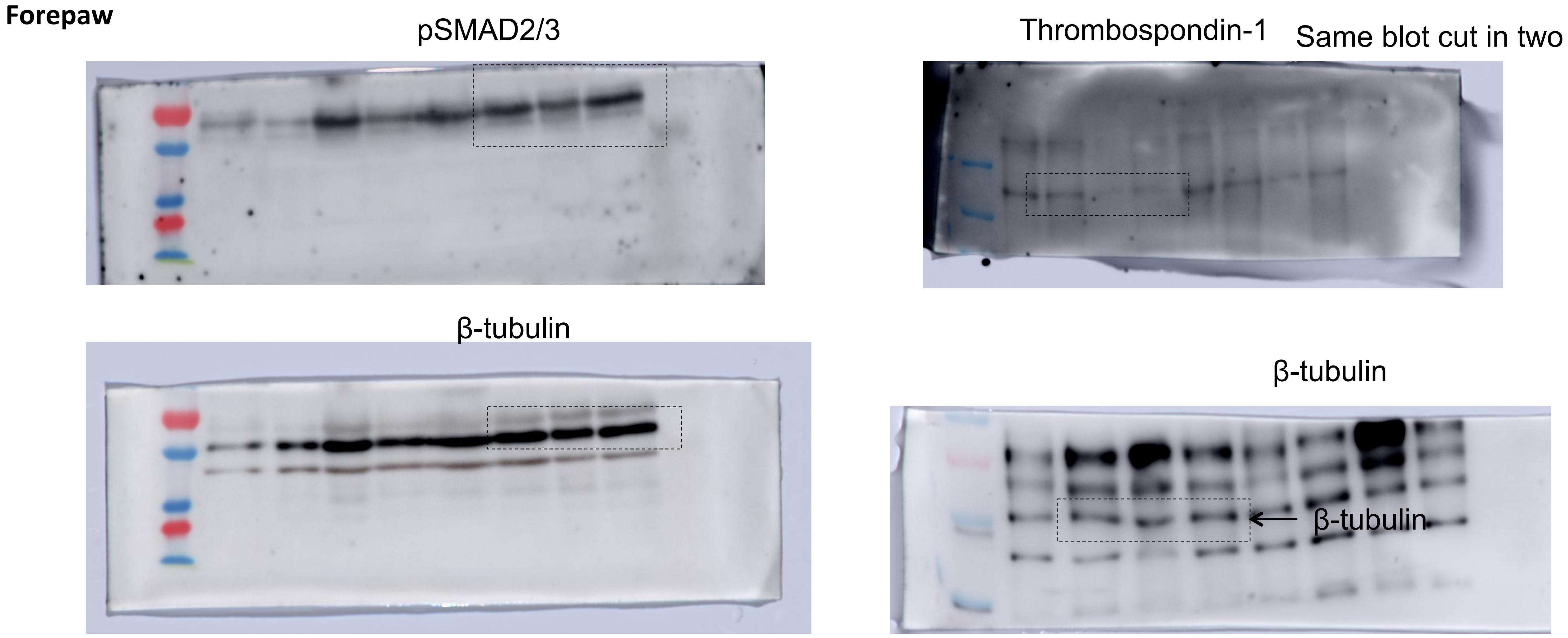

Forepaw
pSMAD2/3
Thrombospondin-1
Same blot cut in two
β-tubulin
β-tubulin
β-tubulin

Figure 7C

Forepaw

Same blot cut in two

Tenascin-C

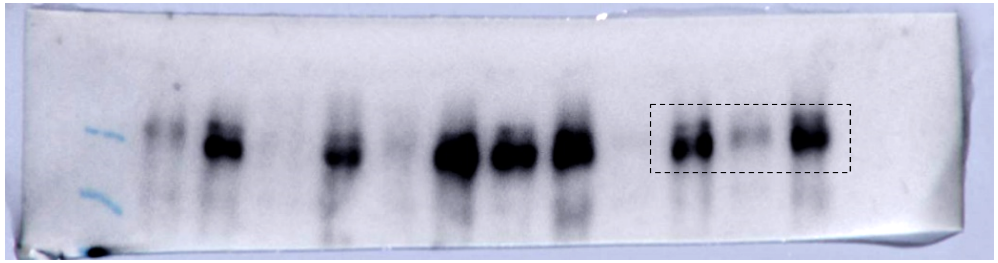

β-tubulin

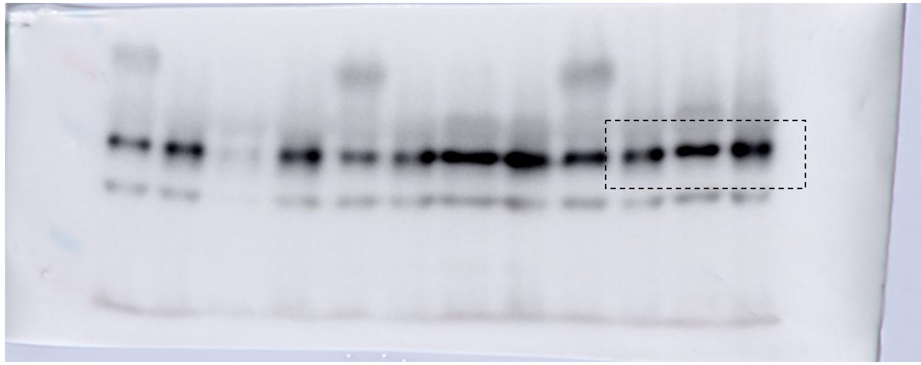

Fibronectin

Same blot cut in two

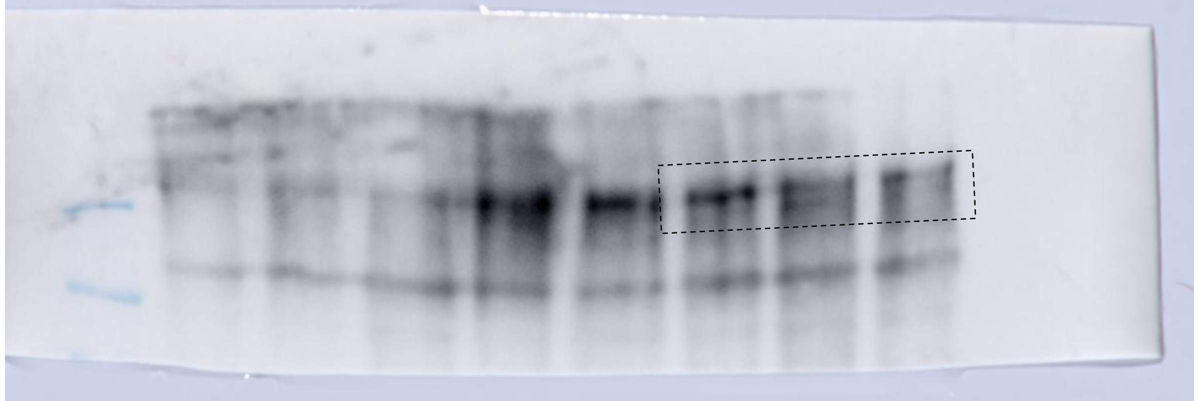

β-tubulin

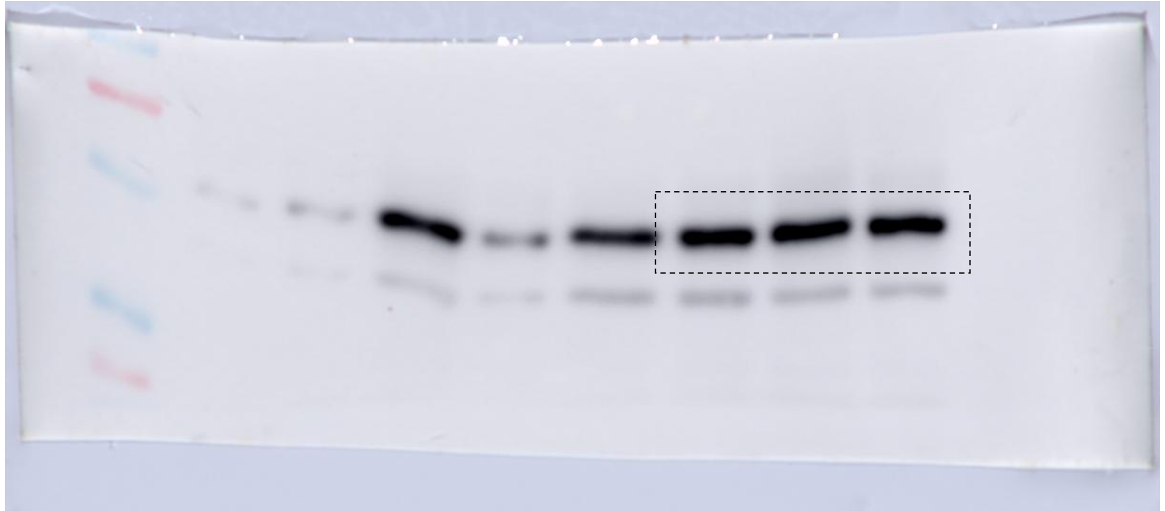

Figure 7C

Forepaw

β-arrestin-1/2

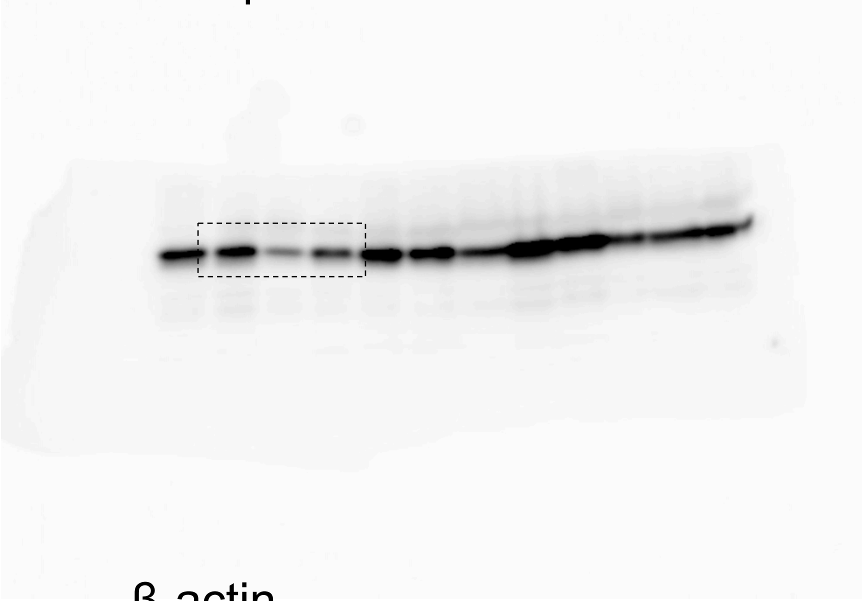

NF-kB

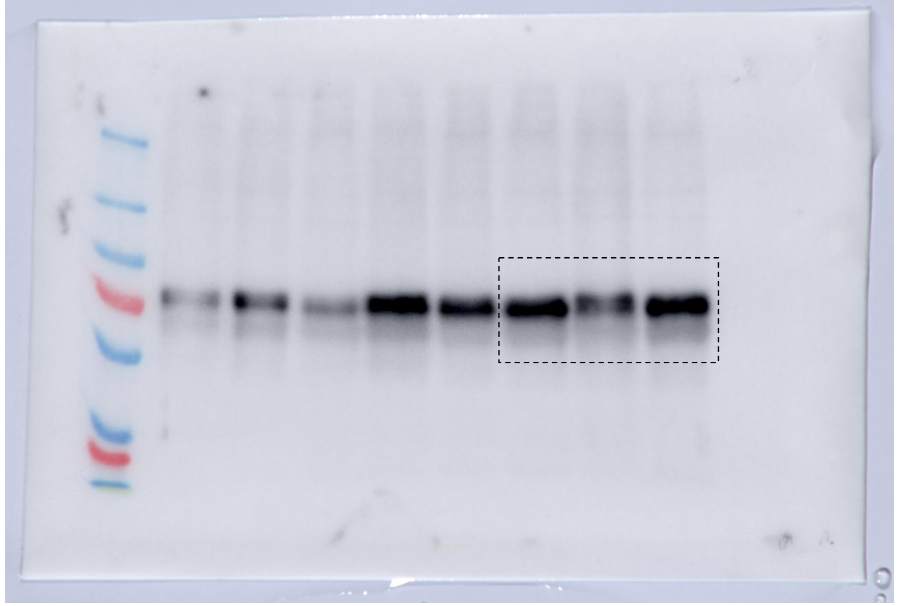

β-actin

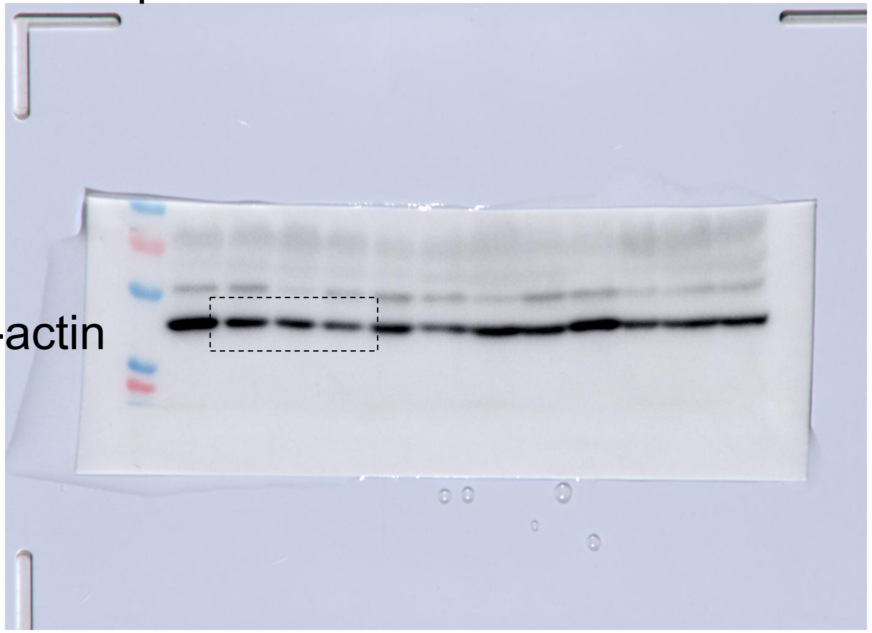

β-actin

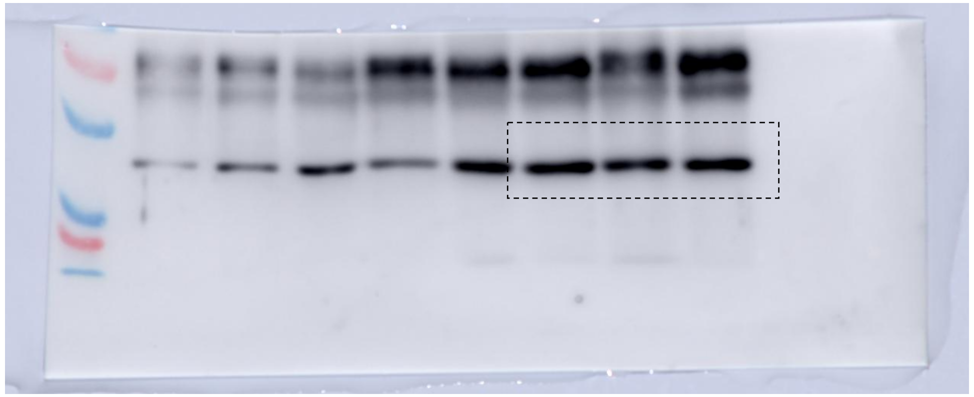

Figure 7C

β-arrestin-1/2

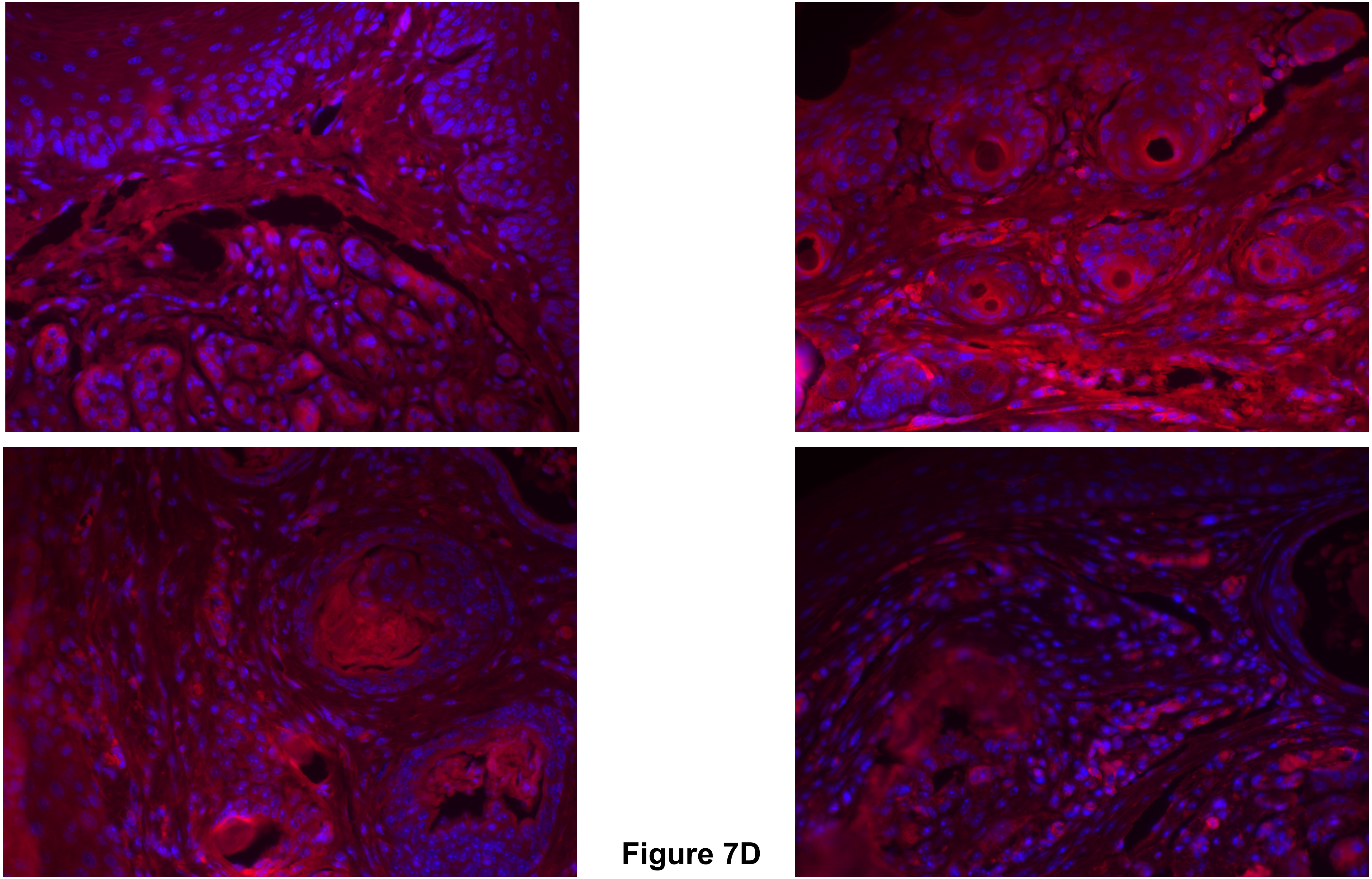

Figure 7D

NF-kB

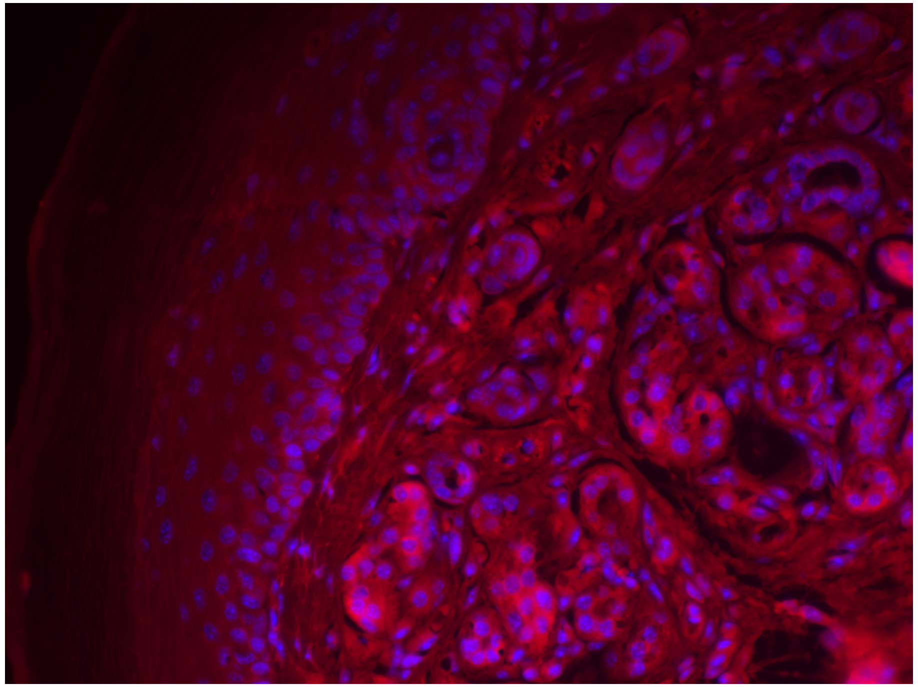

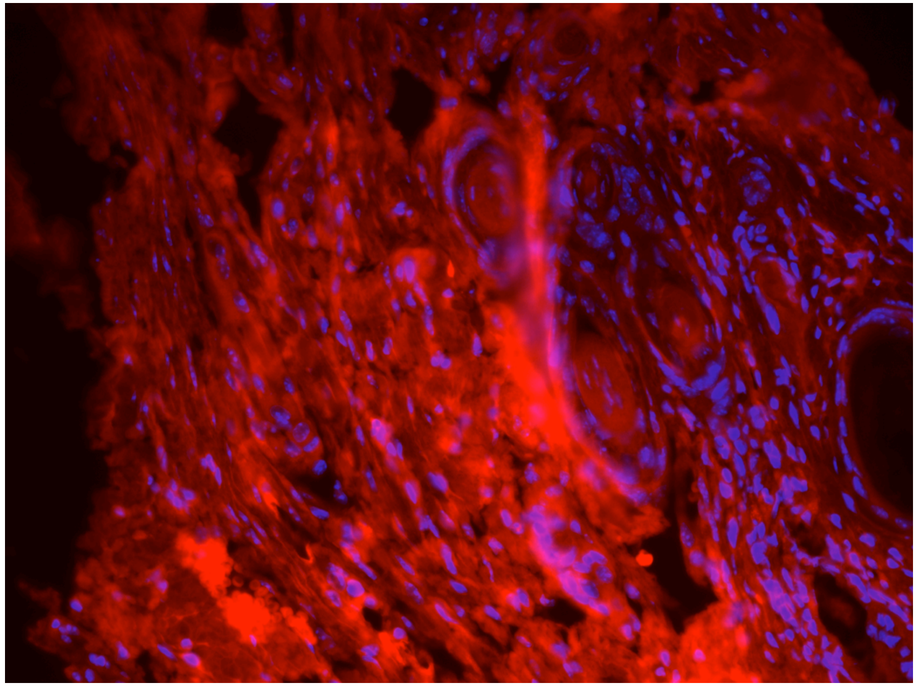

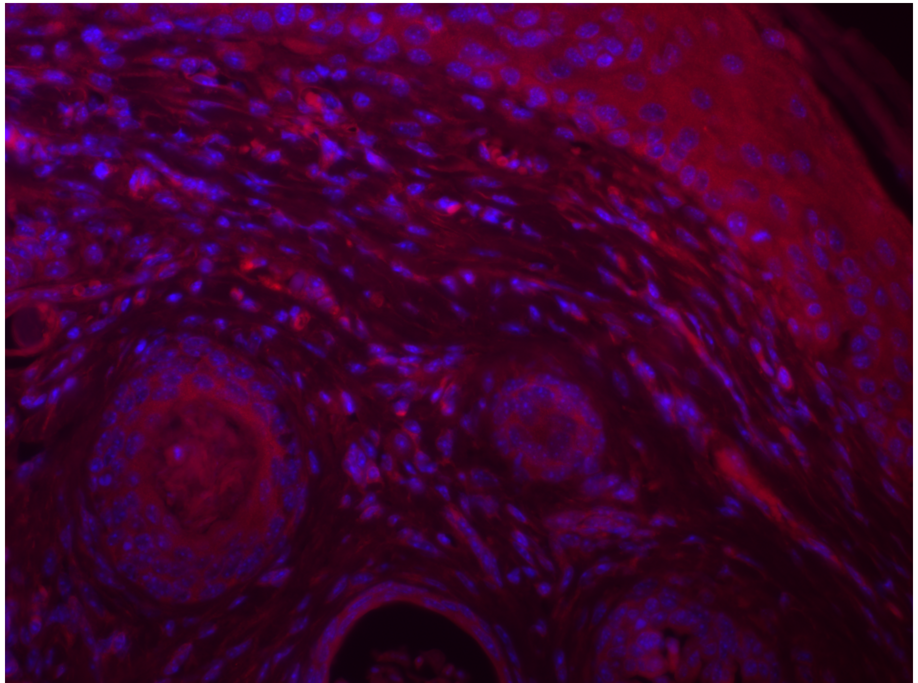

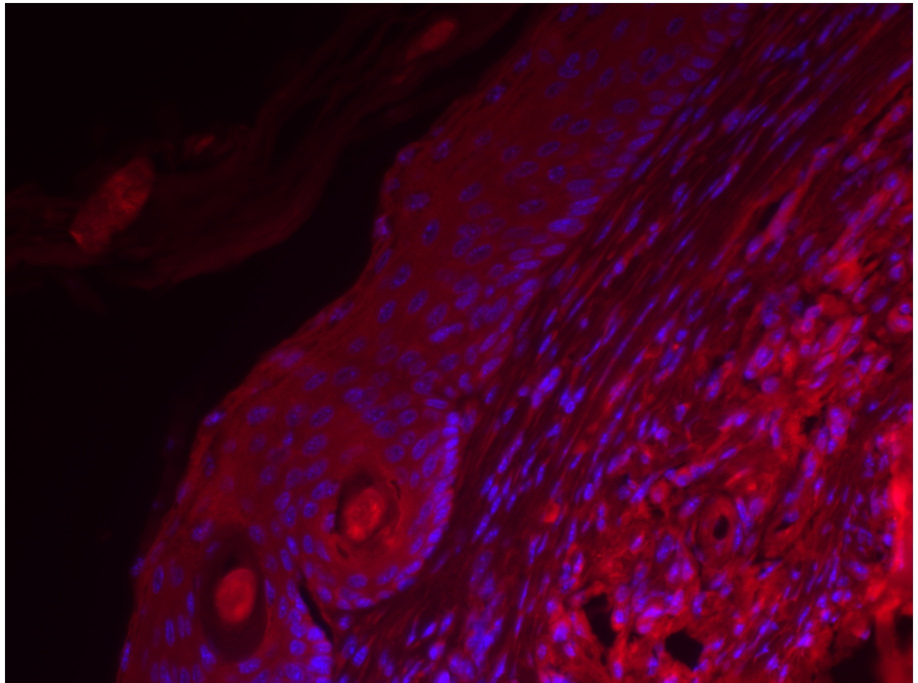

Figure 7D

Eye

Same blot cut in two

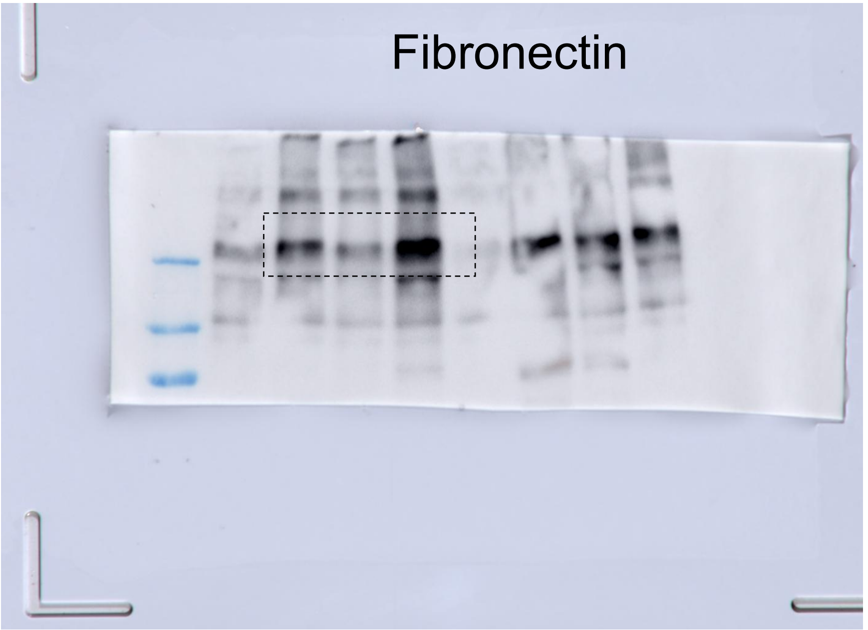

Tenascin-C

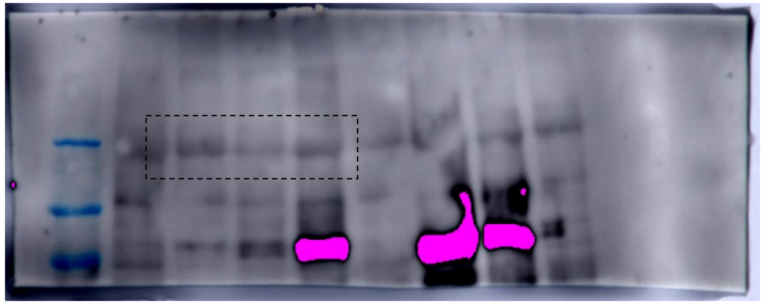

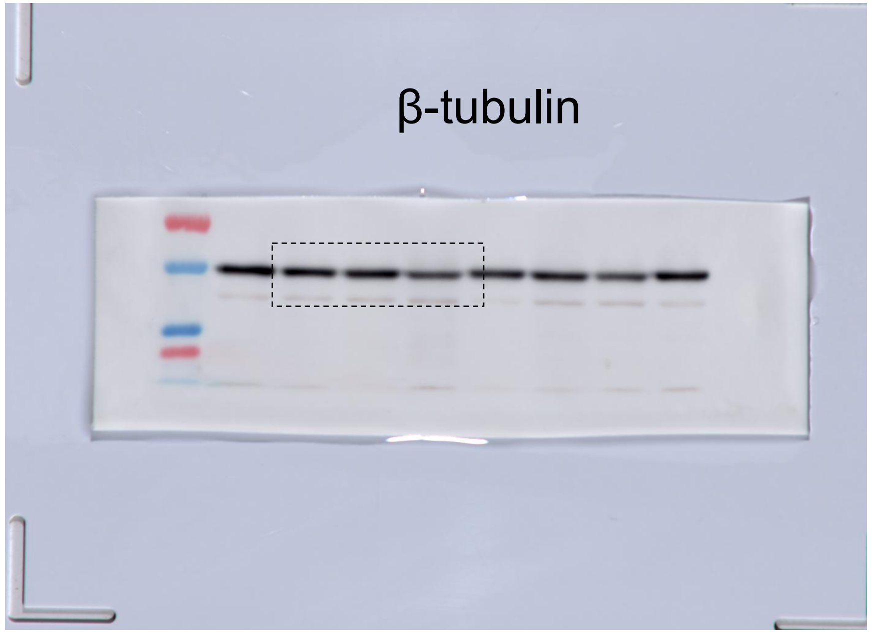

Figure 7E

Eye

α-SMA

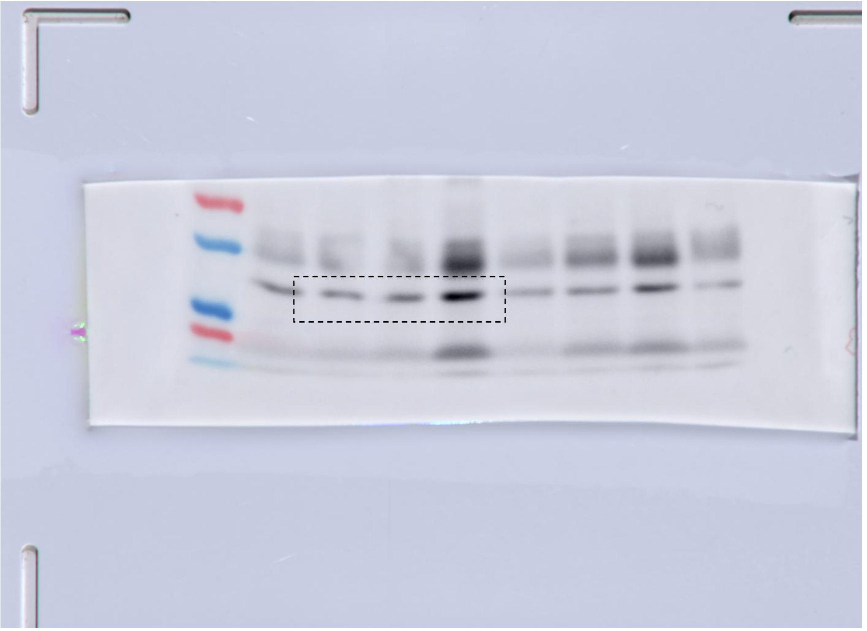

β-tubulin

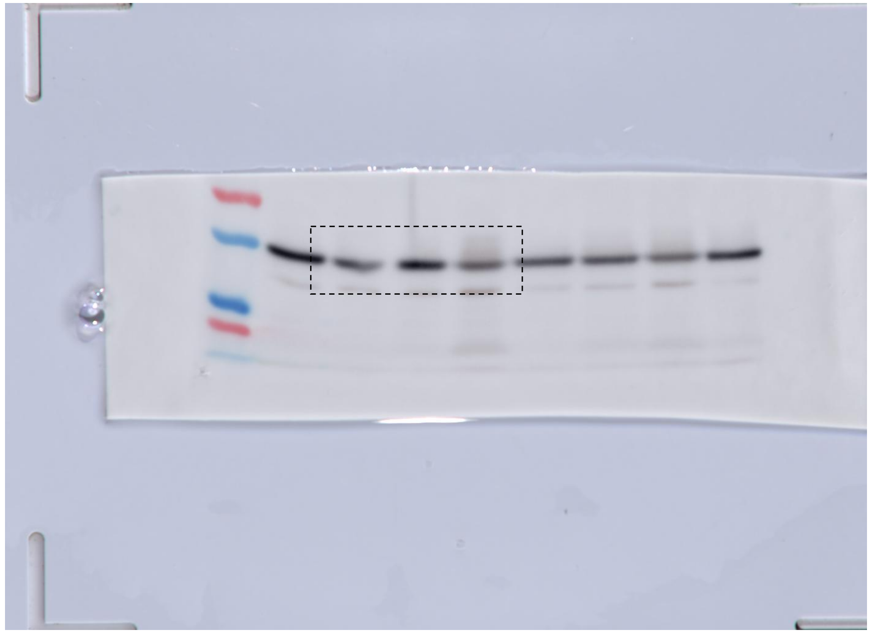

Figure 7E

Eye

Same blot cut in two

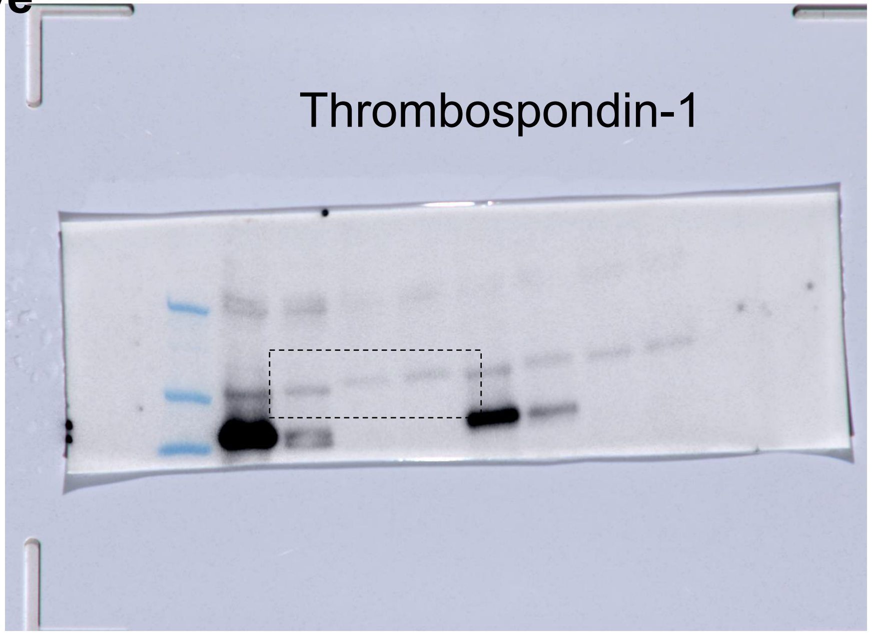

β-tubulin

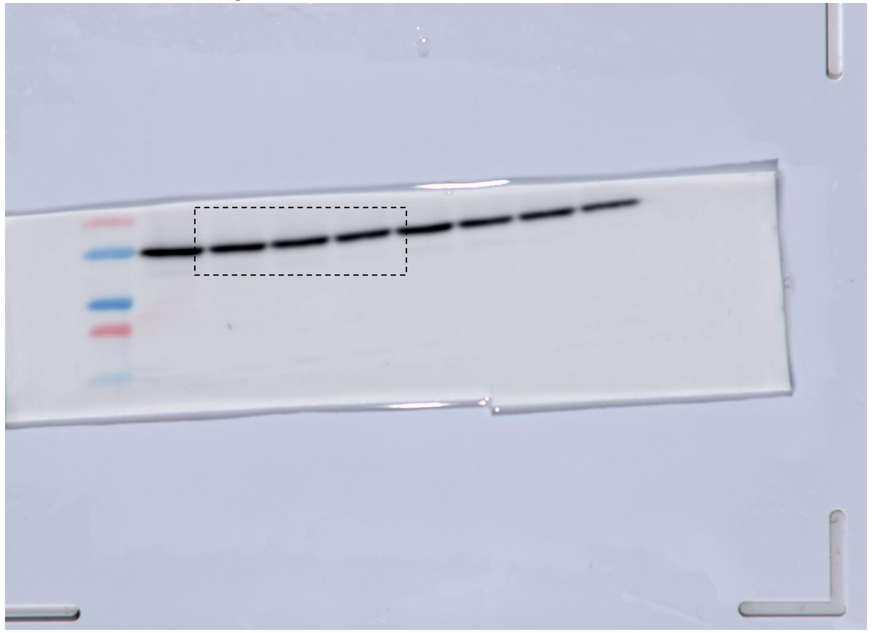

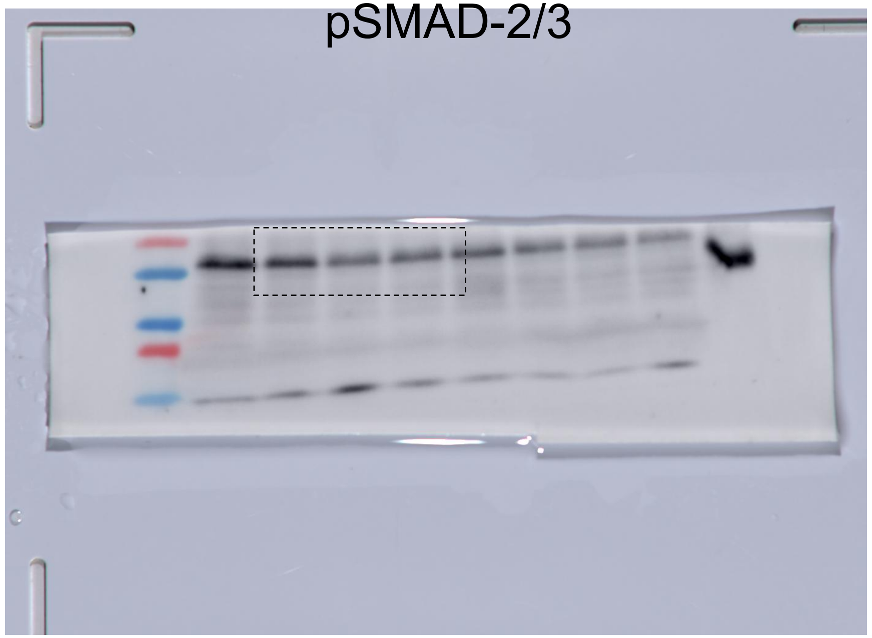

Figure 7E

Esophagus

Same blot cut in two

Fibronectin

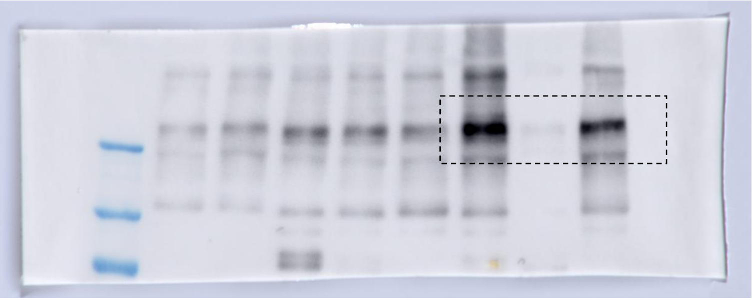

Tenascin-C

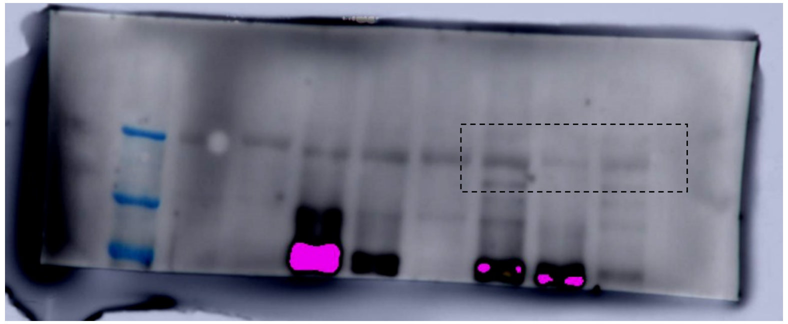

β-tubulin

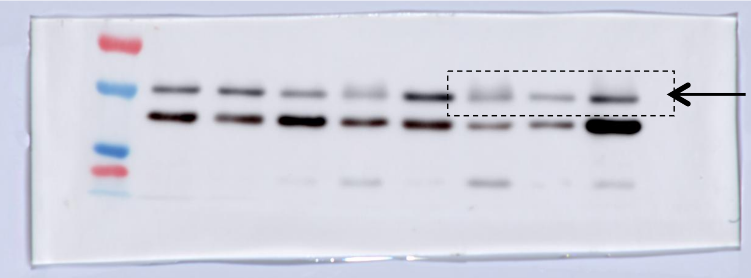

Figure 7E

Esophagus

pSMAD-2/3

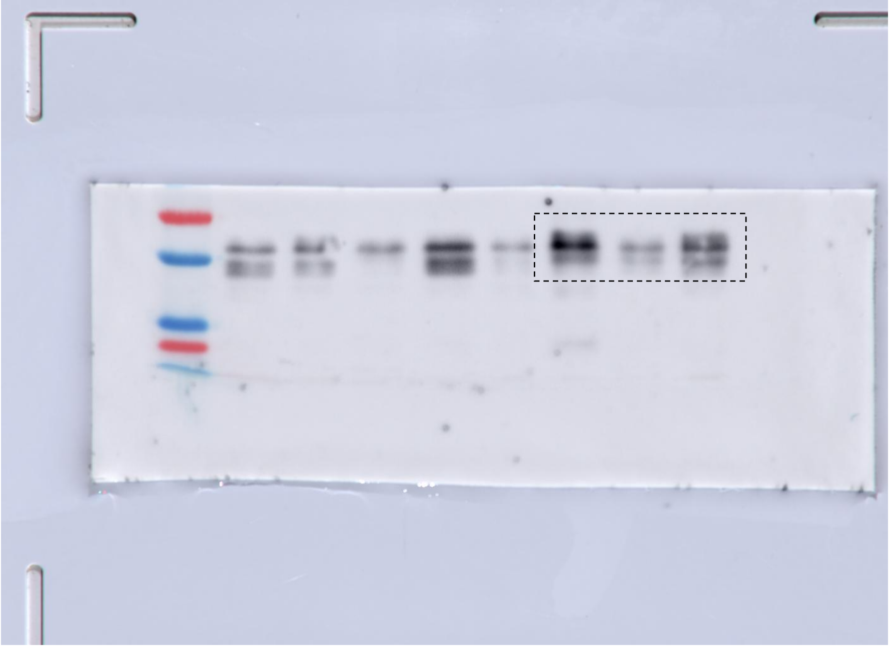

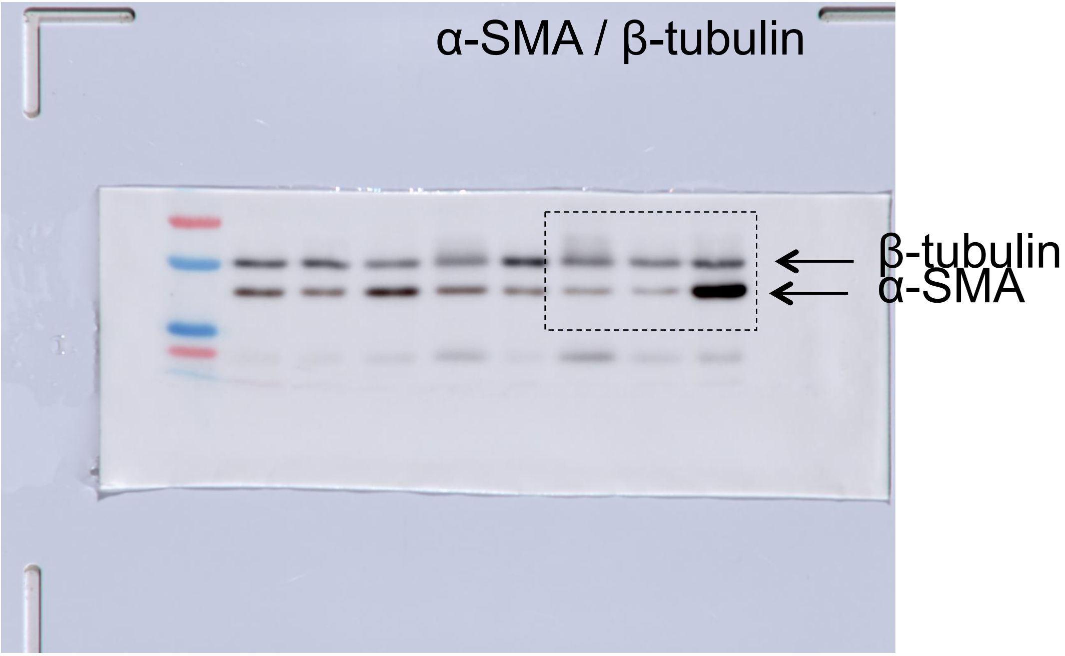

Figure 7E

Tongue

Fibronectin

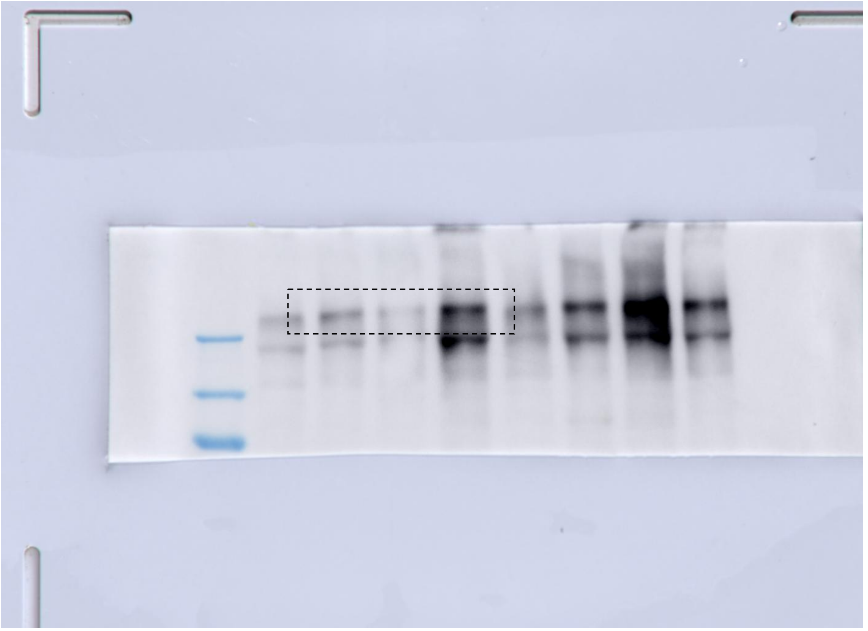

β-tubulin

Same blot cut in two

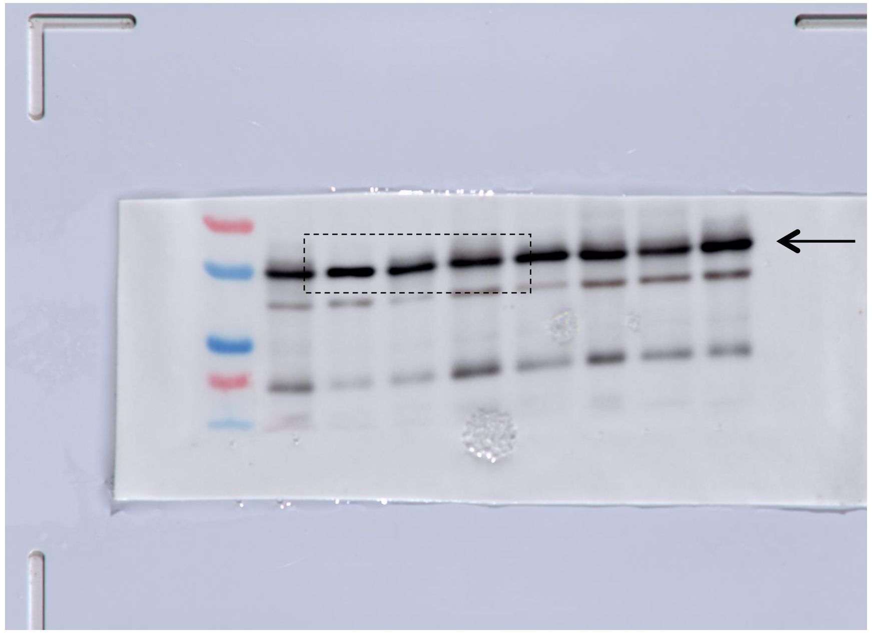

← β-tubulin

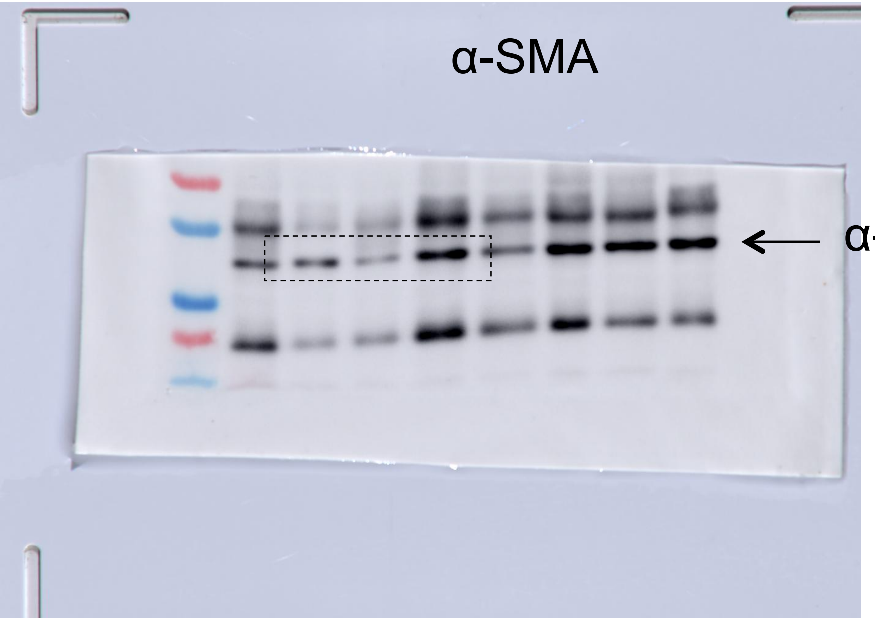

← α-SMA

Figure 7E

Tongue

Same blot cut in two

Tenascin-C

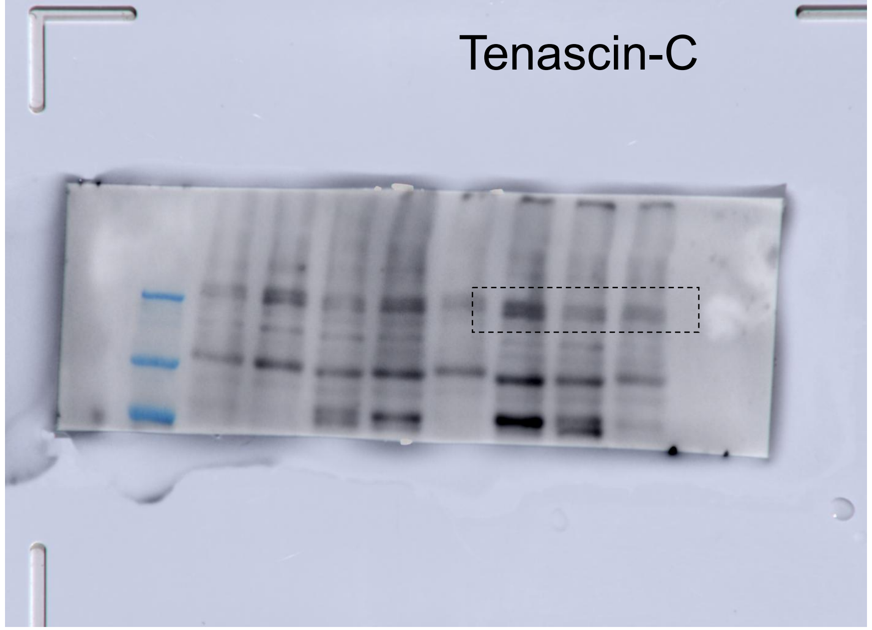

pSMAD2/3

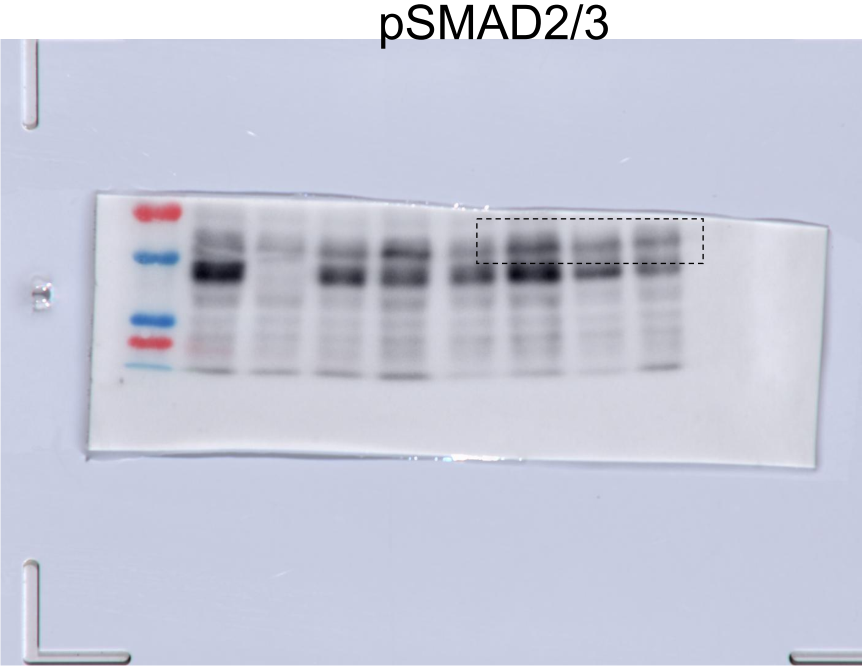

β-tubulin

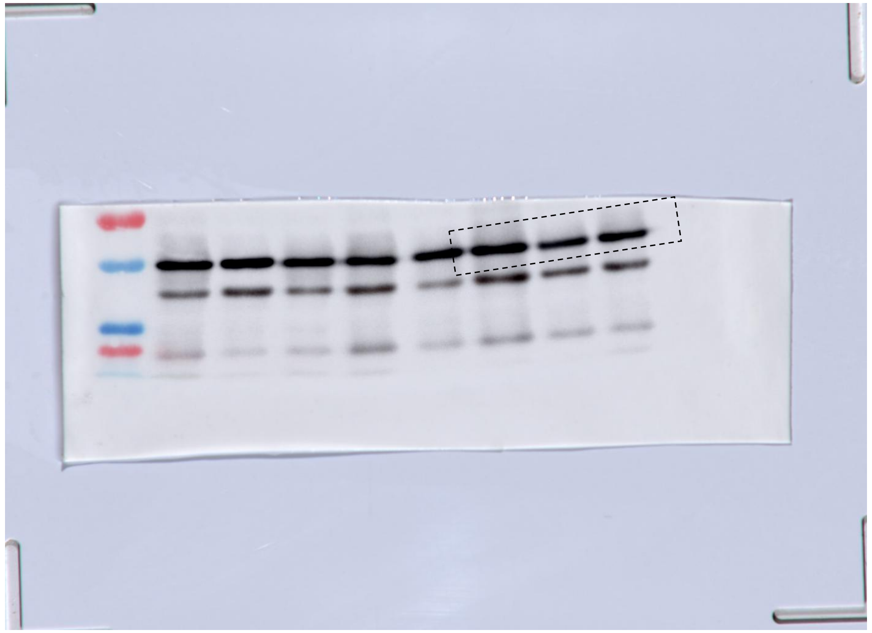

Figure 7E
