# Supplementary material for: Pro‐inflammatory immunity supports fibrosis advancement in epidermolysis bullosa: intervention with Ang‐(1‐7)
Source: EMBO Mol Med. 2021 Aug 30;13(10):e14392. doi: 10.15252/emmm.202114392 (PMC8495454; doi:10.15252/emmm.202114392)
Supplement: Supplementary file 14 [file EMMM-13-e14392-s002.pdf]

EMBO Molecular Medicine

Pro-inflammatory immunity supports fibrosis advancement in epidermolysis bullosa: intervention with Ang-(1-7)

Rocco Bernasconi, Kerstin Thriene, Elena Romero-Fernández, Christine Gretzmeier, Tobias Kühl, Mareike Maler, Pauline Nauroy, Svenja Kleiser, Anne-Catherine Rühl-Muth, Michael Stumpe, Dimitra Kiritsi, Stefan Martin, Boris Hinz, Leena Bruckner-Tuderman, Jörn Dengjel, and Alexander Nyström
DOI: 10.15252/emmm.202114392

Corresponding author: Alexander Nyström (alexander.nystroem@uniklinik-freiburg.de)

Review Timeline:

Submission Date:	7th Apr 21
Editorial Decision:	29th Apr 21
Revision Received:	24th Jun 21
Editorial Decision:	15th Jul 21
Revision Received:	19th Jul 21
Accepted:	23rd Jul 21

Editor: Lise Roth

Transaction Report:

(Note: With the exception of the correction of typographical or spelling errors that could be a source of ambiguity, letters and reports are not edited. Depending on transfer agreements, referee reports obtained elsewhere may or may not be included in this compilation. Referee reports are anonymous unless the Referee chooses to sign their reports.)

1st Editorial Decision 29th Apr 2021

29th Apr 2021

Dear Dr. Nyström,

Thank you for the submission of your manuscript to EMBO Molecular Medicine. We have now received feedback from the three reviewers who agreed to evaluate your manuscript. As you will see from the reports below, the referees acknowledge the interest of the study and are overall supporting publication of your work pending appropriate revisions.

Addressing the reviewers' concerns in full will be necessary for further considering the manuscript in our journal, and acceptance of the manuscript will entail a second round of review. EMBO Molecular Medicine encourages a single round of revision only and therefore, acceptance or rejection of the manuscript will depend on the completeness of your responses included in the next, final version of the manuscript. For this reason, and to save you from any frustrations in the end, I would strongly advise against returning an incomplete revision.

***

When submitting your revised manuscript, please carefully review the instructions that follow below. Failure to include requested items will delay the evaluation of your revision:

1) A .docx formatted version of the manuscript text (including legends for main figures, EV figures and tables). Please make sure that the changes are highlighted to be clearly visible.

2) Individual production quality figure files as .eps, .tif, .jpg (one file per figure).

3) A .docx formatted letter INCLUDING the reviewers' reports and your detailed point-by-point responses to their comments. As part of the EMBO Press transparent editorial process, the point-by-point response is part of the Review Process File (RPF), which will be published alongside your paper.

4) A complete author checklist, which you can download from our author guidelines (https://www.embopress.org/page/journal/17574684/authorguide#submissionofrevisions). Please insert information in the checklist that is also reflected in the manuscript. The completed author checklist will also be part of the RPF.

5) Before submitting your revision, primary datasets produced in this study need to be deposited in an appropriate public database (see https://www.embopress.org/page/journal/17574684/authorguide#dataavailability).
Please remember to provide a reviewer password if the datasets are not yet public.
The accession numbers and database should be listed in a formal "Data Availability " section (placed after Materials & Method). Please note that the Data Availability Section is restricted to new primary data that are part of this study.

6) We would also encourage you to include the source data for figure panels that show essential data. Numerical data should be provided as individual .xls or .csv files (including a tab describing the

data). For blots or microscopy, uncropped images should be submitted (using a zip archive if multiple images need to be supplied for one panel). Additional information on source data and instruction on how to label the files are available at
.

7) Our journal encourages inclusion of *data citations in the reference list* to directly cite datasets that were re-used and obtained from public databases. Data citations in the article text are distinct from normal bibliographical citations and should directly link to the database records from which the data can be accessed. In the main text, data citations are formatted as follows: "Data ref: Smith et al, 2001" or "Data ref: NCBI Sequence Read Archive PRJNA342805, 2017". In the Reference list, data citations must be labeled with "[DATASET]". A data reference must provide the database name, accession number/identifiers and a resolvable link to the landing page from which the data can be accessed at the end of the reference. Further instructions are available at .

8) We replaced Supplementary Information with Expanded View (EV) Figures and Tables that are collapsible/expandable online. A maximum of 5 EV Figures can be typeset. EV Figures should be cited as 'Figure EV1, Figure EV2" etc... in the text and their respective legends should be included in the main text after the legends of regular figures.

- For the figures that you do NOT wish to display as Expanded View figures, they should be bundled together with their legends in a single PDF file called *Appendix*, which should start with a short Table of Content. Appendix figures should be referred to in the main text as: "Appendix Figure S1, Appendix Figure S2" etc.

- Additional Tables/Datasets should be labeled and referred to as Table EV1, Dataset EV1, etc. Legends have to be provided in a separate tab in case of .xls files. Alternatively, the legend can be supplied as a separate text file (README) and zipped together with the Table/Dataset file.
See detailed instructions here:
.

9) For more information: There is space at the end of each article to list relevant web links for further consultation by our readers. Could you identify some relevant ones and provide such information as well? Some examples are patient associations, relevant databases, OMIM/proteins/genes links, author's websites, etc...

10) Every published paper now includes a 'Synopsis' to further enhance discoverability. Synopses are displayed on the journal webpage and are freely accessible to all readers. They include a short stand first (maximum of 300 characters, including space) as well as 2-5 one-sentences bullet points that summarizes the paper. Please write the bullet points to summarize the key NEW findings. They should be designed to be complementary to the abstract - i.e. not repeat the same text. We encourage inclusion of key acronyms and quantitative information (maximum of 30 words / bullet point). Please use the passive voice. Please attach these in a separate file or send them by email, we will incorporate them accordingly.

Please also suggest a striking image or visual abstract to illustrate your article. If you do please provide a png file 550 px-wide x 400-px high.

11) As part of the EMBO Publications transparent editorial process initiative (see our Editorial at http://embomolmed.embopress.org/content/2/9/329), EMBO Molecular Medicine will publish online a Review Process File (RPF) to accompany accepted manuscripts.

In the event of acceptance, this file will be published in conjunction with your paper and will include the anonymous referee reports, your point-by-point response and all pertinent correspondence relating to the manuscript. Let us know whether you agree with the publication of the RPF and as here, if you want to remove or not any figures from it prior to publication.
Please note that the Authors checklist will be published at the end of the RPF.

EMBO Molecular Medicine has a "scooping protection" policy, whereby similar findings that are published by others during review or revision are not a criterion for rejection. Should you decide to submit a revised version, I do ask that you get in touch after three months if you have not completed it, to update us on the status.

I look forward to receiving your revised manuscript.

Yours sincerely,

Lise Roth

Lise Roth, PhD
Editor
EMBO Molecular Medicine

To submit your manuscript, please follow this link:

Link Not Available

*Additional important information regarding Figures

Each figure should be given in a separate file and should have the following resolution:
Graphs 800-1,200 DPI
Photos 400-800 DPI
Colour (only CMYK) 300-400 DPI"

Figures are not edited by the production team. All lettering should be the same size and style; figure panels should be indicated by capital letters (A, B, C etc). Gridlines are not allowed except for log plots. Figures should be numbered in the order of their appearance in the text with Arabic numerals. Each Figure must have a separate legend and a caption is needed for each panel.

*Additional important information regarding figures and illustrations can be found at https://bit.ly/EMBOPressFigurePreparationGuideline

***** Reviewer's comments *****

Referee #1 (Remarks for Author):

Bernasconi et al's manuscript entitled "Pro-inflammatory immunity drives fibrosis in EB: intervention with Ang-(1-7)" uses both a mouse model and human RDEB cells and tissue to characterise the proteome of progressive RDEB associated fibrosis, to characterise local and systemic inflammation associated with disease progression, and to test the effects of Ang-(1-7) on in vitro cell cultures and in vivo disease.
The Introduction provides a nicely-written background to the problem, and key information needed to understand the rationale to the current work. Bottom of page 3, please declare more about the historic proteomic analyses of the RDEB mice, and mid-Page 4, provide the existing background about inflammation in RDEB. Also bottom of Pg3, in reference to the previous TGFb findings, please include that this information relates to work in the mouse model.
RESULTS - The longitudinal analysis of the tissue proteome somewhat builds upon the authors' 2015 paper. Perhaps reassuringly the conclusions are the same (inflammation is abundant) but the incremental nature of this should be declared in Intro and Discussion. The proteomic data in the supplementary files need improved column headings/annotations (e.g. what are the FC columns - state numerator/denominator and somewhere explain that H are the hypomorphs).
The mechanical testing at a single site (i.e. not the back) limits the ability to include the friction-stiffness association in the interpretations in the first paragraph on Pg7.
Other studies have previously revealed that the protein composition of fibrotic lesions may not include more Collagen I. With the importance placed on this observation in the abstract and final discussion paragraph, please relate it to the literature, and if space allows, discuss any possible limitations of this particular proteomic approach (e.g. extraction method, solubility) that may be influencing this observation.
The inflammation section of the manuscript is clear (pg8-10), well supported by figure 2. However, contextualisation is lacking (e.g. identifying the existing knowledge and gaps in the intro, as well as discussion about how this fits in, for example, with neutrophil targeting in other EB types). The extrapolation to human tissue is a nice addition to this study, although the data largely confirms what is known (inflammatory infiltrate in RDEB skin), and as mentioned previously, it is important to cite these other studies.
P10. "kininogen appeared unique" (more explanation required; unique to RDEB? Unique to forepaw in the 0 and 4 week time-points?) Top of P11 - correct the text referring to Fig S7 - this figure does not address Kininogen function. This paragraph requires some clarification.
2nd half Pg12 - the use of the word "protracted" in the sub-title and text implies that the time-course (of in vitro signalling) was investigated - but 4b is just a single time-point. Its later use (e.g. at the end of the section) implies that 2 days of co-culture is long (protracted), and further suggests that the authors think the response might be delayed rather than just dampened. The use of this word throughout should be reconsidered. Top of Pg13, please state the half-life from this reference. I see that it is likely less than an hour, but the possibility of it triggering a cell differentiation/polarisation event that persists feels distinct from a "protracted" effect.
In the co-culture experiments, one gap seems to be any kind of molecular detail about how the Thp-1 cells are changing in response to the Ang-(1-7) - and indeed how they are first "activated" by the collagen. As much as there is a reference here, it will be important to show a sign of activation triggered by the collagen as stated. And although complete characterisation of the dampening effects of Ang-(1-7) on the pro-fibrotic actions of the Thp-1 cells may be beyond the scope of this manuscript, it is thought to be important to show "something" is altered by this treatment in the in vitro system. Maybe MHC II plus a few key pro-inflammatory cytokines?
Here, Thrombospondin-1 (and Fn and pSmad2/3 and gel contraction) are used as read-outs of fibrosis, but Thrombospondin-1 is not apparent in the proteomic dataset (S1). Also, does the b-arrestin data from the proteomics support the narrative?

Bottom of Pg13, please add a few additional details to re-explain the receptor-inhibitor pairs, as well as your interpretation of the blots in S11 (their current quality is insufficient for the reader to see - so their improvement would help).
The in vivo paw measurements and improvements across all organs (Figs 5-7) following low-dose Ang-(1-7) are compelling and of wide interest. That said, in Figure 5B, the statistics are questionable. The detrimental effects of the high dose appear significant, and this will be an important outcome from this work. Also in 5A, is the histological section folded for the low-dose image? It looks strange and distracting enough that it is difficult to comment on cellularity, for example.
Discussion - bottom of pg 17, specify "genetic mouse model". The "quenched inflammatory activity" (pg19) feels exaggerated, again given only MHC II data (gentler language is needed).
To conclude, this is a very broad study containing useful datasets, but the proteomic findings closely replicate the findings of Nystrom et al 2015. There is also some concern about the mechanistically superficial nature of the anti-inflammatory actions of Ang-(1-7).
Having been through the paper, revisiting the title and abstract reveals over-statements. The title should be adjusted since this work does not demonstrate the pro-inflammatory immunity drives fibrosis. In the abstract, many adjustments are needed: it currently states a "discovery" of pro-inflammatory immunity associating with fibrosis, although this has been described (perhaps not by proteomics) by many, including these authors in 2015. Similarly, this study is not the first to find that fibrosis is more complex than too much of the most common fibrillar proteins. I agree that targeting inflammatory cell-fibroblast communication seems strategic, but it is not clear that this was the mechanism of action of the Ang-(1-7) here, particularly in the in vivo work in Figures 5-7. The in vivo results in the abstract include an alleviation of inflammation/down-modulation of pro-inflammatory immunity, but that was only based on MHC II and indeed alternative mechanisms could be at play. The second-to-last sentence does not belong in this abstract, without conducting the comparative analysis yourselves (this type of work is indeed being undertaken across the research community).

Referee #2 (Remarks for Author):

The data presented in this manuscript "Pro-inflammatory immunity drives fibrosis in epidermolysis bullosa: intervention with Ang-(1-7)" conclusively shows how changes in the ECM organization and inflammation are associated with fibrosis progression in RDEB mice and human RDEB skin samples. The conceptual approach that led to the investigation of Ang-(1-7) as therapeutic agent for RDEB is plausible and the meta-analyses comprising both, human and animal data, are included appropriately. Aside from technical comments and some missing supplementary data (stated below), this paper is well written and of substantial interest for future treatment of RDEB.
Comments:
1. Fig. 1D: The analyses of fuzzy c-means algorithm are not clearly addressed. Please describe in detail the protein expression profiles of each cluster: which clusters represent proteins that are upregulated or downregulated, whereas which clusters represent proteins displaying a bi-model expression pattern.
Besides, what does the y-axis label "fold change" mean? Please define it in the figure legend.
2. Fig. 2: Please provide the representative images of FACS analyses of neutrophils, macrophages and MHC II.
3. In Fig. 2F the y-axis label of monocyte markers is erroneously indicated. The label "% Ly6G- CD45+ cells" is used for neutrophils, not for monocytes. Please correct it accordingly.
4. Fig. 4A: Please provide the quantification of western blot analysis of individual proteins in RDEBF cell lysate.

5. Fig. 4B: Please include a bar graph of an untreated control group in Fig. 4B. This might help to better understand the difference between control group and experimental groups.
6. Fig. 4C: The schematic pictures are not intelligibly defined. Please designate what kind of cells the color blue (THP1 cells?) and the color pink (fibroblast?) represent, respectively.
7. Fig. 4B and 4D: Please provide the representative images of western blot analyses. It is unclear what reference protein (-actin or -tubulin) was used for those western blot analyses in Fig.4B and Fig.4D.
8. Fig. 7D: Please include the images of wild-type mice forepaws with stained -arrestin-1/2 and NFB.
9. Please explain why the lower dose of Ang-(1-7) was more beneficial in ameliorating disease severity and fibrogenic activities than a higher dose.
10. Supplementary Fig. S3A: Please quantify the protein level of collagen I from the western blot image.
11. Supplementary Fig. S3B: It is very confusing to me that no visible staining of collagen fibrils is detected in the photomicrograph of WT mouse. In the manuscript the authors addressed that "Fibrosis progression in RDEB is associated with altered ECM organization rather than increase bulk synthesis of major structural ECM proteins" (Page 8, line 7-8). If this description is true, the collagen fibrils in WT mouse should be stained with picrosirus red and should be visualized under both non-polarized and polarized light. Please include the images of
picrosirus red staining observed under bright light (non-polarized microscopy).

Referee #3 (Remarks for Author):

Bernasconi et al. use tissue proteomics to demonstrate the age-dependent fibrosis of RDEB results from rearrangement of extracellular matrix, not increase in actual structural proteins. Further this ECM rearrangement appears downstream of interactions between inflammatory cells and fibroblasts. Targeted intervention with Ang-(1-7) reduced inflammation, fibrosis progression (both in the skin and other RDEB target organs), and prolonged survival in a hypomorphic murine model of RDEB. This pre-clinical data provides strong support for translation to clinical trials of Ang-(1-7) in RDEB.

Strengths:
(1) Knowledge of clinical RDEB informed well-considered comparisons (time-dependent and site-dependent differences in fibrosis, as well as timing of intervention with Ang-(1-7) at onset of clinical fibrosis of forepaws)
(2) Tissue-specific evaluation of immune infiltrate (more challenging to assay but exceedingly more relevant then systemic blood evaluations)
(3) Greater impact of low dose compared to high dose Ang-(1-7) was consistent across evaluations (in vitro co-culture of monocytes and RDEB fibroblasts compared to in vivo murine studies)
(4) Investigations not restricted to mice, but also completed in human RDEB primary fibroblasts and skin biopsies. Importantly, results were consistent across species, further supporting the validity of the murine RDEB model.
(5) Multiple methods used to reach same conclusions: (a) measures of fibrosis including biometric measure of stiffness and collagen-lattice contraction assays, tissue H&E and IHC (b) protein evaluations (including innate and adaptive immune cell identification) by Western blots, mass spectrometry, flow cytometry, IHC, (c) pathway analysis by cluster analysis and PCA, (c) RNA analyses by RT-PCR, (d) clinical analyses of mice with photography, morphology measurements,

and survival.

The text is well written, easy to understand, and figures clearly displayed with a few exceptions. Access to raw data in tables and ProteomeXchange Consortium is greatly appreciated.

Recommendations / points of clarification:

Major
(1) Some findings appear to be overstated. For example, page 12 "Protracted effects to Ang-(1-7) exposure in cells" text regarding Fig 4D claims long-lasting deactivation of fibrogenic responses yet the data shows only trends. In Fig 4D, the majority of comparisons between in fibronectin, THBS-1 and pSMAD-2/3 between treatment groups are non-significant. Authors could repeat experiments to yield a higher "n" to achieve statistical significance, or report as trends. Phosph-SMAD-2/3 reductions are also not impressive in Fig 7C/FS14. Perhaps these comparisons would be aided by quantification of the Western blot as suggested below (minor #4).

(2) Supplemental tables are currently excel files of raw date. It would be helpful for readers to distill this into accessible tables, perhaps highlighting main effects/observations.

Minor:
(1) Page 9 (end of "Dynamic changes of inflammation during progression of dermal fibrosis" paragraph 1). "Pro-fibrotic priming" misspelled as pro-fibrosiscpriming.
Page 10 (line 5 of "Ang-(1-7) alters inflammatory cell-fibroblast communication). "Regulator" misspelled as refulator.
Period missing at the end of the first sentence on p21.
Comma missing after "AN" in Author contributions, conceptualization.

(2) Materials and Methods. Studies using animals and patient material. p21, line 8 states "Sixteen mice were included." However, just prior to this a total of 43 mice are described. Can you clarify this discrepancy?

(3) Fig 6. Are these analyses following 7 weeks of treatment? This is clearly stated in the figure legend for Fig 7, but absent from Fig 6.

(4) Fig 7C. Is the Western blot photo of beta-arrestin-1/2 cut off at the top? Would it be possible for Fig 7C and E to add densiometric quantifications of the Western blot findings? Some changes are visually quite obvious but others rather subtle.

(5) Fig S8, "-Ang-(1-7)" suggests maybe taking away or inhibiting the renin-angiotensin system. Would suggestion relabeling as "No" or "Absence/Presence". This image of the collagen-lattice contraction is also difficult to visualize, though the dotted outline applied to the photo is helpful.

Point-by-point reply EMM-2021-14392

***** Reviewer's comments *****

Referee #1 (Remarks for Author):

Bernasconi et al's manuscript entitled "Pro-inflammatory immunity drives fibrosis in EB: intervention with Ang-(1-7)" uses both a mouse model and human RDEB cells and tissue to characterise the proteome of progressive RDEB associated fibrosis, to characterise local and systemic inflammation associated with disease progression, and to test the effects of Ang-(1-7) on in vitro cell cultures and in vivo disease.
The Introduction provides a nicely-written background to the problem, and key information needed to understand the rationale to the current work.

Reply: We would like to thank the Reviewer for the supportive comments on our work.

Bottom of page 3, please declare more about the historic proteomic analyses of the RDEB mice, and mid-Page 4, provide the existing background about inflammation in RDEB. Also bottom of Pg3, in reference to the previous TGFb findings, please include that this information relates to work in the mouse model.

Reply: We have now added such information. We did not include it in the first version of the manuscript due to space limitation; nevertheless, we apologize for omitting this information and agree with the Reviewer that the manuscript benefits from these additions.

RESULTS - The longitudinal analysis of the tissue proteome somewhat builds upon the authors' 2015 paper. Perhaps reassuringly the conclusions are the same (inflammation is abundant) but the incremental nature of this should be declared in Intro and Discussion.

Reply: In our study published in EMBO Mol Med 2015 (Nyström et al.,) we did indeed use mass spectrometry-based proteomics to assess the response to losartan in the same RDEB mouse model vis-à-vis control RDEB and WT mice. We analyzed one age – adult 12-week-old mice and one location – protected back skin. In the present study we also use mass spectrometry-based proteomics; however, the questions we are asking and the analyses we are performing are very different.

Here, we analyze natural progression of disease and fibrosis in RDEB by employing mice from different ages representing different disease stages and by using skin from two anatomical regions: protected back skin and mechanically exposed paws. The level of detail and information of disease progression we provide in these analyses and follow-up studies are in our opinion unique and we do not see this as an incremental advance. We agree with the Reviewer that results should be linked more clearly to our previous work in the Introduction and we have done so.

The proteomic data in the supplementary files need improved column headings/annotations (e.g. what are the FC columns - state numerator/denominator and somewhere explain that H are the hypomorphs).

Reply: *We thank the reviewer for the feedback. We updated all supplementary tables. As new software packages were available, we reran analyses, shortened tables to make them easier accessible, and added table headers to clearly explain what is displayed in each column. Re-analyses of data led to minor changes in the shortlisted proteins without affecting the findings and general conclusions. We updated respective figures accordingly.*

The mechanical testing at a single site (i.e. not the back) limits the ability to include the friction-stiffness association in the interpretations in the first paragraph on Pg7.

Reply: We thank the reviewer for this excellent point. We have included stiffness measurements of also back skin from the same mice (Figure 1B). These data clearly support the friction-stiffness association.

Other studies have previously revealed that the protein composition of fibrotic lesions may not include more Collagen I. With the importance placed on this observation in the abstract and final discussion paragraph, please relate it to the literature, and if space allows, discuss any possible limitations of this particular proteomic approach (e.g. extraction method, solubility) that may be influencing this observation.

Reply: *The information that we provide on the establishment and the appearance of the injury-induced fibrosis in RDEB is new and we believe is very important for improved treatment of RDEB and other types of fibroses arising in a similar context.*

The Reviewer is absolutely correct in that although fibrosis is generally linked to excessive accumulation of a collagen-rich extracellular matrix in tissues, it is not limited to accumulation of fibrillar collagen but also other extracellular matrix proteins are involved. We do not claim that we are the first to describe a fibrosis without excessive collagen I deposition, nor do we claim that we are the first to point to heterogeneity of the establishment and the appearance of fibrosis among different fibrotic diseases. We have edited the abstract to state that others are also working on this, and added a reference to the last paragraph of the Discussion, an editorial (Schaefer 2018), which discusses heterogeneity among different types of fibroses and provides references for further reading.

Regarding the limitation of using proteomics. There are as always limitations to rely only on one technique. We have employed multiple complementary analytical techniques, including biochemistry, tissue staining, gene expression analyses and flow cytometry to validate and provide more details to the findings from the proteomics. Prior to proteomics we tested several extraction and fractionation methods. We opted for the most robust approach in our hands: taking entire tissue without any pretreatment/fractionation, grinding it in liquid N_2 and solubilizing all proteins in 4% SDS. These are rather harsh methods and we did not observe visible precipitates after extraction. We realize we had omitted information on the extraction of the samples for

mass spectrometry analysis in the Materials and methods section and have added the following "Tissue was frozen on dry-ice, crushed and tissue homogenate boiled in an SDS-PAGE loading buffer containing 4% SDS with 100 mM Tris ph 7.6, 1 mM DTT and 4 mM Pefabloc (Sigma) under harsh vortexing for 20 min.".

Nevertheless, due to the bottom-up proteomics approach, we might lose highly crosslinked peptide species which could influence protein quantification, i.e. if one sample is highly crosslinked and the other one not. We added a respective statement to the Discussion "Due to the chosen peptide-based, relative protein quantification by MS-based proteomics, we cannot exclude that an altered extent of crosslinking of ECM proteins in the different genotypes affects our quantification. However, as we observed the same trends by western blot approaches, we are confident that our data reflect the in vivo situation."

The inflammation section of the manuscript is clear (pg8-10), well supported by figure 2. However, contextualisation is lacking (e.g. identifying the existing knowledge and gaps in the intro, as well as discussion about how this fits in, for example, with neutrophil targeting in other EB types). The extrapolation to human tissue is a nice addition to this study, although the data largely confirms what is known (inflammatory infiltrate in RDEB skin), and as mentioned previously, it is important to cite these other studies.

Reply: *The part on inflammation, which was guided by the findings from proteomics, shows i) the early appearance of inflammatory macrophages and their persistence in tissue; and ii) in contrast to some other fibrotic diseases, but similar to e.g. muscular dystrophy, limited involvement of increased tissue-repair macrophages.*

In previous studies, various inflammatory cells have been analyzed in skin samples, wounds or sera from donors with RDEB; however, there are limited data on their changes with advancing disease and on their direct correlation to fibrotic changes. We have added the following text and references into the Introduction "RDEB can be considered a wound healing pathology and indeed analyses of skin wounds, dressings and sera from people with RDEB have revealed altered abundance and activity of immune cells and immune mediators, with some studies indicating wound type-dependent changes (Nyström et al, 2015; Cianfarani et al, 2017; Fuentes et al, 2020; Alexeev et al, 2017; Phillips et al, 2020; Huitema et al, 2021)."

However, we believe that in many aspects our study is unique in terms of careful donor selection allowing us to assess inflammation with advancing disease and fibrosis in this rare disease.

In terms of targeting inflammatory cells in other forms of EB. We are aware that neutrophil targeting is being actively pursued in epidermolysis bullosa acquisita, which is an autoimmune disease. Although self-antibodies have been reported against various skin proteins in RDEB, their involvement in disease progression remains unknown, i.e. whether they are bystanders or active drivers. What is clear is that RDEB is driven by friction-induced damage. Therefore, it is challenging to compare treatment of epidermolysis bullosa acquisita with treatment of RDEB. We are aware that other groups are actively working on neutrophil targeting in various genetic EB

forms including junctional EB and RDEB; however, to our knowledge as of June 23 2021, no such studies have been published. As these data are not publicly available we have not been able to review them and can consequently not comment on them or contextualize them in the manuscript.

P10. "kininogen appeared unique" (more explanation required; unique to RDEB? Unique to forepaw in the 0 and 4 week time-points?) Top of P11 - correct the text referring to Fig S7 - this figure does not address Kininogen function. This paragraph requires some clarification.

Reply: *We acknowledge that this was unclearly phrased and that kininogen should have been better introduced. We apologize for this. The segment has now been edited to provide details on why kininogen appeared unique, as well as providing information to it and its biological actions.*

"Among the proteins that were commonly dysregulated in both back skin and paws, kininogen-1 appeared unique by being significantly increased in RDEB mice at both analyzed sites (in 4-week-old and 10-week-old RDEB paws and in back skin from 10-week-old RDEB mice) (Fig EV3). Kininogen-1 is primarily expressed by the liver; its abundance in intact skin is low but notably enhanced after injury (Merkulov et al, 2008; Schremmer-Danninger et al, 2004). It is processed by proteases such plasma kallikrein and neutrophil-derived proteinase 3 into biologically active peptides including the pro-inflammatory, nonapeptide bradykinin (Kahn et al, 2009). Kininogen-1 and its derived peptides are key components of the KKS which is inter-connected with the RAS – counter-regulation follows through joint employment of ACE and receptor heterodimerization (Su, 2013). The RAS is known as a principal regulator of inflammation and tissue repair (Su, 2013; Bernstein et al, 2018)."

2nd half Pg12 - the use of the word "protracted" in the sub-title and text implies that the time-course (of in vitro signalling) was investigated - but 4b is just a single time-point. Its later use (e.g. at the end of the section) implies that 2 days of co-culture is long (protracted), and further suggests that the authors think the response might be delayed rather than just dampened. The use of this word throughout should be reconsidered. Top of Pg13, please state the half-life from this reference. I see that it is likely less than an hour, but the possibility of it triggering a cell differentiation/polarisation event that persists feels distinct from a "protracted" effect.

Reply: *We want to thank the Reviewer for pointing this out. To describe the events in terms of direct actions of Ang-(1-7) as protracted based on the presented results is not correct. The word was wrongly chosen, we think that long-lasting is a more appropriate word to use and have revised the passage accordingly. In addition we state the half-life of Ang-(1-7) (around 30 min) after intravenous administration in humans.*

In the co-culture experiments, one gap seems to be any kind of molecular detail about how the Thp-1 cells are changing in response to the Ang-(1-7) - and indeed how they are first "activated" by the collagen. As much as there is a reference here, it will be important to show a sign of activation triggered by the collagen as stated. And although complete characterisation of the dampening effects of Ang-(1-7) on the pro-

fibrotic actions of the Thp-1 cells may be beyond the scope of this manuscript, it is thought to be important to show "something" is altered by this treatment in the in vitro system. Maybe MHC II plus a few key pro-inflammatory cytokines?

<u>Reply:</u> *Thank you very much for suggesting to support the activation of THP-1 with data. We have now analyzed expression of two key fibroinflammatory mediators – IL1B and IL6 – in THP-1 cells after activation by collagen. Exposure of THP-1 cells to collagen I as performed in the co-culture assays evokes a significant increase in IL1B and IL6 expression (New Appendix Figure S7), thus collagen I activates THP-1 cells.*

Here, Thrombospondin-1 (and Fn and pSmad2/3 and gel contraction) are used as read-outs of fibrosis, but Thrombospondin-1 is not apparent in the proteomic dataset (S1). Also, does the b-arrestin data from the proteomics support the narrative?

<u>Reply:</u> *It is correct that thrombospondin-1 was not detected in the proteomics and that β-arrestin-1/2 was not detected in all samples. Due to the partially stochastic selection process of peptides by data-dependent mass spectrometry, incomplete data matrices, i.e. missing values of proteins, are indeed a known shortcoming of bottom-up MS-based proteomics approaches. As pointed out by the Reviewer, Thrombospondin-1 was not detected; hence, we cannot make any statement based on our proteomics data regarding this protein.*

β-arrestin-1 was at the detection limit, and we only detected it in 6 out of the 18 analyzed samples (max. 2 datapoints per genotype). Thus, unfortunately, also for beta-arrestin we have too little data to generate meaningful conclusions based on our proteomics analyses.

Importantly, our selection of thrombospondin-1 was based on previous work from others and us that has shown that thrombspondin-1 is increased in RDEB. We now provide reference to these publications (Nyström et al. 2015, Atanasova et al. 2019) in the Results section to provide the rationale for choosing to analyze thrombospondin-1.

Thrombospondin-1 and fibronectin are both suitable markers for assessment of fibrosis-reducing effects as they are both transitional extracellular matrix components and activators of pro-fibrotic signaling.

The choice of β-arrestin-1/2 is motivated by it being involved in signaling of Ang-(1-7) and similar peptides, and that its targeting in fibrotic conditions have shown some benefit (Smith & Rajagopal, 2016 and Lovgren et al, 2011), as referenced in the text in the Results and Discussion. Thus, the choice of β-arrestin-1/2 is not based on increased abundance of in RDEB but based on the prospective action of the tested peptide (Ang-(1-7)).

Bottom of Pg13, please add a few additional details to re-explain the receptor-inhibitor pairs, as well as your interpretation of the blots in S11 (their current quality is insufficient for the reader to see - so their improvement would help).

Reply: *We realize that this was abruptly introduced, we have revised the text to provide more information to Ang-(1-7) signaling "The MAS receptor is widely considered as the major receptor for Ang(1-7) but also the angiotensin II type 1 receptor (AT1R) has been implicated in mediating its biological effects (Bernasconi & Nyström, 2018). Studies in RDEBF with losartan and A779 to inhibit AT1R and MAS, respectively, indicated that AT1R appeared important for the deactivating response observed at low Ang-(1-7) concentrations (Appendix Figure S9).*

We also agree with the Reviewer that the Supplementary Figure S12 was busy making it unclear. We have now revised the figure to focus on the discussed, important lowest dose and support the conclusions with quantification of treatment of RDEB fibroblasts from many donors (New Appendix Figure S9).

The in vivo paw measurements and improvements across all organs (Figs 5-7) following low-dose Ang-(1-7) are compelling and of wide interest. That said, in Figure 5B, the statistics are questionable. The detrimental effects of the high dose appear significant, and this will be an important outcome from this work.

Reply: *Reply:* *It may look like differences between high dose of Ang-(1-7) and PBS treatment are dramatic. However, this is due to the early death of a few mice. When statistically tested we do not find a significant difference in survival between those groups, as stated in the figure.*

We have also consulted a biostatistician, Dr. Rudolf Rohr, Department of Biology, University of Fribourg, regarding the calculations. The calculations are correct. He was surprised by the high P value. This might be explained by the duration of the experiment, which was stopped after 7 weeks. A longer observation period i.e. more death events may have changed the significance. However, this was not possible due to the study design and ethical concerns. In the presented data and observation period there is no statistical significant difference in terms of survival between the high dose of Ang-(1-7) and PBS treatment. On the other hand, the low dose of Ang-(1-7) significantly extends survival compared to PBS treatment.

Our proteomics of RDEB mice treated with a high dose of Ang-(1-7) and PBS-treated RDEB mice further supports the limited differences between PBS and Ang-(1-7) high dose, on a molecular level.

Thus, a higher dose of Ang-(1-7) appears not have a deleterious effect; however, the tissue inflammation-reducing and fibrosis-delaying properties seem to be lost.

Also in 5A, is the histological section folded for the low-dose image? It looks strange and distracting enough that it is difficult to comment on cellularity, for example.

Reply: *We agree that the shown section should be replaced and we have done so (Figure 6A).*

Discussion - bottom of pg 17, specify "genetic mouse model".

Reply: *This was unclearly phrased and we apologize for this. Genetic model was intended to refer to RDEB; however, the use of unbiased analyses in the following sentence made the reader think that this would specifically refer to studies on the mouse. We have now revised the sentence "Here, we employed a clinically relevant genetic model predisposed to multi-organ fibrosis – RDEB – to conduct comprehensive analyses of the natural progression of fibrosis. Importantly, in RDEB, conspicuous fibrosis developed without increased deposition of major structural ECM proteins."*

The "quenched inflammatory activity" (pg19) feels exaggerated, again given only MHC II data (gentler language is needed).

Reply: *Quenched has been replaced with subtly dampened.*

To conclude, this is a very broad study containing useful datasets, but the proteomic findings closely replicate the findings of Nystrom et al 2015. There is also some concern about the mechanistically superficial nature of the anti-inflammatory actions of Ang-(1-7). Having been through the paper, revisiting the title and abstract reveals over-statements. The title should be adjusted since this work does not demonstrate the pro-inflammatory immunity drives fibrosis. In the abstract, many adjustments are needed: it currently states a "discovery" of pro-inflammatory immunity associating with fibrosis, although this has been described (perhaps not by proteomics) by many, including these authors in 2015. Similarly, this study is not the first to find that fibrosis is more complex than too much of the most common fibrillar proteins. I agree that targeting inflammatory cell-fibroblast communication seems strategic, but it is not clear that this was the mechanism of action of the Ang-(1-7) here, particularly in the in vivo work in Figures 5-7. The in vivo results in the abstract include an alleviation of inflammation/down-modulation of pro-inflammatory immunity, but that was only based on MHC II and indeed alternative mechanisms could be at play. The second-to-last sentence does not belong in this abstract, without conducting the comparative analysis yourselves (this type of work is indeed being undertaken across the research community).

Reply: *We want to thank the Reviewer for the caution about the title and the abstract. We have changed the title to "Pro-inflammatory immunity supports fibrosis advancement in epidermolysis bullosa: intervention with Ang-(1-7)". We have also significantly revised the abstract following the suggestions by the Reviewer,*

We believe that by using multiple models – human and murine – we provide strong support of the anti-inflammatory actions of Ang-(1-7). However, importantly, as repeatedly pointed out Ang-(1-7) does not solely target inflammatory cells but also fibroblasts. This leads to reduced tissue inflammatory activities and reduction in the progression of multi-organ fibrosis (Figures 6 and 7).

Referee #2 (Remarks for Author):

The data presented in this manuscript "Pro-inflammatory immunity drives fibrosis in epidermolysis bullosa: intervention with Ang-(1-7)" conclusively shows how changes in the ECM organization and inflammation are associated with fibrosis progression in RDEB mice and human RDEB skin samples. The conceptual approach that led to the investigation of Ang-(1-7) as therapeutic agent for RDEB is plausible and the meta-analyses comprising both, human and animal data, are included appropriately. Aside from technical comments and some missing supplementary data (stated below), this paper is well written and of substantial interest for future treatment of RDEB.

Reply: *Thank you very much for the supportive comments on our work.*

Comments:
1. Fig. 1D: The analyses of fuzzy c-means algorithm are not clearly addressed. Please describe in detail the protein expression profiles of each cluster: which clusters represent proteins that are upregulated or downregulated, whereas which clusters represent proteins displaying a bi-model expression pattern.

Besides, what does the y-axis label "fold change" mean? Please define it in the figure legend.

Reply: *We are very sorry for the unclear description of these analyses. Practically to generate the clusters, proteins were shortlisted as being significantly regulated between RDEB and WT samples in minimally one timepoint. Log2 values of average protein abundances in RDEB divided by WT samples were standardized and clustered. Protein profiles show relative alterations of protein abundances over time in RDEB compared to WT samples. To make this clearer we changed the y-axis title to "abundance change" and added following statements to the main text and figure legends:*

Main text:
"To understand the dynamic regulation of the processes related to dermal fibrosis, we performed fuzzy c means clustering. Abundance differences of the significantly changed proteins between RDEB and WT mice were log2-transformed and standardized to follow relative changes of RDEB compared to WT mice. Data were split into six clusters of similar size (Fig 1D), clusters 2 and 3 containing proteins that progressively increase or decrease over time in RDEB mice, respectively. Clusters 1 and 4-6 contained proteins with more complex, bimodal abundance changes. Pathway enrichment analyses performed on the proteins within each cluster revealed a dynamic regulation of inflammation during progression of fibrosis in forepaws (Fig 1D, Dataset EV4). These data reiterated principal association of progressive changes in inflammation during fibrosis establishment in RDEB."

Figure Legend:
"(D) Fuzzy c means clustering of significantly altered proteins in paws of RDEB compared to WT mice to visualize their dynamic regulation during disease progression. Average abundance differences of three biological replicates each (RDEB/WT) were log2 transformed and standardized. Shown are relative abundance

changes over time (0, 4 and 10 weeks). Six clusters were generated and each cluster was analyzed for enrichment of pathways and processes using STRING DB (Szklarczyk et al, 2019). Representative pathways are listed for each cluster. The number of proteins belonging to each pathway is indicated in brackets."

2. Fig. 2: Please provide the representative images of FACS analyses of neutrophils, macrophages and MHC II.

<u>Reply:</u> *We thank the Reviewer for this suggestion and we have provided representative plots of neutrophils, macrophage and MHC II analyses from 10-week-old wild-type and RDEB mice. In order to keep the figure clear we only chose to show this timepoint as this would be most informative in terms of changes in RDEB compared to wild type.*

3. In Fig. 2F the y-axis label of monocyte markers is erroneously indicated. The label "% Ly6G- CD45+ cells" is used for neutrophils, not for monocytes. Please correct it accordingly.

<u>Reply:</u> *We are sorry for this mistake and thank the Reviewer for pointing this out. We have corrected the y-axis.*

4. Fig. 4A: Please provide the quantification of western blot analysis of individual proteins in RDEBF cell lysate.

<u>Reply:</u> *This is an excellent suggestion to support the findings. We have included such quantifications. New Figure 4B.*

5. Fig. 4B: Please include a bar graph of an untreated control group in Fig. 4B. This might help to better understand the difference between control group and experimental groups.

<u>Reply:</u> *We have added such bar graph and we agree with the Reviewer that this has improved the clarity of the data presentation.*

6. Fig. 4C: The schematic pictures are not intelligibly defined. Please designate what kind of cells the color blue (THP1 cells?) and the color pink (fibroblast?) represent, respectively.

<u>Reply:</u> *We apologize for inadvertently omitting the labeling. We have now included the labels and again thank the Reviewer for detecting this oversight.*

7. Fig. 4B and 4D: Please provide the representative images of western blot analyses. It is unclear what reference protein (□-actin or □-tubulin) was used for those western blot analyses in Fig.4B and Fig.4D.

<u>Reply:</u> *We are sorry for not directly having stated this and we have edited the figure legend to provide this information. In addition, we have now provided representative*

blots for both these analyses. Depending on which protein was analyzed β-actin or β-tubulin was used as loading control.

8. Fig. 7D: Please include the images of wild-type mice forepaws with stained □-arrestin-1/2 and NF□B.

Reply: *We agree with the Reviewer that including such stains is informative and we have now added a panel with staining of wild-type paws. To show images acquired at the same session with identical settings we have had to replace all panels (Figure 7D).*

9. Please explain why the lower dose of Ang-(1-7) was more beneficial in ameliorating disease severity and fibrogenic activities than a higher dose.

Reply: *We believe that this is due to the multiphasic dose response to Ang-(1-7) which we can detect both in vitro and in vivo. The mechanistic nature underlying this response is not known but one explanation could be different signaling strength leading to differential activation of mediators downstream of the GPCR. In support of this are observations from the decarboxylated form of Ang-(1–7) - Ala^{1}-Ang-(1–7) in which differential coupling of Gαi and Gαs proteins appears to occur depending on the ligand (peptide) concentration leading to a bell-shaped concentration response of cAMP in endothelial and mesangial cells (Tetzner et al, 2018). Importantly. higher concentration of a ligand has also been reported to lead to lower recruitment of β-arrestin to GPCRs (Hübner et al, 2016).*

We have added the following to the Discussion.
"Both in vitro and in vivo analyses revealed that lower doses of Ang-(1-7) displayed stronger fibrosis-limiting effects than higher doses. We did not further go into the mechanisms behind these dose-dependent differences. However, previous studies on the decarboxylated form of Ang-(1–7) - Ala1-Ang-(1–7) have also shown a bell shaped dose response and alluded to dose-dependent differential coupling of Gαi and Gαs proteins behind this response (Tetzner et al, 2018). Importantly, higher concentration of a ligand has also been reported to lead to lower recruitment of β-arrestin to GPCRs (Hübner et al, 2016)."

10. Supplementary Fig. S3A: Please quantify the protein level of collagen I from the western blot image.

Reply: *We have added such quantification for the collagen I α1 peptide and the α2 polypeptide from five wild-type and five RDEB mouse samples. The quantification could not disclose any significant difference in collagen I abundance between wild type and RDEB.*

11. Supplementary Fig. S3B: It is very confusing to me that no visible staining of collagen fibrils is detected in the photomicrograph of WT mouse. In the manuscript

the authors addressed that "Fibrosis progression in RDEB is associated with altered ECM organization rather than increase bulk synthesis of major structural ECM proteins" (Page 8, line 7-8). If this description is true, the collagen fibrils in WT mouse should be stained with picrosirus red and should be visualized under both non-polarized and polarized light. Please include the images of
picrosirus red staining observed under bright light (non-polarized microscopy).

Reply: *This is an excellent point. We sincerely thank the Reviewer for bringing this up. The previously shown image of a picrosirius red stained forepaw from a wild-type mouse acquired under polarized light was too dark after PDF conversion. We have now replaced the images with new images of picrosirius red-stained forepaws from 10-week-old wild-type and RDEB mice acquired under brightfield and polarized light. In these images there is a clear dermal staining of the wild-type sample when viewed under polarized light. The dermal signal in the RDEB sample is significantly stronger. These data are well in line with the presented quantification in Figure 7B. Importantly, under brightfield no notable differences are seen between wild-type and RDEB mouse forepaws. These data support altered organization and not altered abundance of fibrillar collagens in dermal fibrosis in RDEB.*

Referee #3 (Remarks for Author):

Bernasconi et al. use tissue proteomics to demonstrate the age-dependent fibrosis of RDEB results from rearrangement of extracellular matrix, not increase in actual structural proteins. Further this ECM rearrangement appears downstream of interactions between inflammatory cells and fibroblasts. Targeted intervention with Ang-(1-7) reduced inflammation, fibrosis progression (both in the skin and other RDEB target organs), and prolonged survival in a hypomorphic murine model of RDEB. This pre-clinical data provides strong support for translation to clinical trials of Ang-(1-7) in RDEB.

Strengths:
(1) Knowledge of clinical RDEB informed well-considered comparisons (time-dependent and site-dependent differences in fibrosis, as well as timing of intervention with Ang-(1-7) at onset of clinical fibrosis of forepaws)
(2) Tissue-specific evaluation of immune infiltrate (more challenging to assay but exceedingly more relevant then systemic blood evaluations)
(3) Greater impact of low dose compared to high dose Ang-(1-7) was consistent across evaluations (in vitro co-culture of monocytes and RDEB fibroblasts compared to in vivo murine studies)
(4) Investigations not restricted to mice, but also completed in human RDEB primary fibroblasts and skin biopsies. Importantly, results were consistent across species, further supporting the validity of the murine RDEB model.
(5) Multiple methods used to reach same conclusions: (a) measures of fibrosis including biometric measure of stiffness and collagen-lattice contraction assays, tissue H&E and IHC (b) protein evaluations (including innate and adaptive immune cell identification) by Western blots, mass spectrometry, flow cytometry, IHC, (c) pathway analysis by cluster analysis and PCA, (c) RNA analyses by RT-PCR, (d) clinical analyses of mice with photography, morphology measurements, and survival.

The text is well written, easy to understand, and figures clearly displayed with a few exceptions. Access to raw data in tables and ProteomeXchange Consortium is greatly appreciated.

<u>Reply:</u> *We thank the reviewer for these supportive comments on our work.*

Recommendations / points of clarification:

Major
(1) Some findings appear to be overstated. For example, page 12 "Protracted effects to Ang-(1-7) exposure in cells" text regarding Fig 4D claims long-lasting deactivation of fibrogenic responses yet the data shows only trends. In Fig 4D, the majority of comparisons between in fibronectin, THBS-1 and pSMAD-2/3 between treatment groups are non-significant. Authors could repeat experiments to yield a higher "n" to achieve statistical significance, or report as trends. Phosph-SMAD-2/3 reductions are also not impressive in Fig 7C/FS14. Perhaps these comparisons would be aided by quantification of the Western blot as suggested below (minor #4).

<u>Reply:</u> *We agree with the Reviewer that the statements in regard to the last part of Figure 4 would benefit from being supported by statistically significant data rather than strong tendencies. Toward this end, we have taken the Reviewer's advice and repeated the experiment presented in former Figure 4D, new 4F and G. This has resulted in that the reduction of thrombospondin-1 and pSMAD-2/3 is significant after treatment of only THP-1 cells, or of both THP-1 cells and RDEB fibroblasts; whereas, fibronectin is only significantly reduced upon treatment of both THP-1 cells and fibroblasts. This has led us to add the following to the text.*

"Subsequent western blotting of the RDEBF fraction revealed that Ang-(1-7) priming of THP-1 cells was sufficient to induce long-lasting deactivation of fibrogenic response, as shown by reduction of pSMAD-2/3 and thrombospondin-1 (Fig 4F and G). However, for reduction of fibronectin, treatment of both THP-1 and fibroblasts prior to co-culture was most efficient (Fig 4F and G)."

Quantifications for the blots shown in Figure 7C are presented in Figure EV4. The weak effect on pSMAD-2/3 is consistent with the data in the manuscript and also supported by the quantification for the paws and for eye, esophagus and tongue (Figure EV5). This together with the data from the TGFβ signaling inhibitors that we have used support that Ang-(1-7) to a large part mediates its fibrosis limiting effect through mechanisms outside direct TGFβ signaling targeting.

(2) Supplemental tables are currently excel files of raw date. It would be helpful for readers to distill this into accessible tables, perhaps highlighting main effects/observations.

<u>Reply:</u> *This is a very good comment and we agree that presenting the omics data exclusively as excel files will limit their accessibility. Toward this end, as suggested by the Reviewer we now present data from the GO term analyses as new Table 1.*

Minor:
(1) Page 9 (end of "Dynamic changes of inflammation during progression of dermal fibrosis" paragraph 1). "Pro-fibrotic priming" misspelled as pro-fibrosiscpriming.

Page 10 (line 5 of "Ang-(1-7) alters inflammatory cell-fibroblast communication). "Regulator" misspelled as refulator.
Period missing at the end of the first sentence on p21.
Comma missing after "AN" in Author contributions, conceptualization.

Reply: *Thank you very much for pointing these typos out to us, we have now corrected them.*

(2) Materials and Methods. Studies using animals and patient material. p21, line 8 states "Sixteen mice were included." However, just prior to this a total of 43 mice are described. Can you clarify this discrepancy?

Reply: *This was an unfortunate clerical error. The correct number is 43. We have corrected this.*

(3) Fig 6. Are these analyses following 7 weeks of treatment? This is clearly stated in the figure legend for Fig 7, but absent from Fig 6.

Reply: *We apologize for having omitted this important information from the figure legend. They were analyzed after 7 weeks of treatment. We had added this information to the figure legend.*

(4) Fig 7C. Is the Western blot photo of beta-arrestin-1/2 cut off at the top? Would it be possible for Fig 7C and E to add densiometric quantifications of the Western blot findings? Some changes are visually quite obvious but others rather subtle.

Reply: *The presented β-arrestin membrane had first been probed for another protein and this was the band partially visible at the top. We have replaced the blot with a new blot for β-arrestin for which β-arrestin was probed first. We agree with the Reviewer that some due to the rather subtle changes some difference could be hard to make out in the presented blots. We have therefore in addition to quantifying blots from analyses of multiple mice (Figures EV4 and EV5), replaced some of the images with clearer exposures or blots (7C - β-arrestin-1/2; 7E Eye - αSMA, Thbs-1; 7E Esophagus - pSMAD-2/3, αSMA; 7E Tongue - Fibronectin, Tenascin-C, αSMA, pSMAD-2/3 and their respective loading controls).*

(5) Fig S8, "-Ang-(1-7)" suggests maybe taking away or inhibiting the renin-angiotensin system. Would suggestion relabeling as "No" or "Absence/Presence". This image of the collagen-lattice contraction is also difficult to visualize, though the dotted outline applied to the photo is helpful.

Reply: *Thank you very much for this great comment on how to improve the clarity in the labeling of this figure. We have changed it to No and 1 nM Ang-(1-7). In addition*

we have re-run the contraction assays to obtain better images. The previously presented image has been replaced and since we have repeated the experiment with additional donors we have added more values to the graph which has led to that the significance has changed from P = 0.031 to P = 0.0001.

1st Revision - Editorial Decision 15th Jul 2021

15th Jul 2021

Dear Dr. Nyström,

Thank you for the submission of your revised manuscript to EMBO Molecular Medicine. We have now received the enclosed reports from the referees who had reviewed your original manuscript. As you will see, they are now supportive of publication, and I am therefore pleased to inform you that we will be able to accept your manuscript once the following editorial points will be addressed:

1/ Referees' comments:
Please address the minor comments from referee #3.

2/ Main manuscript text:
- Please address the queries from our data editors in track changes mode in the main manuscript file labelled 'Related manuscript file'. Please use this file for any further modification.
- Please remove the red text, and only keep in track changes the new modifications.
- Please remove the abbreviation list, and instead incorporate the abbreviations in the manuscript text.
- Material and methods:
o Studies using animals and patient material: please clearly indicate "human" or "mouse" origin, and include a statement that the experiments conformed to the principles set out in the WMA Declaration of Helsinki and the Department of Health and Human Services Belmont Report.
o Antibodies: please indicate the dilutions used.
o Cells: please indicate the origin and species of the cells, and whether they were authenticated and tested for mycoplasma contamination.
- Data Availability: Please replace "data and materials availability" by "Data Availability". Thank you for depositing the proteomics data in a public repository, please provide a URL link. Please remove: "The RDEB mouse model can be obtained through an MTA"
- Author contributions: Michael Stumpe is currently missing, please update.

3/ Figures and Appendix:
- Thank you for providing exact p values for significant results. Please also provide exact p values for non-significant results (n.s.). Some people found that to keep the figures clear, providing a supplemental table with all exact p-values was preferable. You are welcome to do this if you want to.
- Please note that Table 1 needs to be editable and in black & white only.
- Please make sure that all figures are correctly referenced in the text. There is a callout for Suppl. Fig. S7 that should be corrected to Appendix Figure S7. Panel 1C of figure EV3 is not called out in the text.
- The dataset legends should be removed from the manuscript and added to the files. Nomenclature in the dataset files should be corrected.
- The Appendix Excel tables should be made Table EV1 and Table EV2.

4/ Checklist:
Section E/12: include a statement that the experiments conformed to the principles set out in the WMA Declaration of Helsinki and the Department of Health and Human Services Belmont Report.

5/ Source Data:

Thank you for providing Source Data. Source Data fig. 2K seems to be mislabeled (2J?). Please check.

6/ Synopsis:
Thank you for providing a synopsis text. Please also suggest a striking image or visual abstract to illustrate your article as a PNG/tiff/jpeg file 550 px wide x 300-600 px high.

7/ As part of the EMBO Publications transparent editorial process initiative (see our Editorial at http://embomolmed.embopress.org/content/2/9/329), EMBO Molecular Medicine will publish online a Review Process File (RPF) to accompany accepted manuscripts.
This file will be published in conjunction with your paper and will include the anonymous referee reports, your point-by-point response and all pertinent correspondence relating to the manuscript. Let us know whether you agree with the publication of the RPF and as here, if you want to remove or not any figures from it prior to publication.
Please note that the Authors checklist will be published at the end of the RPF.

I look forward to receiving your revised manuscript.

Yours sincerely,

Lise Roth

Lise Roth, PhD
Editor
EMBO Molecular Medicine

To submit your manuscript, please follow this link:

Link Not Available

*Additional important information regarding Figures

Each figure should be given in a separate file and should have the following resolution:
Graphs 800-1,200 DPI
Photos 400-800 DPI
Colour (only CMYK) 300-400 DPI"

Figures are not edited by the production team. All lettering should be the same size and style; figure panels should be indicated by capital letters (A, B, C etc). Gridlines are not allowed except for log plots. Figures should be numbered in the order of their appearance in the text with Arabic numerals. Each Figure must have a separate legend and a caption is needed for each panel.

*Additional important information regarding figures and illustrations can be found at https://bit.ly/EMBOPressFigurePreparationGuideline

The system will prompt you to fill in your funding and payment information. This will allow Wiley to send you a quote for the article processing charge (APC) in case of acceptance. This quote takes into account any reduction or fee waivers that you may be eligible for. Authors do not need to pay any fees before their manuscript is accepted and transferred to our publisher.

***** Reviewer's comments *****

Referee #1 (Comments on Novelty/Model System for Author):

These are unchanged from the original review. This is high-quality research, somewhat incremental from the authors' previous work (although, they nicely explained how this study is distinct in the rebuttal), which may have medical impact. The use of the impressively representative mouse model in parallel with human tissue is a strength.

Referee #1 (Remarks for Author):

The authors have done well to address the suggestions and concerns of the reviewers. The paper is now thought to be ready for publication.

Referee #2 (Remarks for Author):

All concerns are addressed properly.

Referee #3 (Comments on Novelty/Model System for Author):

No issues identified, limitations are appropriately disclosed.

Referee #3 (Remarks for Author):

The revisions and response to reviewer's comments clarified a number of points and greatly strengthened the manuscript, leaving no additional scientific comments. Two minor typographical comments:
(1) Pg 7, a full sentence is repeated "Newborn WT and RDEB mice . . . mild disease phenotype at a young age."
(2) Table 2, Italicized sub-labels spell forepaws "forpaws." Additionally unclear what is meant by X-wk-old "up" unless that should read "pup."

2nd Authors' Response to Reviewers **19th Jul 2021**

The authors performed the requested editorial changes.

2nd Revision - Editorial Decision 23rd Jul 2021

23rd Jul 2021

Dear Dr. Nyström,

Thank you for providing the revised files. I am pleased to inform you that your manuscript is now accepted for publication and is being sent to our publisher to be included in the next available issue of EMBO Molecular Medicine!

Please note however that the data (Data Availability section) have to be public before online publication of your manuscript.

Please read below for additional IMPORTANT information regarding your article, its publication and the production process.

Congratulations on your interesting work!

With kind regards,

Lise Roth

Lise Roth, Ph.D
Editor
EMBO Molecular Medicine

Follow us on Twitter @EmboMolMed
Sign up for eTOCs at embopress.org/alertsfeeds

*** *** *** IMPORTANT INFORMATION *** *** ***

SPEED OF PUBLICATION
The journal aims for rapid publication of papers, using using the advance online publication "Early View" to expedite the process: A properly copy-edited and formatted version will be published as "Early View" after the proofs have been corrected. Please help the Editors and publisher avoid delays by providing e-mail address(es), telephone and fax numbers at which author(s) can be contacted.

Should you be planning a Press Release on your article, please get in contact with embomolmed@wiley.com as early as possible, in order to coordinate publication and release dates.

LICENSE AND PAYMENT:

All articles published in EMBO Molecular Medicine are fully open access: immediately and freely available to read, download and share.

EMBO Molecular Medicine charges an article processing charge (APC) to cover the publication costs. You, as the corresponding author for this manuscript, should have already received a quote with the article processing fee separately. Please let us know in case this quote has not been received.

Once your article is at Wiley for editorial production you will receive an email from Wiley's Author Services system, which will ask you to log in and will present you with the publication license form for completion. Within the same system the publication fee can be paid by credit card, an invoice, pro forma invoice or purchase order can be requested.

Payment of the publication charge and the signed Open Access Agreement form must be received before the article can be published online.

PROOFS

You will receive the proofs by e-mail approximately 2 weeks after all relevant files have been sent o our Production Office. Please return them within 48 hours and if there should be any problems, please contact the production office at embopressproduction@wiley.com.

Please inform us if there is likely to be any difficulty in reaching you at the above address at that time. Failure to meet our deadlines may result in a delay of publication.

All further communications concerning your paper proofs should quote reference number EMM-2021-14392-V3 and be directed to the production office at embopressproduction@wiley.com.

Thank you,

Lise Roth, Ph.D
Scientific Editor
EMBO Molecular Medicine

EMBO PRESS

YOU MUST COMPLETE ALL CELLS WITH A PINK BACKGROUND

PLEASE NOTE THAT THIS CHECKLIST WILL BE PUBLISHED ALONGSIDE YOUR PAPER

Corresponding Author Name: Alexander Nyström

Journal Submitted to: EMBO Molecular Medicine

Manuscript Number: EMM-2021-14392

Reporting Checklist For Life Sciences Articles (Rev. June 2017)

This checklist is used to ensure good reporting standards and to improve the reproducibility of published results. These guidelines are consistent with the Principles and Guidelines for Reporting Preclinical Research issued by the NIH in 2014. Please follow the journal's authorship guidelines in preparing your manuscript.

A- Figures

1. Data

The data shown in figures should satisfy the following conditions:

- the data were obtained and processed according to the field's best practice and are presented to reflect the results of the experiments in an accurate and unbiased manner.
- figure panels include only data points, measurements or observations that can be compared to each other in a scientifically meaningful way.
- graphs include clearly labeled error bars for independent experiments and sample sizes. Unless justified, error bars should not be shown for technical replicates.
- if n< 5, the individual data points from each experiment should be plotted and any statistical test employed should be justified
- Source Data should be included to report the data underlying graphs. Please follow the guidelines set out in the author ship guidelines on Data Presentation.

2. Captions

Each figure caption should contain the following information, for each panel where they are relevant:

- a specification of the experimental system investigated (eg cell line, species name).
- the assay(s) and method(s) used to carry out the reported observations and measurements
- an explicit mention of the biological and chemical entity(ies) that are being measured.
- an explicit mention of the biological and chemical entity(ies) that are altered/varied/perturbed in a controlled manner.

- the exact sample size (n) for each experimental group/condition, given as a number, not a range;
- a description of the sample collection allowing the reader to understand whether the samples represent technical or biological replicates (including how many animals, litters, cultures, etc.).
- a statement of how many times the experiment shown was independently replicated in the laboratory.
- definitions of statistical methods and measures:
  - common tests, such as t-test (please specify whether paired vs. unpaired), simple χ2 tests, Wilcoxon and Mann-Whitney tests, can be unambiguously identified by name only, but more complex techniques should be described in the methods section;
  - are tests one-sided or two-sided?
  - are there adjustments for multiple comparisons?
  - exact statistical test results, e.g., P values = x but not P values < x;
  - definition of 'center values' as median or average;
  - definition of error bars as s.d. or s.e.m.

Any descriptions too long for the figure legend should be included in the methods section and/or with the source data.

In the pink boxes below, please ensure that the answers to the following questions are reported in the manuscript itself. Every question should be answered. If the question is not relevant to your research, please write NA (non applicable). We encourage you to include a specific subsection in the methods section for statistics, reagents, animal models and human subjects.

USEFUL LINKS FOR COMPLETING THIS FORM

http://www.antibodypedia.com	Antibodypedia
http://1degreebio.org	1DegreeBio
http://www.equator-network.org/reporting-guidelines/improving-bioscience-research-repor	ARRIVE Guidelines
http://grants.nih.gov/grants/olaw/olaw.htm	NIH Guidelines in animal use
http://www.mrc.ac.uk/Ourresearch/Ethicsresearchguidance/Useofanimals/index.htm	MRC Guidelines on animal use
http://ClinicalTrials.gov	Clinical Trial registration
http://www.consort-statement.org	CONSORT Flow Diagram
http://www.consort-statement.org/checklists/view/32-consort/66-title	CONSORT Check List
http://www.equator-network.org/reporting-guidelines/reporting-recommendations-for-tumo	REMARK Reporting Guidelines (marker prognostic studies)
http://datadryad.org	Dryad
http://figshare.com	Figshare
http://www.ncbi.nlm.nih.gov/gap	dbGAP
http://www.ebi.ac.uk/ega	EGA
http://biomodels.net/	Biomodels Database
http://biomodels.net/miriam/	MIRIAM Guidelines
http://jjj.biochem.sun.ac.za	JWS Online
http://oba.od.nih.gov/biosecurity/biosecurity_documents.html	Biosecurity Documents from NIH
http://www.selectagents.gov/	List of Select Agents

B- Statistics and general methods

Please fill out these boxes (Do not worry if you cannot see all your text once you press return)

1.a. How was the sample size chosen to ensure adequate power to detect a pre-specified effect size?	The calculations for the sample size were based on data of progression and variance from a previous study (Nystrom et al., EMM 2015). Using these data we applied a Log-Rank test using a power of 80% and P < 5% to calculate the sample size.
1.b. For animal studies, include a statement about sample size estimate even if no statistical methods were used.	By a Log-Rank test with a power of 80% and P < 5% as described in 1a.
2. Describe inclusion/exclusion criteria if samples or animals were excluded from the analysis. Were the criteria pre-established?	An ethical exclusion criteria applied; only mice meeting these criterea (expected activity and weight gain) were treated. Among these no mice were excluded. The treatment started at the first sign of toe loss (average 35 days of age for all groups).
3. Were any steps taken to minimize the effects of subjective bias when allocating animals/samples to treatment (e.g. randomization procedure)? If yes, please describe.	At the start of treatment the mice were randomly divided into the three groups; every third mouse was assigned to one of the treatment groups.
For animal studies, include a statement about randomization even if no randomization was used.	Randomization was performed as detailed above.
4.a. Were any steps taken to minimize the effects of subjective bias during group allocation or/and when assessing results (e.g. blinding of the investigator)? If yes please describe.	Due to ethical reasons blinded treatment was not possible. Photographs of the treated mice as well as staining of tissue sections were quantified by a blinded researcher.
4.b. For animal studies, include a statement about blinding even if no blinding was done	Blinding during treatment was not possible, since for ethical reasons it had to be known which treatment the mice received. Quantifcation of staining intensities and toe length was performed by a blinded researcher.
5. For every figure, are statistical tests justified as appropriate?	Yes, all figures contains clear information of the statisitcal tests used. The selection of statistical test used was dependent on the nature of data analyzed and the comparions that were made.
Do the data meet the assumptions of the tests (e.g., normal distribution)? Describe any methods used to assess it.	Yes, they do. The datasets were tested for normality and equal variance using Shapiro-Wilk and F tests.
Is there an estimate of variation within each group of data?	This is provided for all presented data either as standard error of the mean or standard deviation.
Is the variance similar between the groups that are being statistically compared?	Yes, this was first assessed using F tests.

C- Reagents

6. To show that antibodies were profiled for use in the system under study (assay and species), provide a citation, catalog number and/or clone number, supplementary information or reference to an antibody validation profile. e.g., Antibodypedia (see link list at top right), 1DegreeBio (see link list at top right).	We list the catalogue number and clone number (when applicable) for all antibodies used: primary antibodies: rabbit anti-pSMAD-3 (S423/425) (ab52903), rabbit anti-fibronectin (ab2413), mouse anti-IL6 (ab9324), rabbit anti-IL1-β (ab9722), rabbit anti-kininogen 1 (ab175386), rabbit-anti ACE-2 (ab15348), rabbit anti-periostin (ab14041), rabbit anti-β-tubulin (ab6046) all from Abcam; rabbit anti-pSMAD-2 (Ser 465/467) (138D4), rabbit anti-NF-kB p65 (D14E12), rabbit anti-β-arrestin1/2 (D24H9), rabbit anti-pAkt (Ser 473) (D9E) from Cell Signaling; rat anti-mouse tenascin-C clone 578 from R&D Systems); CD68 mouse monoclonal Antibody (KP1), CD3 ε mouse monoclonal Antibody (UCH-T1); mouse anti-α-smooth muscle actin (clone 1A4) and mouse anti-activated MAP kinase (diphosphorylated Erk-1/2) from Sigma; mouse anti-thrombospondin-1 clone A6.1 and rat anti-mouse syndecan-1 (clone DL-101) from ThermoFisher Scientific, rabbit anti-collagen I (Origene); secondary antibodies: HRP-conjugated goat anti-rabbit (KPL, cat. Nr. 474-1506), HRP-conjugated mouse anti-β-actin (C4) (sc-47778) (Santa Cruz Biotechnology), HRP-conjugated goat anti-mouse (Calbiochem, cat. Nr. 401253-2ML), HRP-conjugated goat anti-rat (Jackson Immuno Research, cat. Nr. 112-035-167, Alexa Fluor 594 goat anti-rat IgG (Invitrogen, ref. A11007) and Alexa Fluor 488 goat anti-rat IgG (Invitrogen, ref. A11006).<br><br>For flow cytometry analyses: anti-CD45 APC/Cy7 clone 30-F11 (BioLegend, ref. 103116), anti-CD11b V450 clone M1/70 (BDHorizon, ref. 560455), anti-CD11b FITC clone M1/70 (BD Biosciences ref. 557396), anti-F4/80 PerCP/Cy5.5 clone BM8 (BioLegend, ref. 123128), anti-mouse Ly6G Pe/Cy7 clone 1A8 (BioLegend, ref. 127618), anti-CD38 PE clone 90 (Invitrogen, ref. 12-0381-82), anti-Ly6C PeLy7 clone 1 HK1.4 (BioLegend ref. 128017), anti-CD62L biotinylated clone MEL14 (BD Biosciences ref. 104403) stained together with APC-streptavidin, anti-MHCII eFluor 450 clone AF6-120.1 (Thermo Fischer Scientific ref. 48-5320-82), anti-Egr2 APC (eBioscience, ref. 17-6691-82), anti-NK 1.1 APC clone PK136 (BioLegend), anti-CD3ε PE clone eBio500A2 (eBioscience), anti-CD8 PeCP clone 53-7.6 (BioLegend), anti-CD4 BrilliantViolet 421 clone GK 1.5 (BioLegend).
7. Identify the source of cell lines and report if they were recently authenticated (e.g., by STR profiling) and tested for mycoplasma contamination.	We routinely screen our cell culture facility for mycoplasma contamination. All tests during the time the work for this study was performed were mycoplasma negative. Human RDEB fibroblasts were primary cells. The THP-1 cell line was used and was purchased from ATCC.

* for all hyperlinks, please see the table at the top right of the document

D- Animal Models

8. Report species, strain, gender, age of animals and genetic modification status where applicable. Please detail housing and husbandry conditions and the source of animals.	Both male and femle mice were used-PBS (16 mice, 11 females and 5 males); 0.1 mg Ang-(1-7) /kg body weight (12 mice, 6 females and 6 males); 1 mg Ang-(1-7) /kg body (15 mice, 10 females and 5 males). The collagen VII hypomorphic mice – RDEB mice were kept on mixed C57BL/6 129sv background. The generation of the mice has been previously described (Fritsch et al, 2008) and the mouse model has been extensively characterized . The mice were kerpt in a pathogen-free facility at the University of Freiburg (CEMT). Mice used for the expermiments were generated by in-house breeding. Mice were given food and water ad libitum and had access to a special soft food diet (Fritsch et al, 2008, Nystrom et al, 2013).
9. For experiments involving live vertebrates, include a statement of compliance with ethical regulations and identify the committee(s) approving the experiments.	Studies using mice were approved by the regional review board (Regierungspräsidium Freiburg, Freiburg, Germany; approval numbers 35/9185.81/G15-140, G14-93, G10-118).
10. We recommend consulting the ARRIVE guidelines (see link list at top right) (PLoS Biol. 8(6), e1000412, 2010) to ensure that other relevant aspects of animal studies are adequately reported. See author guidelines, under 'Reporting Guidelines'. See also: NIH (see link list at top right) and MRC (see link list at top right) recommendations. Please confirm compliance.	The study was designed to comply with the ARRIVA guidelines (Kilkenny et al, 2010).

E- Human Subjects

11. Identify the committee(s) approving the study protocol.	The study using patient-derived material was approved by the ethics committee of the University of Freiburg (approval no. 318/18).
12. Include a statement confirming that informed consent was obtained from all subjects and that the experiments conformed to the principles set out in the WMA Declaration of Helsinki and the Department of Health and Human Services Belmont Report.	The patients gave informed consent for use of the materials for research, and the study was approved by the ethics committee of the University of Freiburg (approval no. 318/18) and was performed in agreement with the principles of the Declaration of Helsinki and the Department of Health and Human Services Belmont Report.
13. For publication of patient photos, include a statement confirming that consent to publish was obtained.	Not applicable.
14. Report any restrictions on the availability (and/or on the use) of human data or samples.	Not applicable.
15. Report the clinical trial registration number (at ClinicalTrials.gov or equivalent), where applicable.	Not applicable.
16. For phase II and III randomized controlled trials, please refer to the CONSORT flow diagram (see link list at top right) and submit the CONSORT checklist (see link list at top right) with your submission. See author guidelines, under 'Reporting Guidelines'. Please confirm you have submitted this list.	Not applicable.
17. For tumor marker prognostic studies, we recommend that you follow the REMARK reporting guidelines (see link list at top right). See author guidelines, under 'Reporting Guidelines'. Please confirm you have followed these guidelines.	Not applicable.

F- Data Accessibility

18: Provide a "Data Availability" section at the end of the Materials & Methods, listing the accession codes for data generated in this study and deposited in a public database (e.g. RNA-Seq data: Gene Expression Omnibus GSE39462, Proteomics data: PRIDE PXD000208 etc.) Please refer to our author guidelines for 'Data Deposition'.<br><br>Data deposition in a public repository is mandatory for:<br>a. Protein, DNA and RNA sequences<br>b. Macromolecular structures<br>c. Crystallographic data for small molecules<br>d. Functional genomics data<br>e. Proteomics and molecular interactions	The mass spectrometry proteomics data have been deposited to ProteomeXchange Consortium via the PRIDE partner repository with the dataset identifiers PXD019957 and PXD019929.
19. Deposition is strongly recommended for any datasets that are central and integral to the study; please consider the journal's data policy. If no structured public repository exists for a given data type, we encourage the provision of datasets in the manuscript as a Supplementary Document (see author guidelines under 'Expanded View' or in unstructured repositories such as Dryad (see link list at top right) or Figshare (see link list at top right).	See above.
20. Access to human clinical and genomic datasets should be provided with as few restrictions as possible while respecting ethical obligations to the patients and relevant medical and legal issues. If practically possible and compatible with the individual consent agreement used in the study, such data should be deposited in one of the major public access-controlled repositories such as dbGAP (see link list at top right) or EGA (see link list at top right).	Not applicable.
21. Computational models that are central and integral to a study should be shared without restrictions and provided in a machine-readable form. The relevant accession numbers or links should be provided. When possible, standardized format (SBML, CellML) should be used instead of scripts (e.g. MATLAB). Authors are strongly encouraged to follow the MIRIAM guidelines (see link list at top right) and deposit their model in a public database such as Biomodels (see link list at top right) or JWS Online (see link list at top right). If computer source code is provided with the paper, it should be deposited in a public repository or included in supplementary information.	Not applicable.

G- Dual use research of concern

22. Could your study fall under dual use research restrictions? Please check biosecurity documents (see link list at top right) and list of select agents and toxins (APHIS/CDC) (see link list at top right). According to our biosecurity guidelines, provide a statement only if it could.	No, it does not fall under these restrictions.
